# Supplementary material for: A stakeholder engagement strategy for an ongoing research program in rural dementia care: Stakeholder and researcher perspectives
Source: PLoS One. 2022 Sep 22;17(9):e0274769. doi: 10.1371/journal.pone.0274769 (PMC9499231; doi:10.1371/journal.pone.0274769)
Supplement: S1 Table — (PDF) [file pone.0274769.s001.pdf]

S1 Table. Description of Summit components and their purpose

Component	Description	Purpose
**Planning**	- Planning Committee includes RaDAR researchers, trainees, staff<br>- Annual Summit evaluation data helps inform choice of presenters, topics, and organizational aspects<br>- The provincial Alzheimer Society provides input into the program and coordinates participation of people living with dementia and family members<br>- Data from evaluation forms and RaDAR team observations are used for Summit planning	- Summit Planning Committee holds debriefing meeting following each Summit to review collated stakeholder evaluations and make plans for the next Summit. The Committee meets again mid-year and more frequently closer to Summit to make more detailed plans<br>- Ensures stakeholder recommendations are used to guide planning<br>- Draws on RaDAR's long-standing relationship with the Alzheimer Society to identify and connect with individuals living with dementia and families<br>- Applying stakeholder recommendations demonstrates respect for their time and commitment to attend Summit; shows we value their input; maximizes repeat participation by ensuring needs/preferences are met; ensures Summit is meaningful and provides value for time as presenters via presentation and technology support
**Stakeholder Participants**	STAKEHOLDERS<br>- Individuals living with dementia and family members<br>- Health care providers and administrators<br>- Alzheimer Society staff and leadership<br>- Provincial Ministry of Health<br>- others<br>RESEARCHERS AND TRAINEES<br>- RaDAR team members and trainees	- Participation is by invitation vs. open registration to ensure representation of key stakeholder sectors and because RaDAR covers all costs. Additional requests to attend are accommodated as budget permits. The size of the Summit has gradually increased<br>- Including stakeholders representing a variety of groups and experiences increases diversity of discussions and opportunities to establish meaningful connections. Expansion to additional groups (e.g., Ministry of Health) resulted from feedback at early Summits<br>- Trainees receive an opportunity to engage directly with stakeholders in a participatory research event

	- non-RaDAR researchers and trainees from the province, Canada, international (keynotes)	- Alzheimer Society involvement exposes Society staff to stakeholder needs, current research, and community initiatives; gives stakeholders the opportunity to meet and engage with Society staff<br>- Ministry involvement is essential for sharing the latest research with decision-makers responsible for health service delivery policies
**Opening reception and Poster Evening**	- 6:30 – 9:00 PM<br>- Appetizers and cash bar provided	- Start time allows most rural stakeholders to travel after work<br>- Providing food supports the social aspect of the informal evening
Poster session	- Interactive session features 20-25 posters by RaDAR and non-RaDAR researchers, trainees, and stakeholders<br>- Includes research ideas and projects in progress and completed<br>- Booklet with reproductions of posters available online in advance	- Non-RaDAR posters show the range of rural dementia research underway throughout province, Canada, and internationally<br>- Trainee poster presentations support capacity-building in dementia care and research<br>- Non-RaDAR affiliated stakeholder posters and displays focused on programs and services and offer solutions to challenges faced in rural communities<br>- Posters are left on display on second day; based on stakeholder feedback, to allow more time for viewing
Brief education presentation	- Educational talk by RaDAR member and dementia care specialist focuses on assessment and management of dementia	- Provides an opportunity for stakeholders without clinical expertise to learn more about Alzheimer's disease and other dementias<br>- This component is consistently highly rated in participant evaluations
Trainee poster prize awards	- Prizes awarded to top 3 trainee posters; the competition is adjudicated by researchers and stakeholders and supported by a Family Trust Fund	- Incentivizes trainees to participate in poster session and recognizes innovative research<br>- The use of the Family Trust Fund for prizes allows an opportunity for RaDAR to publicly acknowledge philanthropy
**Summit Day**	- 8:30 AM to 4:00 PM	- End time allows most rural stakeholders to travel home

Introductory presentation by RaDAR Lead	- Presentation includes current dementia statistics, history of RaDAR program, and overview of current RaDAR research projects	- Statistics and RaDAR history offer context and orientation for new participants, and underscore the need for rural dementia research<br>- Overview of current research provides evidence of the team's active research program; highlights strengths of rural communities
RaDAR highlights panel	- Brief presentations of current research projects by RaDAR members	- Demonstrates to stakeholders that rural issues in dementia care are a priority for many researchers in the province<br>- Since 2015, the panel has included updates on the implementation of Rural PHC Memory Clinics, intended to show our progress in spreading and sustaining the clinics and to encourage stakeholders to advocate for clinics in their communities
Keynote	- Presentation by a national or international research collaborator	- National/international speakers demonstrate and strengthen collaborations with leaders in dementia research<br>- Builds stakeholder capacity by increasing exposure to international research and rural dementia care issues
Small group session	- This is the core engagement activity at Summit (2 hours minimum). One RaDAR member leading a project presents research in development, in progress, or completed; followed by a small group and large group discussion.<br>- Table 2 outlines the session focus by year and the outcomes	- Stakeholders are assigned to groups of 8-10 people to provide recommendations on 3-4 a priori questions designed by the lead researcher, to identify/refine research questions, plan research design, interpret findings (depending on stage of the project). Having the groups facilitated by RaDAR researchers/trainees provides opportunity to enhance facilitation skills and build relationships with stakeholders. Note-takers report back to the larger group and provide detailed notes for the lead researcher<br>- RaDAR team members are dispersed among stakeholder tables as a strategy for engagement that was recommended in the evaluations<br>- The session is the core Summit activity, providing an opportunity to seek direction from a diverse range of

		stakeholders, increasing the appropriateness, relevance, and sustainability of RaDAR projects
Non-RaDAR highlights panel	- Brief presentations on current research projects by non-RaDAR researchers	- Increases stakeholders' awareness of research outside of the RaDAR program<br>- Featuring external researchers demonstrates our readiness to collaborate and strengthens existing collaborations
On the Radar panel	- Presentations of stakeholder-led dementia initiatives	- Raises awareness of grassroots initiatives developed and implemented by stakeholders in local communities, to showcase community initiatives and promote transfer to other settings<br>- Community initiatives developed as a result of attending Summit are highlighted, demonstrating the feasibility of adapting programs to address local needs
Alzheimer Society update	- Alzheimer Society leadership gives overview of client and community statistics (e.g., number receiving services), current program and services, and future initiatives	- Raises awareness of Society's services offered in communities across the province, emphasizing the benefits and broad reach of a provincial non-profit advocacy and service organization
Lived experience panel	- Presentation by person(s) living with dementia and care partners	- Actively listening to and respecting the views of individuals with lived experience is essential for meaningful engagement<br>- Lived experience presentations identify service gaps, counter myths, and may inform decision-making of policy-makers and stakeholders
Breakfast, lunch, and coffee breaks	- Breaks for refreshment and informal networking time	- Adequate time for breaks and meals fosters collaborations and is valuable for informal networking between participants who may see one another infrequently outside of Summit
Evaluation	- Stakeholders complete a paper-based evaluation form at the end of the day that includes rating scales and open-ended	- Stakeholder feedback is critical to planning future Summits<br>- Time for completing evaluations is included in the agenda; completed forms are gathered as stakeholders leave and their

	questions exploring their perspectives of the poster evening and Summit day	names are entered into a draw for a gift, to increase response rate and acknowledge their time for completing the evaluation
Funding	- There is no cost to attend. Research funding initially supported Summit. A trust fund from a local family now provides funding. For those unable to attend without travel support, RaDAR will cover mileage and hotel	- Our aim is to keep the Summit as accessible as possible.<br>- Given the size of the province (1225 kms north to south and 630 kms across at the southern border), the majority of stakeholders have to travel long distances and stay overnight after the Poster Session
Venue	- Located on the city outskirts in unique provincial museum that preserves history of the economic and cultural development of the province. Extensive free parking available	- The Summit was initially held in a hotel in the city downtown. Although amenities and service were excellent, parking was limited and costly. Parking is essential because there is no rural public transportation available. The lower cost of the current venue allowed us to increase the number of participants. Access to the museum allows participants to mingle and socialize during breaks.
Materials	- The agenda, brief biographies and photos of attendees, slide presentations, and poster are available online prior to the event	- Stakeholders appreciate having materials in advance and able to follow slide shows on their devices during the presentations<br>- Biographies (including photos and contact information) support participant networking efforts during and after Summit meeting<br>- Previously a binder with meeting materials was provided, but the majority of participants agreed to “going green” in 2015
Photography	- A professional photographer captures pictures of presenters and attendees who want a photograph	- High-quality images are used for the Summit Report, RaDAR website, and newsletter, and are shared with stakeholders who requested photos. Capturing the event in photographs is highly valued by participants; having a photographer responsible for this allows RaDAR team members to fully participate in Summit activities
