# Supplementary material for: A stakeholder engagement strategy for an ongoing research program in rural dementia care: Stakeholder and researcher perspectives
Source: PLoS One. 2022 Sep 22;17(9):e0274769. doi: 10.1371/journal.pone.0274769 (PMC9499231; doi:10.1371/journal.pone.0274769)
Supplement: S2 Table — (PDF) [file pone.0274769.s002.pdf]

Supporting Information File 3

Example of Summit small group engagement sessions

The small group engagement sessions at Summits 5-7 demonstrate how stakeholders shaped three stages of a project aimed at conducting the first-ever evidence-based provincial-level analysis of gaps between actual and best dementia care practices in the province. This *Dementia Gap Analysis* study consisted of three components:
(1) Synthesis of best practices, (2) analysis of linked administrative data bases, and
(3) environmental scan/inventory of dementia-related services and resources available along the continuum of care across the province.
The project was a collaboration between RaDAR and the provincial Health Quality Council, an independent organization supporting health system improvement by partnering with health organizations, government, and community organizations in the province.

Summit 5 (2012)
An overview of the goals of the Gap Analysis project was presented, followed by a small-group interactive session focused on the *environmental scan* component, which was aimed at determining availability and access to dementia-related services within health regions in the province. In the small groups Summit stakeholders reviewed a draft checklist of services that was developed from input from an earlier provincial consultation, and then made recommendations for services and strategies for inclusion. A moderated discussion followed with each group reporting back and further refining the checklist. These recommendations were incorporated into the design of the environmental scan. This work was instrumental in identifying services and strategies that are most important for persons living with dementia and families, and health care professionals on the front lines of service delivery.

Summit 6 (2013)
Preliminary findings were presented from the *environmental scan* of available dementia-related services and the *administrative health data analysis* related to dementia incidence and prevalence in rural vs. urban areas of the province. A moderated discussion followed to explore interpretations of the results of the two studies that were subsequently used in the *Dementia Gap Analysis* Report.

Summit 7 (2014)
RaDAR team members and the lead Health Quality Council analyst presented an overview of a draft version of the *Dementia Gap Analysis* project. The findings of the three study components and draft recommendations were presented. In the small-group session that followed, each group was asked to identify and rank their top five recommendations, and suggest action plans for each. A moderated discussion was then held with each group reporting back. The final task was to develop a list of 10 recommendations from those developed in the small groups. Following a lively discussion and debate, a final list of 10 recommendations was agreed on, that included policy, research, and knowledge translation and exchange recommendations for the project report.
