# Supplementary material for: A stakeholder engagement strategy for an ongoing research program in rural dementia care: Stakeholder and researcher perspectives
Source: PLoS One. 2022 Sep 22;17(9):e0274769. doi: 10.1371/journal.pone.0274769 (PMC9499231; doi:10.1371/journal.pone.0274769)
Supplement: S3 Table — (PDF) [file pone.0274769.s003.pdf]

**S4 Table. How the Summit differs from a typical conference**

| **Summit** | **Typical conference** |
|---|---|
| -Invited event to ensure representation and mix of key stakeholder sectors; additional requests are accommodated as funding and space permit. | -Open registration to anyone interested. |
| -Location is static so that participants are aware of consistent location and can plan accordingly. | -Conference locations typically rotate around the country, which may limit access depending on location; costs are variable depending on location. |
| -No cost to attend; all meals provided; free parking. We apply for meeting grants to help with costs or fund from existing research grants so that no fees are charged to attend and the event is accessible to all. Current funding is from a family trust fund. | -Costs can be high with registration fees, meals, and parking, limiting attendee participation/representation to those with means to attend. |
| -Smaller group is more intimate. Stakeholders spend the evening and full day together in a plenary session; no break-out sessions except for the small group engagement activity where at least one RaDAR member is assigned to each group, with reporting back to the large group. Spending the majority of the event in a plenary session allows everyone to hear everything together; people get to know each other better and become more comfortable with each other. | -Generally large events with multiple concurrent sessions. |
| -The program includes presentations by researchers, persons living with dementia, caregivers, stakeholders. | -Majority of presentations are by researchers. |
| - Summit includes a cross-section of academic knowledge plus practical knowledge that stakeholders can apply in their rural setting. | -Most conferences have a research focus only. |
| -Summit is focused on rural/remote dementia; most stakeholders have rural interest. Session topics are selected based on relevance to rural dementia care and interests/needs of rural stakeholders. We ask for topic suggestions in the annual evaluations. | -Most conferences include a broader range of topics and sessions. The program is developed based on abstract submissions made by researchers. |
| -Summit is designed to accommodate rural people including start and end times, provision of full meals and snacks, coverage of travel/hotel costs for persons living with dementia and | -A typical conference is not as tailored to meet the needs of a specific group of participants. |

| | |
|---|---|
| caregivers, and those with no other means to attend. We relocated from a downtown hotel to a rural history museum with ample free parking on outskirts of the city. We link persons living with dementia with a contact person assigned to provide support as needed. | |
| -Summit is set up to optimize interaction and engagement (small group session is the core engagement activity); longer breaks, wine and cheese poster evening, document with participant bios and photos helps stakeholders get to know each other and supports post-Summit connections. | -Most conferences include engagement opportunities for researchers (opening receptions, poster sessions) but stakeholder engagement is not the focus. |
| -A detailed Summit report is sent to all stakeholders, with a focus on continued engagement. | -Post-conference follow-up may be minimal |
| -Over time a community of practice has developed where stakeholders and researchers meet regularly to learn and work together on issues of common concern. | -Conferences have regular attendees but more turnover; typically large events, not focused on collaborating to address a particular topic area. |
