# Supplementary material for: A stakeholder engagement strategy for an ongoing research program in rural dementia care: Stakeholder and researcher perspectives
Source: PLoS One. 2022 Sep 22;17(9):e0274769. doi: 10.1371/journal.pone.0274769 (PMC9499231; doi:10.1371/journal.pone.0274769)
Supplement: S1 File — (PDF) [file pone.0274769.s004.pdf]

S2 Table. Summit interactive small group session focus and purpose

Summit Number (Year)	Focus of Interactive Small Group Session	Purpose
Summit 1 (2008)	-A pre-Summit survey of stakeholders identified priority dementia care issues<br>-At Summit, stakeholders identified research most relevant to each priority	Informed priorities and planning for foundational RaDAR projects
Summit 2 (2009)	Review research priorities identified at previous Summit, update on developing projects, and time for stakeholders to meet with project leads regarding project quality, feasibility, and relevance	The discussion informed ongoing development and implementation of projects emerging from the Summit 1 priority-setting process
Summit 3 (2010)	Overviews of team research projects discussed at Summit 2, followed by roundtable meetings where stakeholders could engage with each project lead	Opportunity for stakeholders to re-engage with project leads to further develop projects they identified as priorities at previous Summit
Summit 4 (2011)	Review October 2011 Provincial Stakeholder Consultation that identified priorities for a team grant in rural community-based primary health care	Guided development of the CIHR team grant application, including refining research questions and methods
Summit 5 (2012)*	Develop methods for an environmental scan of rural dementia services, as part of a baseline gap analysis study of dementia care in the province	The resulting recommendations were used to plan study methods for the province-wide study
Summit 6 (2013)*	Review and discuss findings of the environmental scan developed at previous year's Summit	Stakeholders engaged in interpreting results of a study they helped to design
Summit 7 (2014)*	Rank draft recommendations from the provincial Gap Analysis study and develop an Action Plan	Stakeholders developed the final recommendations for the Gap Analysis Report
Summit 8 (2015)	Design adaptations to a decision support tool for dementia diagnosis and management (based on Canadian Consensus Guidelines), to fit rural primary health care teams	Recommended adaptations to the decision support tool were incorporated in design of rural primary health care memory clinics implemented in the partner Health Region
Summit 9 (2016)	Discuss implications of a study of health service use by individuals with dementia; plan next steps in the research project	Generated ideas for future RaDAR research evolving from health service use study
Summit 10 (2017)	Reflect on the role of the Summit over the previous decade; develop a vision for the future Summits	Direction for planning future Summits (e.g., presentation topics, stakeholder groups to

		include, recommendations to keep current venue, format, and small-group engagement sessions
Summit 11 (2018)	Rank interventions to include in a research proposal for a new team project	Guidance used to revise and refine grant proposal prior to submission
Summit 12 (2019)	Provide direction on a grant application to address the need for timely diagnosis in rural communities	Session provided guidance on project goals and design for the grant application

*See Box 1 for more detail on the small-group engagement sessions at Summits 5-7.
