# Supplementary material for: A stakeholder engagement strategy for an ongoing research program in rural dementia care: Stakeholder and researcher perspectives
Source: PLoS One. 2022 Sep 22;17(9):e0274769. doi: 10.1371/journal.pone.0274769 (PMC9499231; doi:10.1371/journal.pone.0274769)
Supplement: S2 File — (PDF) [file pone.0274769.s005.pdf]

CIHR Applied Chair in Health Services & Policy Research:
Decision-Makers' Summit
November 21st, 2008

EVALUATION RESULTS

Were you able to attend the **Reception & Poster Session** last night? Yes = 27 No = 4

If Yes, did the event:

a) Provide you with an opportunity to **learn** about what has been happening with the Rural and Remote Memory Clinic Research? Yes = 26 No = 1 (already knew about it)

b) Provide you with an opportunity to **meet** Research Team members you had not met before? Yes = 27 No = 0

b) Provide you with good **value** for your time (e.g., worth travelling the night prior to the meeting)? Yes = 25 No = 0

Comments on the Reception & Poster Session:

- Very informative
- Very interesting. I would have appreciated having the presenters there to talk about their poster. Some were but not all
- Very Nice!
- I learned about a number of research projects I was not aware of
- Very interesting to see what research has already been done
- Well-organized
- Good to see that the RRMC project can generate a tremendous amount of research projects that involve collaboration from individuals with different academic backgrounds
- Very informative, nice meeting new people who face the same challenges as us
- Very informative & interesting. Good to see all the work being done to address this issue
- Well organized. Would like a copy of the poster presentations for follow up.
- The format was excellent particularly because of the interest level (passion) of the researchers/people there. Food great.
- Very informative. The casual setting was appreciated.
- Good venue to meet people we haven't met
- Lots of fun and good exchange of information
- A wonderful networking opportunity. I don't always get a chance to attend all NET related activities so it provided me with an opportunity to catch up with what people are doing
- Great information
- I liked the chance to connect the night before. It then lets us "get to work" on the Friday
- Lots of overlap between posters from memory clinic. Layout with posters at circumference was great. Abstracts of posters might be helpful in meeting package.

Today's Summit - Please rate your satisfaction with the following *(check appropriate box)*:

	Very Satisfied	Somewhat Satisfied	Could be Better	Definitely not Satisfied
**Time Allotment for Agenda Items**	30	2		
**Time Allotment for Breaks**	29	1	1 (shorter for shorter day)	
**Venue of Summit**	27	2	2	
**Flow of Day's Activities**	30	2		
**Opportunity to voice your opinions**	29	1		
**Accomplishment of Day's Objectives**	24	5		

It was worth my time to attend and participate in this Summit *(please circle one):*

Strongly Agree = 22 Agree = 9 Neither Agree/Disagree=0 Disagree=0 Strongly Disagree=0

What, if anything, should be done differently for the next Decision-Makers' meeting?

- More detailed agenda
- Please provide microphone for speakers (good in PM). Larger
- October, September, or Jan – April are preferred meeting months
- Binder should have a content page. Binder tabs are not properly placed.
- Perhaps a guest speaker
- Meeting at the host hotel instead of a different venue.
- I would appreciate the notes from the breakout groups (summary) (Xxx)
- Perhaps ½ longer so we could have participated in 2 groups. Hard to decide
- Clear focus in small groups
- At the next meeting it would be great to have an update to see if any proposals were pursued and the results
- Great event. Very meaningful to have front-line staff, family, managers and government all in one room.
- A bit long for unstructured discussions.
- I think it was a great opportunity to discuss and share issues. Great information presented. Important to be part of research project and to get our concerns addressed and talked about. I think process is very effective. No changes.
- I really appreciated hearing from the range of "positions": researcher, policy, SaskHealth, frontline, families
- Better acoustics to facilitate discussion. Long lead time for planning. Subgroup discussions ?
- Would like access to all of the selected research team publications on the purple sheet in the handout.

How often should the Decision-Makers meet face-to-face? *(please circle one):*

Quarterly = 3 Twice a Year = 10 Yearly = 18

By what other means would you find useful to communicate with the team? *(check all that apply)*

- emailed newsletters/updates=23
- videoconferencing (e.g. Telehealth) = 16
- teleconferences = 12
- interactive online meetings (e.g. WebEx; Illuminate) = 12
- online community (e.g. Sharepoint; Timeless) =8

Knowledge Network in Rural and Remote Dementia Care
2nd Annual Summit – Xxx
October 29th & 30th, 2009

EVALUATION FORM RESULTS

Were you able to attend the Reception & Poster Session last night? ☐Yes **(19)** ☐No **(2)**

If yes, did the event:

		Yes	No
a)	Provide you with an opportunity to **learn** about research and projects that are current and relevant to rural and remote dementia care in Saskatchewan?	☐Yes **(19)**	☐No **(0)**
b)	Provide you with an opportunity to **meet** Research Team members you had not met before?	☐Yes **(19)**	☐No **(0)**
c)	Provide you with good **value** for your time?	☐Yes **(19)**	☐No **(0)**

Comments on the Reception & Poster Session:

- Good opportunity to see the initiatives that are underway in dementia care.
- Excellent posters! More time to review the posters would be appreciated.
- I was unaware of comprehensive research that is happening! I am contemplating pursuing PhD studies and am exploring options.
- Food was excellent. The floor plan was a little confusing, ie flow wasn't very good, some posters facing back corner.
- Consider keeping posters up around the edge of the room on Friday.
- Excellent food and good discussion.
- Well organized session. I liked the set-up for the posters, allowed people to circulate. The food is always good at the [venue], recommend this venue continue to be used.
- This is sure a great way to see how the research comes about and how studies are done.
- I was very impressed with the variety of projects. I had the opportunity to increase my knowledge about research in Saskatchewan.
- I enjoy it. Time to think about it before sessions today. Like the idea of posters staying up the next day as well.
- Could leave posters up on Friday.
- As a poster presenter it was good to come early or stay after the poster session ended to see what others were presenting.
- Leave posters up the next day a good idea.

<u>Today's Summit</u> - Please rate your satisfaction with the following *(circle appropriate response)*:

	Strongly Agree	Agree	Disagree	Strongly Disagree	Did not answer question
The time allotted for agenda items was sufficient	12	8			1
The time allotted for breaks was enough that I was able to network	12	7	1		1
Overall, I enjoyed the meeting rooms, food, and amenities of the [venue]	14	6			1
There was a good flow to the order of events during today's meeting	14	6			1
Overall, I feel like I was able to share my opinions and ideas	13	7			1
Overall, I believe that we were able to meet today's objectives	13	7			1
It was worth my time to attend the Summit today	15	4	1		1

The elements of today's summit that I liked best were:

- Research project consultation with Decision Makers.
- Had a Dr. come from the North.
- Keynote speaker: Xxx
- Violence in workplace presentation.
- Diverse crowd.
- I can't speak of anything that stands out – totally enjoyed everything.
- Variability of topics as well as the variability of people at the sessions. • Violence Awareness Programming presentation – Xxx.
- Keynote speaker – very relevant to situations in Sask.
- Variety in presentations – all very good.
- The discussion groups at the end of the day.
- Poster presentations.
- I always like the "brainstorming" sessions.
- The presentations – Xxx and Xxx.
- Small group exercise on 4 research projects.
- The food/comfort were top notch. I like the hours for start and finish.

- Sharing ideas in the afternoon.
- The flow of speakers.
- The variety of topics/speakers and audience participants.
- Feedback sessions on research proposals – very useful to me as lead.
- Excellent talks and good informal discussion.
- Great presentations and great duration.
- Enjoyed hearing about current/future projects.
- The presentation from Dr. Xxx was excellent.
- I feel Dr. Xxx and her team are doing an excellent job and I hope there is funding available to continue the Clinic.
- The groups to discuss each research project/proposal were excellent – could have been longer as it takes at least 5-10 minutes to get started and comfortable.
- The interdisciplinary participation.
- Discussion.
- The international and global collaboration and cooperation and partnerships.
- The Gentle Persuasion approach presentation was excellent and engaging.
- Presentations from good guest speakers.
- Small groups,
- Presentations on GPA – partly due to the topic and partly due to passion of the speaker.

During today's summit there were elements I did not particularly enjoy, they were:

- Nothing
- Nothing
- Nothing
- Nothing
- Would be great to have more people here – perhaps some caregivers (family caregivers).
- Involve, if possible, people with dementia – maybe not in this format but somehow to make sure they have a voice.
- Nothing
- The room was cold.
- All were informative and well presented.

If I were planning next year's summit I would make sure that:

- I continue to engage in the research consultation.
- Have a keynote speaker.
- Government involvement.
- Posters up all day on Friday.
- Work group stations.
- Same format would be good.
- Great workshop.
- All activities are in the same place as you did this year.
- Would not plan an event in the evening leading up to the Summit. Would suggest that posters and networking happen over lunch and coffee breaks.
- Nothing to add – very well organized and informative.
- I like the Thursday evening wine and food poster presentation.

- Tables rotated – people sat at different tables after each break to promote mix/mingling.
- Could the posters be left up.
- If posters were left up the next day and more time for group discussion of projects – include a few family members or individuals with dementia.
- I don't know that I would change anything.
- Political involvement.
- Open poster session and Summit to private community.
- Include provincial government.

Other comments:

- Fantastic and Rich Experience.
- Good job by all involved.
- Better to have here at [venue] compared to TCU place.
- A good mix of international info and local research.
- Annual summit face to face is excellent and I believe necessary.
- Invite Saskatchewan Health Representative to the meeting.
- I feel we are melding as a group and I am learning to understand and value research more with each summit. I consider it a privilege to be part of the Summit.
- The [venue] as a one-stop convention center is a wonderful idea.
- Continue yearly face to face meetings. Well done!
- Great event.
- Great day.
- Excellent.
- Soliciting input from everyone was great and the process of getting feedback on prepared and upcoming research projects was good.
- Poster presentation provided an opportunity to reflect and process.
- Excellent Summit – well organized.
- All sessions with all participants attending is a good format.
- Well done!

By what other means would you find useful to communicate with the team? *(check all that apply)*

teleconferences - 3
emailed newsletters/updates - 19
online community (e.g. Sharepoint; Timeless) - 3
interactive online meetings (e.g. WebEx; Illuminate) - 4
videoconferencing (e.g. Telehealth) – 4
Other suggestions:
- E-mail with weblinks
- E-mail including info that could be printed for sharing

Knowledge Network in Rural and Remote Dementia Care
3rd Annual Summit – Xxx
October 28th & 29th, 2010

EVALUATION FORM

Were you able to attend the **Reception & Poster Session** last night? ☐Yes (**20**) ☐No (**8**)

If yes, did the event:

a) Provide you with an opportunity to **learn** about research and projects that are current and relevant to rural and remote dementia care in Saskatchewan? ☐Yes (**22**) ☐No (**0**)

b) Provide you with an opportunity to **meet** Research Team members? ☐Yes (**22**) ☐No (**0**)

c) Provide you with good **value** for your time? ☐Yes (**22**) ☐No (**0**)

Comments on the Reception & Poster Session:

- I wish the people responsible for each poster would have been present at each poster and more readily available for discussion.
- Excellent posters - thank you.
- It was a lovely informal ice breaker to the following day.
- You should invite caregivers, people with dementia, health care professionals, MLA's, Ministry of Health officials, etc.
- Good opportunity to meet and speak with the different groups.
- Good atmosphere, lots of opportunities to wander and/ or to discuss posters, good length.
- I will certainly try and attend the poster session next year.
- Glad posters remained up for the second day as well.
- Might want to group posters into "theme" areas and then introduce the themes in the welcome greeting.
- Informative and interesting.
- Excellent evening but it would have made it even better if we had been introduced to one another at the beginning of the evening. The group was small enough to do this and I think would have allowed for greater interaction between the presenters.

Today's Summit - Please rate your satisfaction with the following (circle appropriate response):

	Strongly Agree	Agree	Disagree	Strongly Disagree	Did not answer question
The time allotted for agenda items was sufficient	5	17	3	1	2
The time allotted for breaks allowed me to meet other Network members	11	13	2	0	2
Overall, I enjoyed the meeting rooms, food, and am enities of the [venue]	13	12	1	0	2
There was a good flow to the order of events during today's meeting	13	13	0	0	2
Overall, I feel like I was able to share my opinions and ideas	12	14	0	0	2
Overall, I believe that we were able to meet today's objectives	11	13	0	0	4
It was worth my time to attend the Summit today	21	6	0	0	1

Comments on the above items:

- Afternoon schedule was really interesting but it may have been useful to allocate more time to the carers/ service users and also perhaps to select one research item to discuss rather than 3.
- The day is very rushed and time is very short. I believe this could be made into a longer day to 5:00.
- Great day - the representatives from the support group were fantastic and a highlight to the day.
- More time for the families would have been more helpful for us and more supportive for them.
- Not enough discussion time after panels. Excellent speakers, timely and relevant information.
- This was my first time attending. I learned so much. It was absolutely excellent. Thank you for the opportunity.
- Meeting rooms were excellent. Presentations very informative. Amenities a little short this go around for the hotel.
- When sessions go over it cuts into networking time which is crucial. Also, too much presenter time and not enough time for discussion and feedback. Please emphasize to presenters to be briefer and give more time for questions and discussion. No session had enough time for questions. Difficult to go from caregiver experiences to quick research overviews, not a good transition and I found it difficult to give attention to the projects.
- First time for me, this was great!!
- Thoroughly enjoyed the day, especially the caregiver panel. It is vitally important we engage family members more and more all the time.
- It's a great chance to join today's meeting. Hopefully will enlarge the research interests.
- I only attended one session.
- The panel discussion of the FTD caregivers telehealth support group should not have been followed by another session. It was very emotional and people needed time to "regroup". But it was an extraordinary experience and its difficult to know how they'll unfold.

The elements of today's summit that I liked best were:

- The ability to speak and make contact with other people.
- All was excellent - hard to pick one part out. Thank you.
- Xxx's presentation. Alzheimer Society's presentation.
- Panel discussion FTD Support Group.
- Rising Tide - UK NDS. Continuum of Care poster brainstorming. Xxx's research. Voices of caregivers (just think it was too much).
- Presentation from the frontal temporal dementia caregivers and Dr. Xxx.
- Xxx's presentation: A wonderful speaker. Family presentation.
- FTD caregivers messages.
- Being in a room with family members, researchers, decision makers and practitioners engaged in knowledge translation and exchange.
- Dr. Xxx's presentation and family members story sharing.
- Guest speaker from Xxx.
- Sharing of knowledge from a wide spectrum of interested professionals and caregivers.
- The personal stories - courageous, heart warming. What's happening with strategies in the UK. The research that is going on in the province.
- The guest speakers are always great.
- FTD group personal accounts. Xxx's keynote was the best I've ever attended; very interesting and appropriate for all levels of attendees.
- Interaction.
- Caregiver panel.
- Support group presentation. Xxx.
- Keynote speaker wonderful. Panel discussion FTD - WOW! Informative and moving.
- Neuropsychological tests and also the stories shared by the caregivers who gave the real experience of being with dementia patients.
- The speakers from family members.
- Excellent information, excellent presenters, good networking.
- Personal stories. Research roundtable.
- The FTD caregivers' support group discussion. Xxx's keynote was excellent and engaging.
- VERY powerful and informative panel discussion of FTD.

During today's summit there were elements I did not particularly enjoy, they were:

- I would like more time to meet people - so many great people/ research etc. to speak to.
- Xxx *[redacted comment].*
- None.
- Cold room. Quick change of groups in afternoon.
- Microphone could have been louder.
- N/ A
- No, it was all enjoyable!

If I were planning next year's summit I would make sure that:

- There was more or better representation from government and health care districts across the province.
- More government officials, more caregivers, more decision makers need to be here next year. We need health care workers here.
- It would be great if the keynote speaker had more time - he was fantastic.
- More discussion re presentations and goal setting.
- A representative from Sr. Government was present.

- Families facing dementia who have not made it to the diagnosis point involved.
- If family/ caregivers are going to be invited again to tell their stories and send their messages that (with their consent) these messages be videotaped for distribution to decision makers/ government. Very impacting and eloquent speakers.
- Research roundtable to discuss methodology and methods at end of day - formal or informal.
- I encourage others to attend.
- Information on treatment and diagnosis.
- I guess more time would be great - but I understand the financial restraints.
- More time for questions after sessions and panels. Warmer! Find a way to 'mix people up' as many sat at same tables and didn't interact or have a way to enhance people meeting each other.
- Presenters had enough time to not feel rushed or skip over slides in their presentations.
- If possible, the slides in the binders should be larger. Even with my bifocals some were too small. More time for end of day discussion group work.
- More caregivers sharing their experiences and more needs. Make the needs meet possible.
- Try to involve more First Nations participants because of the Memory Clinic focus on "remote" clients.

Other comments:

- This was such an excellent day. Perhaps people with dementia could also take part next year? Fantastic - just a huge thanks.
- If a keynote speaker like Xxx comes from England, we need to give him a much larger group than we had here today to hear what this man has to say. It is very powerful.
- Very interesting day.
- FTD panel interesting but given limited time for summit would have spent less time on it to allow broader discussion (eg. supporting young patients and/ or atypical diagnoses). As always, lots to think about for my practice and teaching.
- Well organized and great content and presenters.
- Thank you, this was a great event!
- The presentation on the memory clinic involving family members would be very useful for education purposes (video or DVD).
- Include at least one senior manager from each region, ie: VP of long term care / home care. Perhaps invite Minister of Health.
- Bathrooms - wheel chair stall did not have a raised toilet for those of us who have much difficulty with regular height toilets. Microphone on the podium not picking up the sound, difficult to hear. Good session. Thanks for the invitation.
- Great job Dr. Xxx and your team.
- Maybe instead of a draw for (or in addition to) a door prize, there could be a research "treasure hunt" of things from the poster presentations to have people better engage with the research posters. A participant commented that they found the posters intimidating. Find a way to remind of goals after lunch?
- Caregiver session very powerful. Next year I hope to make it to the evening session too. Would community pharmacists be interested as they are a source of info to the public.
- Best summit yet.
- It was great. A lot of information. Thanks!
- Thank you for the opportunity to participate in this.
- This was the first time I attended and so worth it. Thank you so much.
- Thank you for an excellent Summit. It (as usual) was very well organized and involved participants from a variety of backgrounds which made it so informative.

By what other means would you find useful to communicate with the team? (check all that apply)

☐ teleconferences **(7)**

☐ emailed newsletters/ updates **(17)**

☐ online community (e.g. Sharepoint; Timeless) **(3)**

☐ interactive online meetings (e.g. WebEx; Illuminate) **(8)**

☐ videoconferencing (e.g. Telehealth) **(13)**

EVALUATION FORM RESULTS

4th Annual Summit of the Knowledge Network in Rural and Remote Dementia Care
Xxx - October 27 & 28, 2011

30 evaluations returned out of 45 attendees

To help us better understand your responses, how would you best describe your role at the Summit?

☐ I'm a researcher in the field **(9)**

☐ I'm a student **(4)**

☐ I work directly with people with dementia in rural areas **(9)**

☐ I'm a family member of someone with dementia **(3)**

☐ I work at an administrative level in the field of dementia care **(2)**

☐ Other **(4)**

Were you able to attend the Reception & Poster Session? Yes **(24)** No **(1)** Didn't answer **(5)**

If yes, did the event:

Provide you with an opportunity to **learn** about research and projects that are current and relevant to rural and remote dementia care in Saskatchewan?	24
Provide an opportunity to interact with researchers?	24
Provide an opportunity to interact with others interested in dementia care?	24
Provide good **value** for your time?	24

Comments on the Thursday night Reception & Poster Session:

- Enjoyed this very much!
- It was nice to see that the presenters of posters were easily identifiable.

- Interesting / lots of opportunity to talk to presenters.
- Excellent resources and contacts.
- Was a good way to meet and exchange ideas.
- Enjoyed the picture of the presented attached to the poster.
- Unable to attend.
- The posters were just great.
- Excellent food.
- More structured introductions to people who were attending.
- Excellent.
- Excellent. Wonderful opportunities to discuss research with students and others.
- Still was not a full compliment of researchers standing at their posters.

Please rate your agreement with the following:

	Strongly Agree	***Agree***	***Disagree***	***Strongly Disagree***	***Did not answer question***
The time allotted for agenda items was sufficient	20	9	1		
The time allotted for breaks allowed me to meet other Network members	18	12			
Overall, I enjoyed the meeting rooms, food, and amenities of the [venue]	20	9			1
There was a good flow to the order of events during today's meeting	22	8			
Overall, I feel like I was able to share my opinions and ideas	18	11			1
Overall, I believe that we were able to meet today's objectives	17	12			1
It was worth my time to attend the Summit today	24	6			

Comments about today's meeting:

- Excellent Summit!
- Another great Summit.
- A very friendly, knowledgeable and interactive group.
- I look forward to the Summit every year.
- I enjoyed the variety of topics.
- Very well organized. Very good presentation.
- Excellent day. I always learn a lot and have lots to take back.
- I liked the keynote speech and the others. I would have like to see an empirically supported intervention to balance the anecdotally supported one.
- The session on Active Engagement was especially inspiring.
- Although there was plenty of time to meet other network members I found it challenging to do so.
- Great job!
- This is a comment to "Overall, I believe that we were able to meet today's objectives." What were they - not sure I saw specific objectives for the meeting.
- A good diversity of presenters was valuable. Great knowledge and enthusiasm.

If you stayed overnight as a guest of the Summit here in the [venue], how would you describe your room and the level of service from the hotel staff?

- Excellent
- Excellent. Comfortable bed, pleasant staff.
- Hotel staff were great. The hotel heating system could use revamping.
- Good. Rooms are not sound proof - very loud hallway noise.
- Very good.
- Top notch. My room was clean and cool. Staff courteous.
- Was great.
- Room was very comfortable. A lot of nice small touches.
- Good.

The elements of the Summit that I liked best were:

- The panel discussion.
- Opportunity to meet new people and re-connect with others who are passionate about dementia care.
- The variety and caliber of speakers. Xxx's presentation very much supported our work. Enjoyed Xxx's presentation - was her best presentation yet.
- All parts - but excellent keynote.
- The guest speaker and the presentation by Dr. Xxx - excellent. The active engagement was excellent and good timing of session.
- Guest speakers.
- Enjoyed the enthusiastic presentation from Xxx, but all presenters were interesting and informative.
- Presentations, posters, networking, group activity.
- Poster presentation.
- Good size. Not too big, not too small. Great mix of decision makers and researchers. Very relevant topics by presenters.
- The wide range of groups involved. A number of disciplines working with the Community.
- All of the presentations.
- Range of the presentations.
- Interaction with others.
- Multidisciplinary, including family members was especially important. Active engagement session.
- Getting a chance to hear from other professionals on their experiences with individuals with dementia. Also thinking about how I can use my own research to work together with individuals to improve dementia care.

- Great mix of presenters.
- Xxx.
- Diversity of the presentations. For me, it was especially interesting to hear from caregivers and the behavioural consultants. It was nice to hear talks that were different from academic research presentations.
- Results from the knowledge translation. The variety of talks and speaker backgrounds. Dr. Xxx's presentation.
- Special guest and local speakers very interesting.
- Variety of speakers - research, care consultants, volunteers, education.
- The networking, the speakers, new knowledge.
- The presentations, especially the keynote address. The diversity of presentation is really important to get all perspectives on the issues of dementia care.
- Connections among researchers, families, providers, advocates. Developing a better understanding of dementia care initiatives throughout the province.
- Complete information.

During the Summit, the things I did not enjoy were:

- Not that I did not enjoy but I would have preferred a session on GPA - Genetic Persuasive Approach and Pieces - both of which are new to me, over the actual stats of what each person does in their job.
- Enjoyed it all. Usually do!
- I enjoyed the Summit.
- Keynote presentation - very dry.
- No natural light in the main meeting room.
- Xxx *[redacted comment]*.
- Nothing
- I wish we had more opportunity to talk about future directions and how researchers and front line workers can collaborate based on the Summit discussions.
- Nothing stands out.
- Dr. Xxx
- Enjoyed whole day.
- Food was average.
- Not moving from one table, eg. where you sat at breakfast was where you stayed (until late pm).

If I were planning next year's summit I would make sure that:

- At the end of the day that there was 30 minutes set aside for individuals to be able to interact and connect with speakers. By doing this some planning on continuing an initiative could be established.
- Set the date well in advance so I can get it into my calendar.
- Have a personal connection - last year FTD SG, this year Xxx.
- Same good balance of research focused, clinical and front line stakeholders presentations - and good representation from all groups.
- We considered taking it on the road.
- CEOs, Ministry need to attend. They have control of funding.
- A multi-cultural element was introduced to inform on any facets of dementia care influenced by multi-cultural factors. Policy makers such as government officials, institution management and administrators, union officials.
- Government presence and decision makers in Regions.
- Perhaps encourage people to sit with new people after lunch.
- Keep the variety of background (eg. researchers, policy, GPs). Market it to outside SK - other provinces would benefit and be interested in the Summit.
- The Memory Clinic activities and projects are front and center, eg. even a poster explaining the key players and projects.
- All 4 Summits have been excellent. We all leave with new knowledge and re-motivated to further our work with dementias.
- There was more time for a panel discussion and get the audience more involved.
- I was invited.

Other comments:

- Great job Xxx.
- Very good to hear where we are going in research and in funding for research.
- Invite CEO's of all health regions to attend.
- Great event.
- I heard several comments about the value of the Summit - it should continue as long as it is feasible.
- Province of Saskatchewan is working on a mental health strategy - dementia could also fall in there. Is there a way to gain momentum through this angle?
- Congratulations Dr. Xxx on the prestigious award!
- Had a great time, learned a lot, look forward to next year!
- Really informative and worthwhile day.
- It also makes us aware of the big job yet to be done.
- It would have been nice to have policy makers at the Summit. Would have liked more breaks to make connections. Food was excellent.
- This was my first one, I would like to do it again.

By what other means would you find useful to communicate with the team? (check all that apply)

☐ teleconferences **(7)**

☐ emailed newsletters/ updates **(21)**

☐ online community (e.g. Timeless) **(2)**

☐ interactive online meetings (e.g. Elluminate) **(4)**

☐ videoconferencing (e.g. Telehealth, Skype) **(7)**

☐ social media (eg. Facebook) **(3)**

☐ didn't answer **(9)**

EVALUATION FORM RESULTS<br><br>**5th Annual Summit of the Knowledge Network in Rural and Remote Dementia Care**<br>**Xxx – November 15 & 16, 2012**<br><br>***31 evaluations returned out of 52 attendees***

To help us better understand your responses, how would you best describe your role at the Summit?

- ☐ I'm a researcher in the field **(6)**
- ☐ I'm a student **(4)**
- ☐ I work directly with people with dementia in rural areas **(13)**
- ☐ I'm a family member of someone with dementia **(3)**
- ☐ I work at an administrative level in the field of dementia care **(4)**
- ☐ Other **(5)**
  - Representation of Ministry of Health **(1)**
  - Alzheimer Society **(2)**

Were you able to attend the Reception & Poster Session? Yes **(27)** No **(0)** Didn't answer **(4)**

If yes, did the event:

Provide you with an opportunity to **learn** about research and projects that are current and relevant to rural and remote dementia care in Saskatchewan?	27
Provide an opportunity to interact with researchers?	27
Provide an opportunity to interact with others interested in dementia care?	27
Provide good **value** for your time?	27

Comments on the Thursday night Reception & Poster Session:

- Really enjoy this session and all the exciting poster projects.
- "Like"
- I presented a poster and found it challenging to balance presenting, with seeing other posters, and catching up with colleagues.
- As a first timer, I found it encouraging and well done.
- Good to talk with the people there and reset the brain to be ready for today.
- Well done.
- Great posters, great food.
- Enjoyed very much and found good info in the posters and discussions.
- Great to catch up with everyone. Coffee and/or tea would have been appreciated.
- Some exciting projects displayed.
- The booklet compiling all the posters is a great idea.

Please rate your agreement with the following:

	Strongly Agree	*Agree*	*Disagree*	*Strongly Disagree*	*Did not answer question*
The time allotted for agenda items was sufficient	20	11	0	0	0
The time allotted for breaks allowed me to meet other Network members	21	10	0	0	0
Overall, I enjoyed the meeting rooms, food , and amenities of the [venue]	22	9	0	0	0
There was a good flow to the order of events during today's meeting	28	3	0	0	0
Overall, I feel like I was able to share my opinions and ideas	18	13	0	0	0
Overall, I believe that we were able to meet today's objectives	24	7	0	0	0
It was worth my time to attend the Summit today	30	1	0	0	0

Comments about today's meeting:

- More time for the group activity.
- Having more time for the RaDAR focus group would have been helpful.
- Knowing how much time we had for the group project would have been good.
- Nice to see so many people working hard for a worthy cause.
- Great day.
- There is never enough time. Wish we had a bit more time for small group work.
- It would be interesting and beneficial to have more caregiver/patient input/presentation.
- Another great Summit! Thank you!

If you stayed overnight as a guest of the Summit here in the [venue], how would you describe your room and the level of service from the hotel staff?

- Very good
- Okay
- Excellent
- Lovely
- Rooms and meals were excellent.
- Very good accommodation and service.
- Very good. Staff very attentive.
- Room was great.
- Hotel staff were awesome.
- Wonderful – appreciated the newly renovated room.
- Good – all staff were pleasant.
- Good
- The room was very nice. Staff friendly and helpful.
- Room very comfortable. Inconvenient with construction and elevator out of service but understandable.
- Good. Only issue was lack of access to elevator for luggage to parking lot.

The elements of the Summit that I liked best were:

- Posters and presentations all interesting – good length, etc.
- Poster evening info (talks) on the local research (N-CAM, RaDAR).
- The links between research and practice. The variety of groups represented, having representation from the decision-makers (including the Minister) and the media – how very clever.
- The talks were excellent.
- All of the talks were really excellent. Nice diversity.
- Meeting different people.
- Afternoon session with Xxx, Xxx, and Xxx.
- The caregivers perspective.
- Story telling comments – brings humanity to research.
- Presentation on northern dementia care.
- Xxx's talk.
- FTD talk.
- CBC
- Networking, knowledge development, sharing and meeting new colleagues.
- Variety of speakers.
- Ability to network and reconnect with others.

- The video clip. “brought to life” how the NCAM works – wonderful to see where things go and see potential.
- Powerful messages from caregiver.
- Hearing from caregivers.
- Variety of topics discussed.
- Personal story.
- Xxx – excellent speaker.
- I love being in a room filled with such a diverse group of people all with a keen interest in dementia care. Wonderful networking.
- Panel discussion – FTD support group.
- Xxx – great presentation.
- All presentations were awesome – I think the Summit gets better each year.
- It was all very informative.
- Interaction
- Networking
- Caregiver presenter (Xxx)
- CBC and Xxx
- Brainstorming related to gaps and dreams (but needed more time)
- The interaction, the venue, the overall feeling of familiarity, innovation and knowledge sharing.
- Guest speaker!
- Networking with people from the rest of the community.
- Enjoyed keynote speaker – interesting information and photos and good sense of humour.
- Good to have representatives from government in attendance.
- The whole Summit was great.
- Keeping up to date on work progress and new developments.
- Networking and hearing of other’s work.
- Getting new ideas or being affirmed in being on “right track”.
- Interactive portion.
- The opportunity to share and identify gaps.
- The variety of presentation of both researchers and practitioners/caregivers.

During the Summit, the things I did not enjoy were:

- Nothing
- Talks going over a little limits networking time.
- I enjoyed the entire day.
- Cookies and coffee arrived for afternoon, but do not believe people knew of their arrival until end.
- I arrived late.
- You are doing a fantastic job, would really like to see some further opportunity with Alberta on this research. Would be willing to help facilitate. (Xxx*, Alberta*)
- Honestly, no complaints.
- No free pens ☺
- None
- Everything was great.

If I were planning next year’s summit I would make sure that:

- The poster session stays and maybe open the poster evening to other student posters for dementia outside Memory Clinic invitees.
- The talks continue to do be delivered by a range of people from different backgrounds.
- Take better notes?

- Continue to include the caregivers perspective.
- Continue with scientific session – posters – they are excellent!
- Trends/issues/policy development "how to".
- Linking research and policy – how to make the link and influence decision makers.
- Influencing the political process: - what are the steps and processes, - "how to" tool kits, etc.
- The subject matter remain fresh and relevant.
- Lots of time for caregivers and/or persons with dementia to speak.
- Maybe offer 45 minutes for lunch and a break in the pm – more time for networking.
- Ask attendees to turn off cell phones/silence them.
- More caregivers could come.
- Suggestions for communication strategies across the continuum.
- I attended. So much value in this session. Excellent work, and a great comfort to know that we are addressing gaps in our healthcare system.
- Poster presenters were not required to spend a long time beside their poster – I found it more valuable to "network" throughout the evening (after I spent sufficient time beside my poster).

Next year, a topic I would include, or special guest presenter I would invite would be:

- Xxx? A Rural GP that is competent in dementia.
- Xxx and Xxx on Age-Friendly Xxx.
- Could we address use of language we use when we speak about dementia, ie. front line staff, facility, site, care partners, care home.
- A spiritual care presenter. As dementia is fatal I would love to see someone talk about this.
- Someone developing an intervention, Telehealth Sask rep.
- Xxx to speak about respectful language in dementia care.
- Xxx – optional funding coverage.
- Family Physician – their barriers to diagnosis, etc.
- First Link Coordinators – I would like to hear about their role, and perhaps caregivers who have used First Link (although these people may be too recent to the dementia journey).

Other comments:

- Whilst developing your relationship with the media it might be worth considering media training for the team – so that they are ready to offer spontaneous comments.
- I really enjoyed the meeting and look forward to future opportunities to attend.
- Very small items: 1) Use of some language (facilitate, frontline staff) could be more person-centered or friendly. 2) Podium – difficult to see screen for most of the people in the room due to its placement.
- Utilize other opportunities for speakers such as geriatric grand rounds Thursday morning.
- Xxx *[redacted comment]*.
- As a researcher it is really important to keep Summit with a human (vs all research) element, for motivation and "nourishment" as Xxx described it. Maybe a facet of this at the poster session too?
- I'd love to see/tour remote Clinic.
- Rural and Remote Memory Clinic is unheard of in many small places. A bright, one page flyer similar to the B.U. would go a long way, especially if family questions the diagnosis of the GP.
- Very well organized and gave useful information. Lots of opportunity for input from participants.
- Enjoyed the information and networking. Very well organized.
- Great job as usual, Xxx and Xxx!

We often include up-dates about Team members' research in the pre-Summit newsletter. What else would you like to see in the newsletter?

- It's great – I like the info on other provincial AD events.
- Resources for Dementia Care in Long Term Care.

Did you receive or download a pre-summit newsletter this year?

☐ yes **(14)** If yes, do you always read the newsletter: ☐ yes **(13)** ☐ no **(3)** ☐ Did not answer **(15)**

☐ no **(2)**

☐ I do not remember **(2)**

☐ Did not answer **(13)**

Summit 6 2013

Evaluation

6th Annual Summit

The 6th Summit of the Knowledge Network in Rural and Remote Dementia Care

Xxx, October 24 & 25, 2013

Summary of 40 Returned Evaluation forms

To help us better understand your responses, how would you best describe your role at the Summit? **Note that respondents sometimes chose multiple check boxes.**

- ☐ I'm a researcher in the field **3**
- ☐ I'm a student **1**
- ☐ I work directly with people with dementia in rural areas **17**
- ☐ I'm a family member of someone with dementia **2**
- ☐ I work at an administrative level in the field of dementia care **9**
- ☐ other _**12** : ***Write-ins include: PHS, ASOS Staff X 5, was a family member/retired manager of LTC, Dementia Project Advisor, Gov't [something, can't read], Industry.***

If you attended the Thursday Evening Poster Session, did the evening: *(please skip to next section if you didn't attend)*

	Yes	No
a) Provide an opportunity to **learn** about research & projects relevant to rural and remote dementia care in Saskatchewan?	**25**	**0**
b) Provide an opportunity to **interact** with researchers?	**25**	**0**
c) Provide an opportunity to **interact** with others interested in dementia care?	**25**	**0**
d) Provide good **value** for your time?	**25**	**0**

Note: 15 people skipped this question

Comments: -love chance for networking; -handout with poster presentations very helpful; -Great evening good opportunity to network; -Lots of excellent work; -This was my first time to attend the poster presentation. Opportunities to speak with the researchers was very helpful. I truly enjoyed the networking, food & relaxed atmosphere; -Great!; -Beneficial networking time. Food was good too; -The chance to ask deeper questions is good; -Interesting research using evidence based [sic] being done; -Great posters & interesting research. Also, the food was delicious!; -Open poster night up to a larger audience; -Relaxing and informative;

Today's Summit meeting – please rate your **agreement** with the following *(circle your response)*:

The time allotted for agenda items was sufficient	Strongly Agree **24**	Agree **15**	Disagree **0**	Strongly Disagree **1**
The time allotted for breaks allowed me to meet other Network members	Strongly Agree **23**	Agree **16**	Disagree **0**	Strongly Disagree **1**
Overall, I enjoyed the meeting rooms, food, and amenities of the **[venue]**	Strongly Agree **22**	Agree **17**	Disagree **0**	Strongly Disagree **1**
There was a good flow to the order of events during today's meeting	Strongly Agree **27**	Agree **12**	Disagree **0**	Strongly Disagree **1**
Overall, I feel like I was able to share my opinions and ideas	Strongly Agree **20**	Agree **18**	Disagree **1**	Strongly Disagree **1**
Overall, I believe that we were able to meet today's objectives	Strongly Agree **23**	Agree **16**	Disagree **0**	Strongly Disagree **1**
It was worth my time to attend the Summit today	Strongly Agree **28**	Agree **11**	Disagree **0**	Strongly Disagree **1**

Comments about today's meeting:
-I was able to take so much more info back. Any more info in short time would have been too much;
-Great day;
-Round table type discussions are personally helpful;
-Very good. Glad to see the future potential;
-Excellent Day! Thank you;
-Thanks for allowing our participation;
-Thank you for letting me attend!;
-Good day!;
-[as a write-in on the scale about the room under "Agree"]: but cold, and lunch could be better;
-Chilly :) ;
-Well planned. Thank you!;
-I felt gluten-free meals were good (but not excellent) and some soup/cooked desser would have been nice;
-Very interesting to see all the research being done in this area;
-Another successful summit for Dr. Xxx's team!;
-Very good;
-Thanks so much for your work!

If you stayed overnight as a guest of the Summit here in the **[venue]**, what comments would you like to sh are
about your room and the level of service from the hotel staff?

–Staff very responsive, room very comfortable;
-Did not stay overnight;
-very good;
-Staff were excellent – professional; courteous & accommodating;
-Great! Arrived @ 8 am and my room was ready ☺ ;
-N/A;
-Room was great. Staff pleasant;
-All was fine!;
-Everything was fine, Thank You;
-Very good;
-Very good, nice team, pleasant staff;
-Great;
-Service was good, Huge lack of plug-ins
-Okay;
-fabulous!;
-Comfortable - clean - quiet

The elements of the Summit that I liked best were:

-Capacity;

-opportunity to network, great background on research being done. Being new to this group I did not feel lost or left behind in the knowledge.

-RaDAR-HQC gap analysis of rural dementia care, Dr. Xxx;

-Enthusiastic presenters and hosts, Dr. Xxx's Research;

-Plenty of opportunities to meet new people, catch up with old friends, and share information;

-Poster evening & update on grants and projects;

-Wide range of topics covered during the session and range of people from different fields. I also appreciated the warning that the room would be cold – very thoughtful;

-Capacity presentation & discussion, great info. Preliminary results from RRMC and SHQC, so great to see evidence based info;

-All the speakers brought great information. Dr. Xxx was fantastic;

-Data and how data found. Poster session and chance to talk one-on-one;

-Updates on research. Information on projects. Networking;

-Dr. Xxx;

-The group of people in the room – very diverse. Great networking;

-I appreciated that the program ran on time scheduled;

-Opportunity to listen and learn. Network with participants. I enjoyed the people! Really enjoyed Dr. Xxx's stories!:

-Poster presentation being left up for Friday, as I was unable to attend Thursday night. Networking with others & learning about programs available;

-Variety of topics covered over the day & provincial scope;

-Networking with others, hearing the updates about HQC, enviro scan, etc. Congratulations on new space!;

-Networking. I always learn so much. The research projects are so interesting. We need to move forward.

-Hearing about the research (early results). Meeting other HCP;

-Networking with other people & services

-"Bottom line" with research {cant decipher word};

-Opportunity to listen to presentation regarding research/activities. Also networking & reconnecting with others;

-Meeting people outside my field who have common interests. I always enjoy the community-relevant presentations such as last year's media presentation and this year's capacity assessment presentation;

-Dr. Xxx;

-It was all very informative and it is so exciting to see all the research being done;

-Capacity in Neurology. Enjoyed all presentations;

-Putting faces to names it is always nice to meet people in person. The afternoon presentations;

-Presentations. Enjoy the poster evening on Thursday night! Always enjoy hearing Dr. Xxx speak. The welcoming atmosphere of the RaDAR Team;

-Connecting with other professionals and becoming informed on other initiatives going on in the province;

-It was basic understanding to indepth information pieces as I am doing this thru personal interest I feel I have been brought up to speed;

-Dr. Xxx's presentation on Capacity assessment;

-Info on new or upcoming research & project progressions;

During the Summit, the things I did not enjoy were:
-There was nothing that I did not enjoy;
-Room was a bit cool at times;
-Can't think of anything I didn't enjoy;
-Ø;
-Nothing;
-Nothing;
-I enjoyed every part of Summit;
-N/A;
-Held on Friday – travel late;
-Nothing.;
-The room was cool;
-N/A;
-Temp changed quickly – but was warned;
-Coldness of room;

If I were planning next year's Summit, I would make sure that:
-All overnight guests can stay at the hotel the Summit is held at is my only complaint & I recognize this isn't always with [sic] your power;
-Grant/project updates for the team stay, poster night is more open (eg; other U of S/R students, GPs, etc.);
-Someone senior from Ministry and from U of S (one of those complaining of "no research") come;
-Lunch included warm food;
-Stories from families are included! How are employers supportive of employees who are balancing work and care;
-Introduce practical – prog & services that are in place. Putting research/prog development into practice;
-Varied presenters, from across the province – but also outside presenters who are leading the way in dementia care on a national or international scale;
-Realized that there is no representation from Xxxand some of the other RHAs;
-More Capacity. Drivers testing **{? – difficult to make out, but this is my best guess}**
-I think the collect of nursing GRNA/SALPN should be invited. When we have such a rise in dementia on the horizon we should be educating our care providers. Current programs have very little on dementia. Staff are so unprepared;
-You include time for networking;
-Quality of care in long-term care be discussed. (Dementia) What standards do we have to test the quality of care in LTC. Development of LTC standards base on the clients receiving care;
-There are takeaways for the attendees to their respective areas.

Next year, a topic I would include, or special guest presenter I would invite would be:
-Dr. Xxx again;
-Xxx;
-Would like to see follow up of the RaDAR data;
-I would like someone from Bournemouth or Scotland;
-Somone who could speak to resident violence in LTC/activities, - the role of employers to support caregivers;
-Driving & Dementia – SGI medical Re-{sp?} {?} assessment program – Xxx. They also have a progra m for Doctors that they are working on.
-More of a realistic how I can take it back to the front line. –More knowledge in stats from Home Care—looking forward to it;
-Dr. Xxx;
-Long term care – testing & development of Quality standards. How are we governing this care?;

Other comments:
-Enjoyable;
-Great work!;
-Great opportunity to also meet people and network. Renew acquaintances and make contact;
-Thank you for this opportunity. And thank you for working so hard to improve services and care for our elderly;
-Thank you for always including me, Dr. Xxx. ! Creative idea for next year to coordinate to the symposium; [Xxx]
-Great job organizing! I look forward to attending again next year;
-A great event, good presenters, lost of great info, well organized;
-Have Dr. Xxx back next year;
-Great as always;
-LOTS of good work being done – hope you share with other parts of the country!;
-Thank you for invite to Summit! Very well organized & presented. Definitely worth my time to be present;
-Another excellent day!;
-Thanks – Always enjoy this event;
-Thank you. Great Job!;
-Amazing work. I wish I was more involved from another perspective. I would have like to see the connection besides thru Alz. Society frontline work to increase quality of life as defined by each Region;
-Please do not schedule this on a Friday. 4-5 travel leaves us with no Friday eve. (Time Off);

We often include updates about Team members' research in the pre-Summit newsletter. What else would you like to see in the newsletter?
-no write-ins added by participants

Did you receive or download a pre-Summit newsletter this year?
12 skipped **22** yes **15** no **1** I do not remember
If yes:
Do you always read the newsletter? **18** Skipped **21** yes **1** no

Evaluation SUMMARY

The 7th Summit of the Knowledge Network in Rural and Remote Dementia Care

Xxx, October 21 & 22, 2014

Summary of 38 returned evaluation forms from 68 guests across two days: 56% return rate.

Note that some folk indicated multiple roles in the role description question.
Some folk skipped questions
Some folk circled across categories in the agreement response scale for Summit Meeting – I've scored as half scores.

To help us better understand your responses, how would you best describe your role at the Summit?
7 I'm a researcher in the field
3 I'm a student
17 I work directly with people with dementia in rural areas
4 I'm a family member of someone with dementia
5 I work at an administrative level in the field of dementia care
8 other: ASOS, Policy Analyst w/ Gov't, facilitate a support group, researcher in a field related to dementia.

6 This is my first Summit OR 23 I've been to a Summit before

If you attended the Tuesday Evening Poster Session, did the evening: *(please skip to next section if you didn't attend)*

	Yes	No
a) Provide an opportunity to learn about research & projects relevant to rural and remote dementia care in Saskatchewan and elsewhere?	31	0
b) Provide an opportunity to interact with researchers?	31	0
c) Provide an opportunity to interact with others interested in dementia care?	30	1
d) Provide good value for your time?	30	0

Comments about the Tuesday night Reception and Poster Session:

-Great posters! – I learned a lot about different issues in rural areas.
-This was my favorite time – I loved the different ideas and people.
-Overwhelming # of posters.
- I was unable to attend poster presentation this year. I wish there would have been an opportunity to see them the next day.
-Excellent organization and turnout[sp?]
-Very interesting, a great opportunity to meet the researchers & ask questions.
-Great opportunity for networking and seeing recent research.
-Nice to visit/ network and reconnect.
-Linear format attracted more stoppers-by than round-the-room format, and it helped that the "line" started right at the door. –Timing ~ nice to go home earlier, but increased demand for food.
-Always very interesting.
-I missed the table and chairs at the reception. These not only made eating easier, it was also a means to facilitate networking, etc.
-Could the poster booklet be emailed out before Summit so we could review them and have our questions ready!
-Very Interesting!
-Impressed by the number of posters.
-Great evening, Thanks.

		Strongly Agree	Agree	Disagree	Strongly Disagree
The time allotted for agenda items was sufficient		15	21.5	.5	
The time allotted for breaks allowed me to meet other Network members		20	17.5	.5	
Overall, I enjoyed the meeting rooms, food, and amenities of the:	[venue]	19	17	1	
	[venue]	21	15.5	.5	
There was a good flow to the order of events during today's meeting		24	13		
Overall, I feel like I was able to share my opinions and ideas		20	17		
Overall, I believe that we were able to meet today's objectives		19.5	18.5		
It was worth my time to attend the Summit today		26	12		
Combining the Summit with the International Symposium benefitted the day –not sure, didn't attend SHARP – my guess is that it was a successful endeavor. –But a bit confusing?		11	21.5	1.5	

The elements of the Summit that I liked best were:

-Care Farms. – Really enjoyed Xxx's presentation. –Interesting to see barriers are the same in different countries.

-I thought the entire Summit was a huge success! I loved Xxx's key note and other presentations. I also enjoyed taking part in the afternoon sessions prioritizing the recommendations.

-Xxx's presentations and posters -> such unique initiatives.

-Round table discussion. –Dr. Xxx.

-The Poster Session. –Presentations.

-Learning about current research (presentations) -> inspiring and great for networking.

-The afternoon discussion section. –Review of documentation policy draft.

-The small group exercise was very information and provided an opportunity to hear other perspectives. –Great work yet to come.

-Sharing ideas. –[something I can't read]

-Very well organized & kept on time. –Great variety in research projects & presentations.

-Group work provided insight into the other perspectives on dementia.

-Presentations. –Afternoon activity (brainstorming).

-Keynote and the focus group.

-Sharing of research projects – being enlightened of the great work going on.

-The prioritization discussion, and that sufficient time was allotted. –Networking.

-The opportunity to hear about other work of RaDAR group.

-I liked everything about the Summit.

-SO WELL ORGANIZED! –The opportunity to talk with a range of stakeholders interested in Rural Dementia. –Xxx's keynote speech on Wednesday am.

-The small group work ~ focused, stimulating.

-Knowledge exchange.

-I always enjoy the information exchange and the guest speaker.
-Gap Analysis and groups.
-Great speakers.
-Engaging people with dementia in technology and farm activities.
-Very interesting to hear the perspective of others – those in research & the field.
-Networking ~ seeing friends from previous sessions.
-Great networking. –Relaxed environment & open sharing. –Positive environment. –Timed session ~ begin and end of time is excellent.
-Xxx plenary discussion.
-Group discussion.
-Small group sessions.
-Small group discussion.
-Review of Draft Recommendations discussion.

During the Summit, the things I did not enjoy were:

-The cold room at breakfast.
-Nothing. –The gluten-free food was fine and available well, but a bit low on carbs.
-I did find it a little confusing by the way the presentations were listed.
-Some of the presentation were so short ~ still extremely valuable but it would be great to get more details on some of them.
-Nothing comes to mind.
-N/ A.
-The whole day was enjoyable. –I thought the program was particularly strong this year.
-0.
-The recommendation working group after being remotivated.
-Varying temps in room.
-Nothing
-The entire event was enjoyable. Speakers, sharing information, group discussions, etc.
-0.
-Little rushed.
-Interactive group activity.
-Listening/ topic re MMSE & MOCA to diagnose Alzheimer's.
- Xxx *[redacted comment]*.
-Nothing.
-Liked it all.

If I were planning next year's Summit, I would make sure that:

-Increase representation from RHAs that don't have reps -> perhaps the Alz Society can provide some connections to same.
-I found someone else to do it the following year. –That more young local physicians came.
-Keep up good work ☺.
-The variety of speakers remain diverse.
-Can't really think of anything of substance -> perhaps for airline travellers: duo tangs vs. hard binders (weight limit of luggage).
-Snacks were part of the morning coffee break.
-Continue to be so mindful of time ~ excellent.
-Follow up from notes.

-Leave it the same.
-There are always ways to do things differently but I wouldn't change the group discussions where the various stakeholders have an opportunity to share their ideas, experiences, etc.
-Consider having regional CEOs in attendance (and/or board members).
-More time for the topics ~ maybe not packing as much in if there isn't appropriate time.
-Xxx (Montessori Methods for Dementia – Ontario).
-Client/family with dementia.
-Invite some Dementia Advisory Network members as $$ allow.

Next year, a topic I would include, or special guest presenter I would invite would be:

-SGI-Physician education program re: driving & other driving assessment tools.
-I like having an international speaker – is there someone from the Philippines?
-Minister of Health (if you could persuade him).
-It would be interesting is a person diagnosed with Dementia could present.
-Dr. Xxx-A person's experience living with dementia from a rural area. Pt. stories often frame our work.
-Bring back Xxx.

The thing that stands out for me about combining the Summit and the International Symposium is:

-See the top! [referencing comments that they enjoyed the Summit in previous responses].
-Interesting poster presentations.
-Networking with people I don't usually see.
-The wide range of people I met.
-Greater exchange of information.
-The diversity of topics.
-The line between the two.
-It was nice to have a larger posters session. –It was successful to combine with SHARP.
-More poster traffic ~ break us out of old habits.
-The motivation from across the world and how ripple effect dementia has on all not just health.
-The opportunity to visit others uninvolved in previous Summits.
-Lots of opportunity to network & see what is going on.
-It seemed that there was so much on Tuesday night, and not enough time ~ again, maybe email posters out.
-How the number of issues overlap in research and information internationally.
-Increased amount of poster presentation ~ it worked well!!
-Learned from Xxx about the potential reasons for the high amount o diagnose in Scotland & Nr. Ireland -> *MAKE #s your #1 priority!
-Sharing cost and increasing awareness of related topics.
-Broader knowledge base.

Other comments:

-Thank you for inviting me!
-I really appreciated the SHARP posters.
-This is a priority to attend in my calendar. -I always go home eager to find different things to do.
-Always well organized and leave with a sense that all contribute and come away with something.
-I look forward to the Summit every year. –Great job to the organizers. –I always learn so much.

-Great job! Looking forward to next year's!
-Great Work! –Done very professionally & it was amazing to see so many intelligent & dedicated people in one room.
-Thank you! -7 years "strong" and still a well-organized, thought provoking event!
-This was my first Summit & it was a wonderful experience. It was a great forum to discuss, network & learn. I'm anxious for the next Summit!
-Have more facilitator/ speakers to speak from Cultural Group {word dpm can't read}. –It was great!
-Thanks for having this – It is much appreciated.
-A big Thank You to the organizers, speakers, and participants for a great day & exchange of ideas, etc.
-Great that Xxx reached a wider audience. –Good for increasing public awareness.
-Efficient usage of the time, very productive day. –Great job organizing several activities into a small amount of time. –Well Done!
-Great program! Thank you!

We often include updates about Team members' research in the pre-Summit newsletter. What else would you like to see in the newsletter?
____
____
____

Did you receive or download a pre-Summit newsletter this year?
22 yes 2 no 2 I do not remember
If yes:
Do you always read the newsletter? 19 yes 3 no {but look forward to rcving more}

Evaluation SUMMARY

The 8th Summit of the Knowledge Network in Rural and Remote

Summary of 49 returned evaluations from 73 Guests across two days: 67% return rate
note that some participants skipped some questions, some answered with multiple responses (on "role at Summit")

To help us better understand your responses, how would you best describe your role at the Summit?
7 I'm a researcher in the field
6 I'm a student
21 I work directly with people with dementia in rural areas
5 I'm a family member of someone with dementia
6 I work at an administrative level in the field of dementia care
10 other: ASoS (x4); researcher and program planner in another field; project on dementia; interest in dementia care; PHC; Clinical nurse educator LTC;

15 This is my first Summit OR 24 I've been to a Summit before

If you attended the **Tuesday Evening Poster Session**, did the evening: *(please skip to next section if you didn't attend)*

	Yes	No
a) Provide an opportunity to learn about research & projects relevant to rural and remote dementia care in Saskatchewan and elsewhere?	36	0
b) Provide an opportunity to interact with researchers?	35	1
c) Provide an opportunity to interact with others interested in dementia care?	36	1
d) Provide good value for your time?	36	0

Comments about the Tuesday night Reception and Poster Session:
-I look forward to this; it is so encouraging to see what areas are being reviewed
-Great to see all the fantastic research happening in SK.
-Great evening
-Really enjoyed the casual vibe and food
-There is a growing body of evidence & research that suggests that music & other arts can be a focus of treatment for dementia. Please view the documentary Alive Inside if you are interested. A poster on those alternative treatments would be great.
-Excellent posters, and food, and company!
-Excellent, well organized
-Excellent!
-Skip the voting for those attending
-Unfortunately I was not able to attend
-Sorry could not attend
-Able to meet new friends and reconnect with old ones!!
-Very well done, Thank you
-Great opportunity to network/ connect with people in the field
-Thank you to researchers who were on-site to discuss research & respond to questions
-10 is a little late to run to when we're back at 8 the next morning. Maybe end at 9 pm.
-Many good posters, difficult to choose the BEST one maybe have a first and 2nd choice?
-Really appreciated the opportunity to interact with the researchers
-I arrived last in the evening- so this was probably more so the reason why there was less interactions
-Unfortunately unable to attend – maybe alternate between Xxx and Xxx?
-Did not attend
-Seemed like fewer people were there. Lost of posters, dementia friends was great – really liked government speech

Today's Summit meeting – please rate your agreement with the following *(circle your response)*:

	Strongly Agree	Agree	Disagree	Strongly Disagree
The time allotted for agenda items was sufficient	20	27	2	0
The time allotted for breaks allowed me to meet other Network members	26	23	0	0
Overall, I enjoyed the meeting rooms, food, and am enities of the [venue]	26	22	1	0
There was a good flow to the order of events during today's meeting	32	17	0	0
Overall, I feel like I was able to share my opinions and ideas	30.5	18.5	0	0
Overall, I believe that we were able to meet today's objectives	29	18	1	0
It was worth my time to attend the Summit today	39	10	0	0
(Only if you were staying in the hotel as a guest) The guest rooms and hotel staff were welcoming	10	8	1	0

-Water on table would be great
-cold in some spots and hot in others & hard to find parking so was late getting there, most lot were full at 745 am

The elements of the Summit that I liked best were:

-Presentations from out-of-province |-Small groups |-Research evening

-I liked the On the Radar idea and should continue, this should not be last though! Have it early and allow us to interact and discuss

-Meeting others passionate about dementia care

-Dr. Xxx and networking

-Networking with others | -discussions re: care

-Panel style Q & A | -Constantly having drinks available | -Frequent breaks | -Guest presenters with different topics

-Updating of various projects that are underway or in the future for dementia care

-Keynote presentations from Ontario & Alberta

-Panel was very informative

-Keynote, panels, group discussions

-All of above | -Connecting with experts

-Panel and interactive group work

-Opportunity to hear what is happening in our province

-Panelists/ panel discussions | -Hearing about research in ON & AB

-Really enjoy the interaction of people from across the entire continuum of care

-Learning about what other provinces are doing

-Working groups

-All Summit goals were met

-Sharing & Network | -Topics of conversation

-Dr. Xxx's presentation | -Last Panel – new developments
-Poster presentations at reception | -Discussion @ tables | -Panel presentation
-PC-DATA & Panel discussion
-1 Paper-based Summit binder so I may take notes in one place and "take away" info to read, re-read & refer to in the future. | -2 Access to researchers & others interested in dementia care. | -3 PC-DATA information (core elements, ed. manual, flow sheets & algorithms).
-Small group activity – if possible I would recommend group notes from this discussion as I feel our group had great discussion which could benefit PC-DATA which was not fully captured in 4 minutes of sharing
-As always networking with others is important and appreciated. | - Speakers are always great, topics are timely.
-Attention to every detail of Summit. | -Evening and day was outstanding | -Guest speakers were good choices!
-The group discussion provided an excellent opportunity to speak to & learn from others in different health care positions & regions. | -Also meeting other individuals in various health regions
-Keynote speakers and PC-DATA | -Discussion group | -Well planned
-New/Clear[sp?] goals are provided | -Introduction of the algorithm
-Hearing about AB's plans re: dementia | -Group discussion | -Food was great as well.
-Keynote speakers were fantastic | -Breaks allowed networking
-I really enjoyed the opportunity to learn about current initiatives, research, and collaborations. | -I also liked the collaborative nature of the Summit, and the food!
-Collaborative approach to research as well as presenting
-Posters always interesting. | -Enjoy hearing about the continued work that is happening in our province
-Group break-out time
-Very well organized | -Good communication with organizers | -Kudos to Xxx
-Topics chosen | -Great job Xxx!
-Collaboration between health care providers, academia, family members, etc
-Keynote speaker this am
-It was very informative and its always exciting to see the advances being made in dementia care.
-Meet new contacts, models of service inspiration, stay in touch with best practice
-Discussion groups | -Participants were from different backgrounds/ very knowledgeable
-Panel discussion | -Brief presentations on one topic
-Interaction | -Guest Speakers | -Networking
-Overview of group discussions

During the Summit, the things I did not enjoy were:
-Everything was good
-We did not get good discussion or input from the audience at this Summit. This was a major loss.
-Not having snacks during breaks
-Overload of information. But it is dementia. That is to be expected!
-I enjoyed it all!
-I don't feel there was enough time for reporting out and discussion of the small group work ideas
-The room was cool, but I know that's out of your hands!
-Presentation in early afternoon was dense/ long
-Nothing negative comes to mind
-No concerns!
-No comments.
-Enjoyed it all
-Breaks could have been shorter
-N/A – I thought it was excellent

-No concerns
-I think there were times when some speakers felt rushed, thought I do also appreciate the need to adhere to a timely schedule.
-It was all great!
-It would be great to see more primary care providers
-N/ A
-Food wasn't as good as other years | -Geriatrician presentation could have been on just one topic – too much included
-The breaks were a little long

If I were planning next year's Summit, I would make sure that:
-We have an activity and a facilitator that encourages input/ discussion from the Summit attendees re: the small group session
-additional families present
-The poster session started and ended earlier
-No suggestions
-It is wonderful to hear both updates on ongoing RaDAR project and to learn about evolving provincial initiatives
-The main podium would be better placed at the other end of the table closer to the screen
-Shorter presentations
-Attendance by family & persons with dementia
-No comment
-More attendees (front-line service providers; eg physicians, RN, social workers & OTs) would be present from <u>all</u> health region across the province. (If in-person attendance isn't possible could technology be used to include others??)
-Everything is great that you plan
-Venue remains the same. | -Format of the Summit remains the same. | -Light snack (fruit) at afternoon coffee break.
-There is nothing I would change.
-Do you invite CEOs & VPs from the regions?
-I had more break-out sections
-15 minute coffee break | -1/ 2 hour lunch break
-"Stories" from families/ client – things that made a difference
-Different perspectives | -More regions represented
-Name tags include position as well as location
-More decision makers from each region attended

Next year, a topic I would include, or special guest presenter I would invite would be:
-Nutrition in dementia |-Look forward to hearing Dr. Xxx again
-Maybe no keynote and focus more on the On the Radar and a good small group session and discussion
-Dementia Friendly Communities
-Something relevant to dementia in Indigenous (or other cultures) persons – in particular those living in remote locations
-A government representative that might present on government plans 10-15 mins??
-More about primary health care models of dementia management seem key
-Language (e.g. "patient" "beds") and the impact on culture change
-Xxx
-Representatives from the federal government (Xxx) to update us on their recent promise of a national dementia strategy and/ or ac tion plan
- ? You seem to choose well
-Community-based organization in rural/ remote area → to hear about challenges being faced.

-Fairly new to this area & therefore unable to specify topics and/ or presenters. | -ON appears to be far ahead of SK. eg: to they have other developed models, etc that could be adapted for use in SK? Who are the experts/ leaders in ON that can present in SK?
-Presenter from Dementia Village
-Can't think of anything at the moment – Perhaps family living with dementia.
-Behaviour management tools and outcome measure
-Dementia rep from Australia to explain why they are so successful in early diagnosis and treatment of dementia
-Any!
-Alternative care methods for maintaining dementia clients at home ie: - community paramedic etc.
-Maybe a patient story or more emphasis on family caregivers
-Perhaps some personal stories from family members/ caregivers; The support they had (or not) during their journey and what else can be done to help
-Instead of having group work, could people complete the questions beforehand and the a discussion about the answers?

If you were attending Summit 9 would you prefer a paper-based Summit Binder or e-materials available for download? What other format or technology changes could we make:

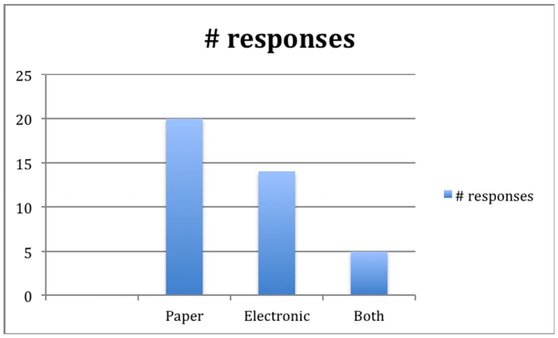

note: I've graphed out the "paper", "e", or "both" responses captured to this question. There were two "other" responses which I've marked with an ** in the response lists.

-Paper binders are good (reviewed before this session previous year)
-e-materials are fine
-paper
-paper is good, I'm not e-based yet
-Paper-based binders
-Paper is good for me
-Paper
-E-materials would be just great
-Downloads
-Paper based please, or an option for either
-E-format would be fine
-I can adapt to whatever is provided**
-Paper based please
-Paper is good
-E-materials
-I would prefer e-materials or a memory stick to use my computer
-Was recently at a conf. that did not provide printed presentations- but rather a flash drive. You could print your own material prior to conf. Potential for $ and environmental savings.**
-I like the paper based binder which lets me share info with colleague
-Prefer paper-based Summit binder, other suggestions: NA
-Paper-based –beneficial to have paper to write notes with slides
-I prefer some components of the binder to remain paper: agenda, outlines of presentations, posters, contact info. the actual slides could be available as electronic (ownCloud, SharePoint, etc).
-E-materials are great.
-Paper based is my preference
-E-material is nice
-Paper-based Binder
-Paper-based Summit Binder – it is really nice to be able to write all over it
-I enjoyed having a binder, But having e-format available would also likely be beneficial
-Summit Binder | -Actually, I would like both!
-Paper based. I would print out anyway ☺

-As long as wifi available could be ematerial
-Yes, e-materials
-e-materials, a paper based agenda (one sheet) would suffice for the day
-Paper please
-E-materials
-Paper based
-Just pdfs would be fine, maybe an agenda on the table but you put it on the screen too
-I like the binder, but would also like access online
-E-materials
-Paper based and e-materials for download

Other comments:

-We need to get more family doctors here
-One keynote (or none) |-Facilitation of discussion rather than merely reporting back from the small groups
-Great conference!
-Great job. Always a good day and very informative. We are getting there!!
-Great day!
-Fantastic!
-Great Summit & opportunities for sharing knowledge & learning from others | -interesting keynotes
-Thanks for your continued awesome work, to hear about the collaboration happening is fantastic!
-Thank you for leaving the posters up for viewing during the day
-One of my favourite days of the year! Well done.
-Excellent job | -Always enjoy the Summit!
-There is a sense of "hope"
-A great event!
-A bit of scheduled time to interact with researchers (poster session) on Wed for those who could not attend on Tuesday evening. | -Thank you for the opportunity to attend Summit 8!
-N/ A
-It would have been useful to have questions on the screen when going through that – would have been easier to follow as we did not have the other group's questions
-It was all good – Thank You
-Suggest if breaking into groups that everyone is assigned so you get a cross section of attendees. At our small group, everyone who was sitting at that table did not move so the group was not a cross representative.
-When divided into smaller groups I would suggest that there would be more formal selection of groups so groups/ people from same dept. get divided. (more rounded groups) | -I like how you are very respectful of time!

We often include updates about Team members' research in the pre-Summit newsletter. What else would you like to see in the newsletter? –
-no feedback/ suggestions at this time
-updates on research projects
-I don't think I was aware one existed
-Maybe some dementia in the news?

Did you receive or download a pre-Summit newsletter this year?
32 yes 2 no 6 I do not remember
If yes:
Do you always read the newsletter? 24 yes 6 no

Summit Evaluation Summary Report for Summit 9

Responses from all 53 attendees

Role	Researcher in field	Student	Work directly with	family member	work at admin level	other
	4.333	2.333	22.166	4.833	9	9.333

'other' desc: former research assistant, I wish to keep abreast of research
ASoS
ASoS
Retired/facilitator of a support group
ASoS
ASoS
work with people with dementia & caregivers
Educator
Support group facilitator

Tuesday Poster Session

Scale is from 1 = Excellent to 5 = Poor

	E	VG	G	F	P	X:	SD:
**Opportunity to Learn about rural/remote dementia care:**	17.0	18.0	7.0	0.0	0.0	1.76	0.73
**Opportunity to Interact with others interested in DC:**	20.0	16.0	6.0	0.0	0.0	1.67	0.72
**The Value you received in exchange for time:**	13.0	19.0	8.0	1.0	0.0	1.94	0.78
**Overall Quality of the posters/presentations:**	18.0	19.0	5.0	0.0	0.0	1.69	0.68
**Venue:**	18.0	15.0	5.0	0.0	0.0	1.66	0.71

Would you recommend the poster session to a colleague/friend: **Yes:** 40 **No:** 1

Poster Session Comments:

Many relevant, practical topics ie: Driving & Dementia, GPA
Really wonderful posters & very willing presenters to discuss their work
excellent
This was excellent, with a great selection. Gluten-free food was fine. Quality was good; people were with their posters.
Very valuable to have the author present to explain their poster!
I wasn't able to attend
N/a
Great relaxed environment to meet & speak with people doing great research.
Good opportunity to network.
Found there wasn't as much room to move & socialize as when at [venue].
Wonderful opportunity to talk directly with research, wonderful opportunity to network.
Not enough room to see Dr. Xxx & others present around the podium. Poster boards were in the way.
not enough space to see speaker because of poster set-up.
Thank you for providing the posters in a booklet. There is simply too much to absorb in a first viewing.
so relaxed and informal, great to see that side of people and the venue/staff/food was great!!!
conflicted with another evening lecture that is held annually.
Really great opportunity to meet others, this is so valuable. More space to navigate posters.
Difficult to ask questions about posters as many of the researchers were not at their posters to be available to talk about it. Also, the timing needs to change --> 9:30 is too late given a full day the next day and the early start on the 26th. Please consider making it earlier.
It was nice to meet others from different health regions.
A nice introduction to the Summit and a good opportunity to network.
I found the posters in some areas overwhelming. I learned a great deal - and the presenters were happy to share their information.
-Not all posters had someone there to answer questions. -Some posters WAY to busy to read.

Summit Meeting Impact

What has the Summit provided you that you haven't gotten elsewhere?

Exchange of "grass roots" knowledge
I appreciate that Summit is on the cusp of new research and implementation of Dementia care and

resources. - also really appreciate the small group discussions
Affirmation of the need and the high quality of work being done
all the research in one place - Networking with a variety of people - Researchers - care providers, family
networking with others of similar interests by varied backgrounds
Updates on what is happening in research & improved practices
Networking across province and stake holders. New information which can us in family meeting
Input into future research.
networking, a voice to give my perspective
Hearing & understanding what others are doing around the province & being able to network & Share
Networking with different agencies around dementia
Love the opportunity to network with people across the continuum of care.
What currently is happening in the province -> in particular programs and services that have been implemented and how they have experienced successes.
Info related to rural context and how that context influence many aspects of care.
Different perspectives from various stakeholders. -Great to meet with a group of similarly interested people in cognitive health and dementia.
Ability to connect with a wide variety of professionals that are working with and supporting people with dementia and their care providers.
Connections to people who are passionate about dementia from a broad range of perspectives.
Opportunity to connect with others face to face. Majority is SK focused.
Indepth critical thinking on dementia.
research, what is happening in the province.
-exposure to current research projects.
-Access to current local research. -Networking opportunities with researcher, professionals, and others working in rural and remote dementia care & caregiving.
-Conversations with other health care professionals & information sharing. -Excellent information from guest speakers.
Details RE: Dementia research and new initiatives.
Connections to other researchers & students.
Update on Dementia services & research in area.
Updates on various initiatives throughout the province.
Collaborative exchange between research partners, clinicians and other disciplines. Being aware that the Summit is a place to influence future research projects.
-Access to a broad range of experience in the care world. -Update on what is going on for dementia in Saskatchewan.
Sask. research data.
renewed motivation
Connection with others who are interested in dementia
More in-depth presentations & feedback on research projects
The explanation of the very good research that is happening in the country. We tend to have to work off the sides of our desks & don't have time to review what is happening across the nation.
The chance to meet with people from a variety of different professions & roles with dementia. It was interesting to hear everyone's perspectives in the group work.
-Updates on provincial initiatives. -education on new programs/research. -Networking/connecting!
Networking :)
A very multi-disciplinary gathering and that has been great getting to hear from other professional at all levels of Dementia research and care provision.
I appreciated the chance to connect with other professionals in the field.
Research specific to dementia.
Meeting colleagues and interested parties across the province.
An opportunity to meet other professionals in my field.
A increased passion for research!
An opportunity to review findings and recommend future directions for research. It also provided an excellent mix of stakeholders and experiences.
The reality of the challenges are discussed here, other conferences do not acknowledge this - they are more heavily focused on large communities.
Current research and networking opportunities with peers.
A chance to feel like I have a voice to getting some things dealt with/looked at. Appreciated the change to hear from others who are far more knowledgeable in the field than I am.
-New information on Dementia care. - Networking with researchers/other individuals with knowledge
Current state of research - some national & provincial.
Lots of information about on going Research and increased understanding of dementia care.

Overall, how do you feel the costs to your organization/department have been returned in terms of knowledge gained or shared, or connections made with others?

n/a
NA
I feel the organization gains much - not sure if they feel the same was as it isn't the easiest to measure.

Dementia care is a priority in my practice, but not in my health region.
na
Has helped in long-term care priorities and giving information to families. Has been encouraging to learn of referral resources (which I teach to other FRs)
Will share learnings with other nursing directors.
Yes! Always enjoy & find valuable. I find out what is going on in the province & share it with appropriate individuals
YES, invaluable
Yes!
I am not sure how much I will bring back to the LTC community. I do value adding the voice of LTC to discussion. Much of the content today pertained to issues that would arise prior to moving into a LTC home.
Networking possibilities are huge. Summit provide opportunity for organizations to know what other services etc. are available & the opportunity to collaborate.
more insight on other research happening in the province, and in particular how this has translated into actions (create support services)
NA --> personal benefits vs. organizational benefits
Excellent knowledge shared & gained. Great investment in time.
Well worth it!
Valuable use of times and resources - good for us to be able to make connections with/meet people who refer to the society. Awesome to learn about research/programs others are implementing
Yes - connections, information is very important.
I feel we get way more in terms of knowledge than the cost incurred. You can't put a cost on knowledge.
good use of time.
Excellent use of resources to send staff to Summit. Great opportunity to network, build relationships for future collaboration.
-very well (in returns) --> Access to current local research. -Networking. -Increased knowledge of other provincial programs (otherwise would NOT have known about).
-I'm extremely satisfied with everything I've learned.
Yes, very much - networking.
Excellent - we learn about what is happening in other regions & across Canada.
Excellent.
Always good to connect with others in dementia care but I feel there are larger picture issues in our health care systems funding and administration that we could address. Perhaps invite more ministers from government to address those issues??
The value meets the need.
I think broadening my knowledge filters out in practice and to different groups. I do discuss with my Primary Health Director/Peers.
Good value. Networking & educational opportunities no available/accessible in today's Health Care systems!
value can't be measure, the information will be passed from person to person
na
It is WELL WORTH IT! Thank you! I appreciate being able to make connections with other people.
-The connections made with others outside of SHR has been invaluable! -I will take much of today back to our entire team.
Very good return on investment. Very Pleased.
I think the knowledge gained is impactful to the service I provide, and there is great importance to connecting with others in the field.
yes.
Yes - there are not a lot of opportunities like this available.
I feel that is was worth the cost to attend as many ideas will be taken home and I was able to share my views & opinions with others.
Yes - I do feel that the time was well-spent. It provided me with a stellar example of effective knowledge translation and I had several excellent conversations about rural needs.
We cannot move forward or have impact on changes without these connections.
Valuable.
Well worth the time!
On personal time, but worth the time
Indeed - good investment of time for knowledge transferred.
Yes, it is a valuable learning tool - but I come on my own time and expense.

What changes to your work do you think you will make/have already made as a result of participating in Summit Activities or interacting with others during Summit?

n/a regarding work. The Summit does give me information that I am able to pass along or to give peers who are caregivers information about helpful organizations to which they can turn
Will try some Namaste care with elderly. Will look again the the PIECES model.
Broadening knowledge of Alzheimer's Society work and value to rural/urban SK.

Learning from others re: possible care ideas
Yes, will think of specific issues.
Dementia on community and facility agenda
Driving patterns; discussion around cessation. Namaste approach in LTC.
Presentation Re: 1st Link & Young Onset group etc. is information that I can readily use in my work with people & family living with dementia.
Interested in learning more about PIECES acronym & the 5 step adaptation approach. Namaste presentation very interesting in supporting quality of life for dementia patients. -Dr. Xxx's presentationve ry informative.
Assigning tables - mix up the attendees more.
I find the Summit very empowering and a great re-charge to go back to your work.
-Increased connections with other professionals. -Increased referrals to community services (was unaware of prior). -New ideas re: service delivery & front-line work.
-Increased Dementia awareness for health care professionals.
Business as usual mostly.
Consideration of other resource tools that may be more practical and applicable to my practice.
-Increase First Link referral. -Networking for resources to assist my practice. -How to modify current resources to fit dementia needs. -Utilize PC-DATA??
Always come away with new ideas. Poster thought provoking - always take away new info/findings that are driving thinking & practice! Relay info to health care providers I am in contact with.
na
Continue to promote/reinforce importance of completing RAI assessment accurately to ensure Data Quality as the information/data is obviously being utilized! Promote the availability of the First Link resources.
I now know about the role of RaDAR & the Alzheimers society & the ability to refer to First Link.
-Today's info will help guide future site-specific research.
Lots of research to go home and absorb further! Lots of new contacts to follow-up with.
To better be able to understand how outreach is done.
I felt that the "Minds in Motion" poster was a great idea that we would love to pilot in Xxx. The Namaste care would also be amazing - can't wait to see that research!
I will ensure that my own KT strategies are as well-designed as an event like this one. I will also be attuned to several content areas that were brought o my attentions in the discussions.
Give more direction to caregivers/family members to connect with First Link, encourage physicians to refer to the Rural and Remote Dementia Clinic.
Change assessments regarding driving. Begin to develop standard work regarding dementia screening/diagnosis within our PHC team.
-Access to new resources to use in Education Xxx {can't read the word}
1)Voluneer. 2)Share Summit info with Co-workers. 3)This has provided me a pause to reflect on some current states I often see at work and potential projects that could be developed.
Over the years I have been able to make many changes in care of our dementia residents. There is always new ideas, new programs and new knowledge.

Summit Meeting Opinion

Elements of the Summit that I liked best were:

Learning about positive steps forward coming out of the research such as use of teleheath; better clients centred care. Important to hear from front line workers also.
-small group discussions
-networking with Dementia Care and Research individuals
venue - space/air was great
networking - group discussion - ability to ask questions of presenters
groups discussion
I liked every aspect of Summit - very interesting, stimulating & exciting! Thank you.
Everything!!
meeting multiple peoples in different roles
group discussion
Networking. Group discussions.
Broad range of topics discussed
Opportunity to share ideas & collaborate with others who also.Dr. Xxx really enjoyed learning.Excellent.
group discussions and how well is was organized and laid out.
The note taking pads for participants during the group exercises were very handy. I really enjoyed the group work discussion activities.
Enjoyed the variety of the day. Great finish to the day with Dr. Xxx.
Presentations, opportunities to network
Keynote - very timely practical information, whats on the RaDAR.
Small Group Discussion

Connecting with other professionals.
Small group Discussions (I don't like standing up in large group settings). I really enjoy hearing about research projects that are occuring in SK! - as well as abroad.
-Hard to decide; overall believe all content/agenda worthwhile & relevant.
-the information presented was excellent by all speakers. - small group work was very beneficial.
Group discussion, Keynote.
Dr. Xxx - needed more time.
Dr. Xxx.
Panel. Dr. Xxx's presentation.
The discussions - the exposure to research studies.
Panel Discussion/presentation. Breakout discussion. Breakfast and lunch were good - missed fruit.
Presentation re: Driving/Dementia. Namaste care.
everything
I enjoyed all the presentations and the opportunity to have small group discussions as well. The Summit was very well organized and kept on time.
Information on the Southern 5 bed dementia assessment unit. Namaste care presentation. Dr. Xxx is a nexcellent!!! presenter - would have loved to hear more from him.
The small group discussion/sharing of experiences and ideas. I appreciated how you bring together so many people with different life & education experiences and you are willing to listen & incorporate all voices. It's refreshing!
Small group session & poster presentation.
-the opportunity for small group discussions. -excellent exchange of information & knowledge.
Having the handouts available to follow along.
Small group work.
Dr. Xxx's presentation & the Alzheimer Society were really engaging.

Group discussion related to the research.
The speakers, sharing of ideas.
group interaction, sharing of ideas/activities working in other LTC facilities.
The variety of presentation topics, the mix of participants, the openness to feedback and recommendations. Great resource/powerpoint presentation booklet as well.
The opportunity to have input in making suggestions for government support/what can be focussed on in research.
Posters. RaDAR presentation re: Xxx
Enjoyed the small group discussion. Leaner a great deal from others. Afternoon sessions - great to hear what is new & happening. Alzheimer presentation was clear and solid.
The afternoon - flow -am was good with the keynote - a lot of time was spent on the research study
Over-view of reserach projects.
Poster Presentation.
Small group session & opportunities to ask questions.
-learning and meeting with other professionals in our field.

Elements of Summit that could be improved include:

None
Needs to have a sign of the museum when coming from south
room cool
equal representation of groups. You have a great mix across the continuum of an interdisciplinary group.
Xxx *[redacted comment].*
Report out from small groups would have been good; shorten discussion time by 10-15 minutes & give more time for reporting/discussing
none that I can think of.
Nothing, a great experience.
Adding a couple of 5 minute stretching session. We sat way too long
It's Great!
?? I don't know. I think you run an excellent Summit!
Content/Agenda Excellent! Would prefer Summit to be held on a Thursday or a Friday.
I thought the summit was excellent this year.
NA
Clarity of questions in group work.
The RaDAR staff or members are not always accessible. Researcher should be highlighted a little more, not just at the poster presentation. Leader of the RaDAR team could be more engaging with new participants or people that are not a part of RaDAR.
A lot of our table discussion centred on urban issues and longterm care - I would like more rural focus. Not sure how to accomplish this.
Would appreciate copies of all PPT presentations.
Could there be equal parts research presentation to learning presentations such as more like your

cognition presentation by Dr. Xxx.
-round table set-up was awkward/uncomfortable for ~1/2 participants. -difficult sight lines.
Small group session - When the group returned, no one shared what they had discussed which was disappointing. It would have been better to have each table share a recommendation.
?
Starting the meeting with a keynote -- not really able to absorb all that was being said.
Nothing to note - well done!
The Summit has been awesome for 9 years.

Next year, a topic I would include or a special guest presenter I would invite would be:

Xxx, or a speaker from to come for 10th. Xxx, (from Calgary) president of Xxx, on end-of-life care in dementia.
Provincial resources lists by regions
Community paramedicine in LTC in SHR.
Dr. Xxx - neuroplasticity & non-pharmalogical interventions. Addressing the issue of stigma & denial in dealing with dementia.
Alzheimer Society - information on New Horizon Initiative on Dementia Friendly Communities, Warning signs campaign, and Dementia Helpline.
more focus on young onest & atypical.
1) Caregiver burnout. 2) How can we better educate family about meeting needs & responding effectively to "responsive behaviours" in the home so they may keep providing care in the home for a longer period of time?
unsure/the speakers have always been fantastic.
We need to include people with dementia.
Dr. Xxx. Pain is not often recognized in individuals with dementia.
MLAs, Ministers.
I am wondering why the only geriatrician in the province wasn't present? Or any other staff from GEMsth ey would have a lot to offer. *A longer time consideration for Dr. Xxx.
FTD --> see very challenging behaviours in LTC.
would have to think, as a first guess/suggestion the GEM physicians who have never attended Summit
Caregiver issues
Any research on Falls Prevention for Mobile Dementia Clients - Best evidence based strategies, allowing to live at risk? Would love to hear more of Dr. Xxx's topics/presentations.
I really like the presentation by Dr. Xxx.
-SCOA. -DALI-North
A caregiver or person with dementia --> OR physicians and other doctors that actually see patients also.
Dr. Xxx - he has great content on communication and understanding behaviours.
how to educate the elderly population, LTC staff, acute care staff, private care home staff, caregivers at home.
A presentation by a family caregiver or two.
gerontologist/psychiatrist.
Specific tools for early intervention.
I could have listened to Dr. Xxx all day. Perhaps he could present on language.
-patient/family story - like patient Safety CPSI -??
? Perhaps a decade of innovation - highlight results of projects/research over 10 years - where is it now? What happened as a result?

In my opinion, hosting the Summit at the [venue] was:

an excellent idea - lots of parking room, large presentation room so that small groups could work without surrounding noise.
a good change great food, roomy venue
Excellent
This was good!
a good idea. Venue worked very well. Food was great, staff excellent.
Very nice
A positive change. It encourages conversations n breaks, supports local charity, and not as sterile.
Ok - but a bit difficult to find.
good.
Great parking, handy to freeway
Great. Nice Venue.
Awesome.
Great space. Good access. Good food. Nice not to pay for parking.
Great! Good for parking, great place to walk around and food was fantastic. Also, ample space.
A nice change of location
Great venue, lots of parking.
A great move!

Parking was good, room was quite cold.
Appreciated, easy access, much better parking - nice to be able to visit Boomtown.
Neat! Enjoyed lunch time walk in Boomtown.
And excellent change, more space and place to walk on lunch break.
A very good change.
A great idea - please do this again!
The perfect location!! :)
Just fine.
Better. Room was warmer, parking was better.
Excellent.
Excellent. Much better space. Nice & Open.
Great.
Amazing - I loved it - easy to get to - convenient indoors - good flow. It only took me 8 minutes to get here, good parking.
Positive.
Fabulous!!!
An excellent idea. Room was very spacious, sound system was good. Food was excellent. However, room was too cold.
a very good venue.
A good location. I didn't know they held events. Their catering staff was excellent.
Great! Lost of parking, excellent food, somewhat uncomfortable set-up.
Wonderful - convenient location, lovely food and ability to use museum on breaks, great atmosphere and amount of space.
Fun to look around at lunch!
Wonderful. This space was great in terms of echo & room to move around. It was also nice to visit Boomtown during the lunch break.
A fitting location.
Fine.
Wonderful!
A great idea. It was a nice large space and interesting venue.
awesome!!
Excellent, good space, good food, good parking, interesting to look around. (Although, I appreciate the warning to bring a sweater!)
Awesome! Great facility -- not much interfering noise. Great parking. Great food.
Forum was great - easy to get to, easy to park - good sound system. -time to go out/fresh air if desired.
Great idea --> good parking/great food.
Good - just need to make reservations at a closer hotel.

Other Comments:

thanks for all the work of putting this excellent meeting together
Facility was great, great parking. Hotel was convenient, especially for evening event. Maybe afternoon rather than evening?
Great organization!
Thanks so much for all the organizers do to make this day a success. Also, I really appreciate how approachable the member of the RaDAR team are - much appreciated!
Another great Summit. Kudos!
Great job. It's encouraging to know there are som many educated & talented people in this fight.
I would like to see more research into the idea of Dementia Prevention much like diabetes prevention. Focus on indicators that contribute to increased risk of vascular disease - lifestyle changes, linking screening to the idea of dementia prevention - create CDMs visits in EMS re dementia prevention.
I would prefer shorter coffee breaks and to leave at 3:30 - long drive. I would prefer to have paperless format where we get a memory stick for our lap tops - Also I wish presenters had their slides available - not all where in book.
Inform re: accommodation closer to [venue]. Always enjoy Dr. Xxx's presentation - so informative even if it is just a snapshot!
Xxx was helpful and professional, an asset
I really appreciated being able to attend this Summit thank you Xxx for telling me about it. Good food as well
I would appreciate an email with Dr. Xxx's presentation.
I would leave more time for small group discussions.
Thank you for a great event!
The lunch was very plain.
Thank you very much for organizing this! :) It was grat to interact with others who provide care in rural areas and the professional who support us!
Early notice regarding ways to download documents would of helped me and I could of avoided paper.
How can we spread Best Practice in Dementia Care faster ie: Xxx - PC DATA tool customized & standard work for all RN NP & Dr. - (Primary Care Physicians). Have you presented to physician groups

- How to get 1st Link out!!! -lunch could be 45 mins
Keep up the good work - Dr. Xxx and Dr. Xxx - and too all of your team members. Great work, Thank you for allowing me to be a part of this team.
Morning coffee breaks could be shorter. Meeting room very cold. Moving to the [venue] was fine.

At today's Summit meeting . . .

	Too short	Just right	Too long
**The Time Allotted for agenda items was:**	0	51	1
**The Time Allotted for breaks was:**	1	48	2

Please rate your satisfaction with:

Scale is from 1 = Extremely Satisfied to 5 = Not at all Satisfied

	ES	VS	SS	SlS	NaaS	*X:*	SD:
**The meeting room, food, and amenities of [venue]:**	27.0	25.0	1.0	0.0	0.0	1.51	0.54
**The materials provided for the meeting:**	27.0	20.0	3.0	2.0	0.0	1.62	0.77
**Order of events during today's meeting:**	22.0	30.0	1.0	0.0	0.0	1.60	0.53
**The Small Group Session:**	28.0	20.0	5.0	0.0	0.0	1.57	0.67
**Your Opportunity to share your opinions and ideas:**	26.0	25.0	1.0	0.0	0.0	1.52	0.54
**Opportunity to increase your knowledge in r&r dementia care:**	31.0	20.0	2.0	0.0	0.0	1.45	0.57
**The Value you received for your time today:**	30.0	22.0	0.0	0.0	0.0	1.42	0.50
**Moving Summit from [venue] to [venue]:**	14.0	17.0	0.0	0.0	0.0	1.55	0.51

I would like to attend Summit next year: **Yes:** 49 **Maybe:** 1 **No:** 0

Please rate your satisfaction with:

Scale is from 1 = Extremely Satisfied to 5 = Not at all Satisfied

	ES	VS	SS	SlS	NaaS	*X:*	SD:
**The meeting room, food, and amenities of [venue]:**	10.0	7.0	1.0	0.0	0.0	1.50	0.62
**The materials provided for the meeting:**	7.0	7.0	3.0	1.0	0.0	1.89	0.90
**Order of events during today's meeting:**	5.0	12.0	1.0	0.0	0.0	1.78	0.55
**The Small Group Session:**	9.0	6.0	3.0	0.0	0.0	1.67	0.77
**Your Opportunity to share your opinions and ideas:**	6.0	11.0	1.0	0.0	0.0	1.72	0.57
**Opportunity to increase your knowledge in r&r dementia care:**	5.0	12.0	1.0	0.0	0.0	1.78	0.55
**The Value you received for your time today:**	6.0	11.0	0.0	0.0	0.0	1.65	0.49
**Moving Summit from [venue] to [venue]:**	1.0	0.0	0.0	0.0	0.0	1.00	?

I would like to attend Summit next year: **Yes:** 15 **Maybe:** 1 **No:** 0

ALL FORMS Summary 53

Who is responding?

note: some participants checked multiple boxes - decimals represent one respondent splitting across multiple responses.

**I am researcher:**	6.5	**I am student:**	0.8
**I work with PWD:**	24.3	**I am family mem:**	5.7
**I work Admin level:**	6.3	**other:**	9.3

"other" text write in:

non profit
I work in a supportive capacity in dementia care in LTC settings
I work directly with people with dementia in both rural and urban areas.
Geriatric pharmacist.
Am a trained Care Aide.
Former team member
Supper of RaDAR in SCHR
Alzheimer Society
Ministry of Health
retired, support group facilitator.
Alzheimer Society
Alzheimer Society
ASOS Staff
retired from working with people with dementia - continue to do some teaching.

Total evals returned: 53

16 from first time attendees,
35 from returning participants
2 from undeclared participants

At Monday Evening Poster Session (all responses):

	Ex	VG	G	F	P
Opportunity to learn	18	17	8	2	0
Opportunity to interact	22	11	10	0	0
Value in exch for time	21	13	7	2	0
Overall quality of posters/pres.	21	16	3	1	0
Venue	21	16	2	2	0

Would you recommend the poster session to a friend? Yes: 40 No: 0

On Tuesday Summit Meeting (all responses):

note: some participants checked multiple boxes - decimals represent one respondent splitting across multiple responses.

	Too short	Just Right	Too Long
The time allotted for agenda items was:	5.5	45	1.5
The time allotted for breaks was:	0.5	49	2.5

On Tuesday Summit Meeting (all responses):

	Ext Sat	Ver Sat	Some Sat	Slght Sat	Not Sat
Meet room/food/amenities @ [venue]	31	19	3	0	0
Materials provided for the meeting	24	24	5	0	0
Order of Events at meeting	23	29	1	0	0
The Small Group Session	27	15	5	1	2
Opportunity to share you opinion/ideas	27	21	4	1	0
Opp. to increase know. in R&R dem. care	28	22	3	0	0
Value received for your time today	28	25	0	0	0

Would you like to attend Summit next year? Yes: 50 Maybe: 2 No: 0

FIRST TIME Attendees Summary total of 16

At Monday Evening Poster Session (FIRST TIME):

	Ex	VG	G	F	P
Opportunity to learn	7	3	3	1	0
Opportunity to interact	9	1	4	0	0
Value in exch for time	8	3	3	1	0
Overall quality of posters/pres.	7	3	3	0	0
Venue	10	2	1	0	0

Would you recommend the poster session to a friend? Yes: 14 No: 0

On Tuesday Summit Meeting (FIRST TIME):

note: some participants checked multiple boxes - decimals represent one respondent splitting across multiple responses.

	Too short	Just Right	Too Long
The time allotted for agenda items was:	1.5	12.5	1
The time allotted for breaks was:	0	15	0

On Tuesday Summit Meeting (FIRST TIME):

	Ext Sat	Ver Sat	Some Sat	Slght Sat	Not Sat
Meet room/food/amenities @ [venue]	12	4	0	0	0
Materials provided for the meeting	8	6	2	0	0
Order of Events at meeting	10	6	0	0	0
The Small Group Session	9	4	2	0	1
Opportunity to share you opinion/ideas	8	6	2	0	0
Opp. to increase know. in R&R dem. care	10	4	2	0	0
Value received for your time today	10	6	0	0	0

RETURNING Attendees Summary total of 35

At Monday Evening Poster Session (RETURNING):

	Ex	VG	G	F	P
Opportunity to learn	11	12	3	1	0
Opportunity to interact	11	10	6	0	0
Value in exch for time	13	9	4	1	0
Overall quality of posters/pres.	13	12	0	1	0
Venue	10	14	0	2	0

Would you recommend the poster session to a friend? Yes: 24 No: 0

On Tuesday Summit Meeting (RETURNING): *note: some participants checked multiple boxes - decimals represent one respondent splitting across multiple responses.*

	Too short	Just Right	Too Long
The time allotted for agenda items was:	4	30.5	0.5
The time allotted for breaks was:	0.5	32	2.5

On Tuesday Summit Meeting (RETURNING):

	Ext Sat	Ver Sat	Some Sat	Slght Sat	Not Sat
Meet room/food/amenities @ [venue]	18	14	3	0	0
Materials provided for the meeting	15	17	3	0	0
Order of Events at meeting	12	22	1	0	0
The Small Group Session	17	10	3	1	1
Opportunity to share you opinion/ideas	18	14	2	1	0
Opp. to increase know. in R&R dem. care	17	17	1	0	0
Value received for your time today	17	18	0	0	0

Summit Monday Evening Poster session

Comments from: First Time Attendees

Responses to "Comments about the Monday night Reception and Poster session"

Should have introduction on Monday Afternoon - and/or before the poster presentation
All the posters were great and really informative.
Good, casual environment to read posters and chat with individuals.
Very nice to attend - great posters.
Great to see the depth and variety of research being done. (the value may be higher for first-time attendees from that standpoint).
Informative, Interactive, Social, Relaxed. Very enjoyable environment.
It would be beneficial to have some ability to increate the # of attendees - could it be extended to others who aren't necessarily attending the full day?
It was great to see poster presentations - meet some people and networking. Food was lovely.
Interactive "elevator pitch" presentations of research?
Informative, love the networking and seeing what organizations, research and people from all disciplines are providing and looking at for qualitative and evidence-based information.
Great opportunity to network and engage with others.
Great food, great posters, enjoyed the cash bar. A good opportunity to meet and chat with people.

Summit Meeting - IMPACT

Comments from: First Time Attendees

Responses to "What has Summit provided you that you haven't gotten elsewhere"

Lost of research, resources, tools for more funding.
I really enjoyed Dr. Xxx's speech and the information was very helpful.
Information directly from researchers and opportunity to speak with them afterwards.
Great to hear about the many projects going on in our province. Connections
Building relationships and networking, especially considering this is my first time attending. Insight into the variety of programs and initiatives taking place in the province.
Dementia specific!! strategies for barriers, challenges. Research and best practice specific to rural and remote! Rural LTC presentation!
Opportunity to get an overview of rural dementia care province-wide.
The information on Age-Friendly Communities.
Networking. New Ideas. Understanding research.
Networking with rural colleagues. Networking with reserachers.
An introduction to dementia community.
Fantastic interaction with Support Groups. Realizing there is support out there!! Programs and services.
So much! The networking and meeting all the professionals and disciplines, stakeholders, research towards being able to access and utilize to provide better services to my residents & clients. Leaning about all the services to access!
Networking opportunities; local, provincial research.
Networking/contact info of people associated with the many initiatives and work, research, and available resources in Saskatchewan.
It's a special opportunity to learn about local initiatives and connect with people in the province. Keeping it small is really great - makes it unique from a larger conference.

Comments from: First Time Attendees

Responses to "Overall, do you feel the costs to your org/department have been returned . . ."

It was very useful, & what I've learned from networking with various professionals
Yes. It was very useful information which can be used in the future.
Yes, great opportunity to learn from presenters re research in progress and plans for future. Great learning experience.
Yes, made lots of connections and lots of knowledge - great to attend.
Yes. Building connections with other attendees is essential (for me, at least).
Yes. Concrete exampls of immediate actions/info we can take back and use. Knowledge exchange, updates.
Yes -> increased knowledge of the multisystems.
Yes, I do. It was such a great opportunity to interact with other people working in different sections of services related to dementia and learn from them.
Very worthwhile - as this is free - only cost of hotel and vehicle - our Health Region - supportive of this learning.
Yes - will help to take back the knowledge to the team I work with to help our clients.
Yes. Has provided info re: programs available for teaching. Many opportunities for research project.
Yes! As a guest & the info provided/shared & connections is priceless.
Yes, the costs have been returned -> inspiring event that allowed for a break from my daily work that will have a positive impact on my teaching/research going forward.
Yes, I feel it will reinforce what we are doing and provide information to our rural homes on how to move forward with the culture change with respect to "The D word".

Definitely - have learned a lot over the Summit.

Comments from: First Time Attendees

Responses to "What changes in your work do you think you will make/have made already"

What I've learned and take back is the activity sensory box, so caregivers, friends, family can us to help the client.
New information to use everyday and to improve on my knowledge and to better serve clients.
Hoping for more attendance from our office.
Better recognition of barriers to recruitment for individuals in clinical fields -> being more available for clinical programs to dispel myths about the work and/or the population.
Scheduled to present/share what was learned with the team. Ideas to reinforce and add to Purposeful Interactions process.
I will take away a better understanding of the future with an emphasis on demographic trends and needs.
The interesting point about interdisciplinary work and more collaboration to go further rather than faster.
None - was fantastic!
Try to ensure the individuals whom we diagnose have the resources in their rural areas to support them on their journey.
I'm positive my research project will have been instigated/started today.
I will be creating a private/community service to Recreation Therapy in community and also providing to my residents of the LTC facility I work at re: advocating for the residents/client first and assist them and families to maintain/improve quality of life!
Consider some of the tools suggested (eg CAMCI) for my own teaching.
Provide more resources/ideas in the work place for all point of care staff.
I've met a lot of people who I would like to collaborate with and have a much better idea of many issues in the provine around dementia care.

Summit Meeting - YOUR OPINION

Comments from: First Time Attendees

Responses to "Elements of the Summit that liked best were:"

The research slides and ho it can be utilized more.
Dr. Xxx's speech.
Slides, graphs, stats with presentation.
The panels and group work.
Honestly, all of the organized parts. Discussions about research.
Networking - new research and learnings. Potential for collaborations. Rural LTC presentation!
The discussions throughout the poster presentations.
Presentations. Small discussions.
All was fantastic. First conference EVER that the technology & presentation were very smoothly presented! Great job Xxx! Fantastic food as well.
Learning about initiatives in rural areas. Small group discussion. Networking.
Small group discussion; forced the interaction between new attendees and colleagues well-known to each other and "catching-up".
Research that was being implemented.
Networking and learning from all; new initiatives.
Keynotes, research updates, poster session.
Networking and information on what's available, and research that is happening.
The small/focused atmospher, beautiful venue, I really enjoyed the small group discussions and hearing from the Alzheimer Society and everyone working directly with people with dementia & their families. Loved Xxx's talk!

Comments from: First Time Attendees

Responses to "Elements of the Summit that could be improved:"

It was well presented and so many people working passionately towards dementia care & making the communities age friendly.
All other elements were good except the group discussion took a lot of the time when we would have had more speakers. Would have preferred to have more speakers give more information. It was good to hear other's views and opinions but more topics covered would be more beneficial.
Acknowledge the treaty land @ the start. First Nation in attendence, French and new immigrants to Canada community.
Participation from leadership in LongTerm Care homes. Also, large group discussion questions were posed with differences from small group ones. Might get more involvement if we knew the questions and were given time to think about our responses (instead of having us sit in silence while we think about them). Maybe post the questions over the afternoon break, then reconvene to discuss?
More participation by decision makers, LTC caregivers(more). Expand panel presentation to concurrent sessions?
More time for speaker, maybe a two-day Summit.
Voice of individuals with dementia.
Nothing! It needs to be shared with stakeholders so they can understand what is available to prover to our clientele affected by dementia.
Including people with dementia.
Question period for each session would be nice.
It would be good to include some persons with dementia. One member of our group suggested applying for CE credits so that more physicians might attend - this would be great if possible.

Comments from: First Time Attendees

Responses to "Next year, a topic I would include, or special guest presenter I would invite:"

A stakeholder or a caregiver telling their stories. Someone who can sho the various transition that a client has to go through before they get to long term care.

First Nations speaker. Persons with dementia, more family members.
Something about the national strategy, or the roll-out/data surrounding the health region amalgamation.
Addressing challenge of balancing structure with flexibility in LTC setting to provide individualized and person-directed care.
Family physician or geriatric specialists?
Saskatchewan rural communities/dementia/transportation towards age-friendly communities.
More personal stories of exceptional care.
Topics around intellectual disability and dementia. National dementia strategy.
Changes in 1 health region and how it has affected services available. Trends and issues and how to serve the people of Sask with dementia at home (without need to go to LTC).
(1) Changes/?improvements/developments we have seen from the change from Health Regions to one Authority.
(2) Helping residents and families with the transition to LTC.

Comments from: First Time Attendees

Responses to "In my opinion, hosting the Summit at the [venue] is:"

Wonderful, what an amazing venue.
Really good. Loved the location and venue.
Great!
Nice venue - great food and friendly staff.
A great idea. Easy parking, great food, great space.
Very enjoyable venue!! (?Will it be able to accommodate concurrent session if there is ever a change to that format?)

Accessible - check! Parking was very easy too!
Good, prefer to be downtown.
Wonderful - fabulous - excellent food, location, etc.
Fine - adequate parking (free).
Lovely!
Excellent.
Good. Easy to access and close to hotels.

Convenient. I enjoyed the chance to wander through the museum.
Great!
Amazing!

Comments from: First Time Attendees

Responses to "Other Comments:"

Hopefully in the next few year the dementia clients.
Overall was really informative.
Well organized, great presenters, wonderful information.
Thanks so much, so happy to attend.
-
So glad I was able to attend :)
Thank you for the invitiation.
Thank you for the day and networking opportunities. Also appreciate the slides electronically. A follow up/update on LTC care study Dr. Xxx was doing would be great.
Really enjoyed hearing about Xxx's research also! Would be nice to hear an update in the future.

Summit Monday Evening Poster session

Comments from: Return Participants

Responses to "Comments about the Monday night Reception and Poster session"

Excellent food, great interactions, knowledgeable presenters.
Informative!++
Great venue.
It would depend. I find it difficult to understand research, but know its important in new developments.
I think a little more structure would help (ie: Intro, explain a little about who some of poster presenters are: broadly eg: U of S Students in department x, y, z, ect.)

Good representation of current research.
A great chance to interact with others.
Impressive research & great food. Would like community researchers (eg nursing MSc types).
Opportunity to learn about research initiatives, opportunity to network.
Very exciting projects. Good to see the advances being made.
Coming from a distance it's great to have opportunity to network and learn the night before and have overnight stay.
did not attend.
Excellent Opportunity to interact with others passionate about dementia care.
Suggestion: can we add a 1/2 day prior to poster session that allows us to get a "primer" of what is represented in posters and who is in the room.
Missed poster presentation evening and realize how much I missed it. There is insufficient time to view on Summit day and poster presenters are not present at posters for discussion.
I would suggest to shorten the time that presenters must stand beside their poster, from 75 to 30-45 minutes. Also, I noticed several students giving brief presentations next to their posters - repetitively - so it would be nice if they could do this once for the whole group or 1/2 the group.
It was great, a nice way to start the Summit.

Summit Meeting - IMPACT

Comments from: Return Participants

Responses to "What has Summit provided you that you haven't gotten elsewhere"

Up to date info on Dementia Research and Health Care Models
-An opportunity to be informed re current/upcoming/completed research and programs and services in SK. -Further understanding of why our health care system does not work in its current state and suggesting for improvements in rural & urban settings. How can integrated teams be activated/used in remote & rural communities (eg: my home community has no Social Workers available for support). -Access to publication "The value of aging-related research in Saskatchewan".
Excellent knowledge exhange.
-the initiative that are being created and at different phases of their development stages. So many great initiatives in our province!!
Good information about programming and research throughout the province.
Networking and being in the same room as like minded people.
Highly relevant networking. Dementia specific focus is great and unlike other conferences.
Networking!
The networking with others in the province. As well as seing what is being looked at and research directions.

Networking with others. Concrete ideas to try. Information to present to health and community people.
Witness to research, meeting with others.
A sense that we are not alone trying to develop care standards and policies.
Networking, updates on numerous projects in one place.
To know what's the trend of dementia care research, it's current impact and progress.
Renewed hope and motivation.
It is local. It is encouraging that things can be done. It is practical in having fixes of real issues.
An opportunity to meet with others who work in the same field as I do.
The interplay of research applied to daily practice.
The emphasis on dementia and rural and remote care. These are not always a priority in other events.
Connection with other folks with special interest/involvement with dementia.
The Summits are always interesting and full of information.
Focus on rural care. Great mix of topics, but all related to dementia and integrated.
Knowledge of programs that are happening around the province.
The research projects on work that's being done. Dr. Xxx's presentation on where we need to goto move fro m acute care to care in the community. Namaste toolkit.
A look into what other areas are doing.
Exposure to such a variety of people & professionals who are passionate about dementia.
Access to leading professional and researchers. Trust we are learning about most current & most evidence-based information from people directly involved.
Current research - what's been done and what is up and coming.
Discussions with a rural-specific focus. Annual, ongoing engagement with a research program and stakeholders.

Comments from: Return Participants

Responses to "Overall, do you feel the costs to your org/department have been returned . . ."

I have come on a day off because this conference is so meaningful!
Yes, the costs are returned: Increased knowledge and increased connections.

Yes, Its an opportunity to connect with those who have a similar interest in healthcare.
Yes - great information, I always learn something new when I attend.
I feel my attendance has been very worthwhile, especially in knowledge gained.
Yes I do I suppose . . . the day is good I would likely not attend the evening session. I would have a longer day to accommodate increased topics & posters.
Yes. Being updated and with a variety of professional, academic services. Networking EXTREMELY valuable as well as access to poster presentations.
Yes.
Yes. Lots of connections; feel reinvigorated.
I believe the knowledge gained by me and brought back to the workplace is unmeasurable - the connections with others in the province has assisted with a greater understanding of global issues.
Well worth every penny.
Absolutely, beneficial for contacts.
Yes, opportunity to share programming, assist with research recruitment, opportunities for partnership.
Yes. It is important to keep up to date on where we are heading or need to got with dementia care.
Yes - would recommend others to come (with proviso, have to want to be involved).
Yes
Yes.
Yes. Summit gather people who are passionate about dementia care. Conversations build enthusiasm. Information/presentations stimulate ideas --> to improve how we provide care to our patients/families/care providers.
N/A
Yes. Take away new ideas and info.
I am retired.
Yes, good cost return Excellent connections made and renewed. Another excellent keynote. Appreciate hearing about RaDAR and other research.

Yes, attending is definitely value-add for those who are reporting back.
Definitely, the networking and knowledge is worth time away from work.
Yes. Revitization[sic] in itself is worth it. Networking with those I've met at previous Summits. I always leave with a couple new ideas to share with others in my region.
Yes. Different ideas, successes/failures are important to learn from. Developing cross functional team is vital.
Yes - we make important connections with people from across the province to learn what they are doing to support people living with dementia and we can share information about how the Alz. Society can support people with dementia in their regions.
ABSOLUTELY - this Summit provides an invaluable resource to not only network and make connections but als to be inspired with new ideas and the most current info available.
N/A
Very worthwhile. A terrific learning experience.

Comments from: Return Participants

Responses to "What changes in your work do you think you will make/have made already"

I will encourage the Namaste Box for volunteers, family and during Palliative Care to east connections between caregiver and dementia client.
Possibly: -Share practical knowledge with clients (eg. Namaste boxes). -Research/Learn more re Namaste boxes. -Knowledge transfer re programs and services in SK.
-Thank you!!
learning and keeping up with dementia research is helpful when interacting with friends who have a family member or who themselves have dementia.
-Involvement in the collaborative case conferences & being part of the community circle of care & support for individuals & families affected by dementia.
I will suggest names of potential attendees from my area who may be in better position to innovate/introduce new ideas/approaches.
Increased focus on complementary therapies.
Stay on track - keep moving with research and vision of what is on the horizon for residents of SK.
New technological support.
The networking over time has allowed me to consult with confidence when there are questions in dementia care. The involvement for families as co-care providers has been reenforced as quality care - and has reenforced the quality of my care provided.
I gain a refreshed passion for what I do each year - rejuvenates me.
Already making policy changes, importance of recreation therapy.
Building up capacity to front line workers dealing with people with dementia.
Get more formal geriatric training to go along with my day to day geriatric experience.
Screening. Use of Xxx's tools.
More actively making referrals to First Link (ASOS).
N/A
Look into Interdisciplinary Toolkit (HQC)
- as above [I am retired] - Will bring some of the information to my support group.
Great reminder from Dr. Xxx about how WE [can't make out word] system fail ALC patients in hospital rather than vis-versa.
New/improved models of care. Concept of dementia friendly communities.
Better understanding of the struggles Rural health care have!
Will promote the implementation of Namaste Program. ABCD Dementia toolkit.
Think about home delivery programs differently.
Following up with contacts we've met here . . . keep "turning the gears" with the multitude of ideas I leave with and finding ways to incorporate new info.
I still try to encourage people to attend.
It's hard to know exactly at this point, but its been energizing and got the wheels turning.

Summit Meeting - YOUR OPINION

Comments from: Return Participants

Responses to "Elements of the Summit that liked best were:"

Networking, hearing about the latest research.
Keynote speaker and RaDAR team highlights. Encouraging and inspiring to hear ho Namaste being used (Past year knowledge to having it applied).
The connection of HCP.
Keynote speaker - Dr. Xxx. Panel presentations, so much exceptional work being done - very creative thin king in the rural & remote areas.
Dr. Xxx RaDAR overview and recap. *The small groups after lunch is so well done!!
Knowledge translation and networking.
Networking. Current reasearch.
Networking - and specific ideas that are useful to client care. Building relationships and maintaining relationships on a personal level.
Dr. Xxx, chatting with other attendees, Namaste presentation, age friendly communities.
Discussion session - having an opinion - from all.
Progress of previous research; meeting old and new people.
Keynote speaker was excellent and applied to all disciplies.
The people!! Well organized, great knowledge sharing. Gluten-free food was great (rolls were star!)
Key note speaker was great!
Dr. Xxx - keynote message. Networking opportunities.
Well run; timely; allowed for discussion; technology worked well.
Everything was great!
Networking. Xxx always awesome, informative.
The networking will all my old friends and learning all the exciting things happening in dementia care.
Keynote. Small group discussion.
A great mix of presentation topics. Meeting new individuals and having great discussions. Opportunity for small group discussions.
See the posters. RaDAR Team highlights.
Location. Networking. Small group discussion & feedback. The presentations were excellent. I learned lots.
Xxx - Amazing!
Opportunity to meet new people - re-connect with people across the province.
Variety of attendees. Food is always excellent.
All of it.
Variety of stakeholders in attendance. Small group discussions. Networking.

Comments from: Return Participants

Responses to "Elements of the Summit that could be improved:"

1) Slightly less small group discussion time.
2) More diversity in attendees - researchers, health care professionals, front-line workers and other people affected by dementia.
The time for panels was rushed. The lunch was not ideal. The sandwiches were not good.
More time to ask questions & discussion with panel members. -Including more community members and/or community agencies.
SHRF - I would prefer to hear more about what is happening in the ground so to speak - what was translated. More personal stories; would like ministry to make some changes.
None. Good variety and already excellent.
Expand to other communities and Universities.
More opportunities for interactive sessions. Divide small groups into a more representative cross-section.

More time to interact with others.
Shorter breaks allowing for a bit shorter day.
I do not enjoy group sessions.
Nothing.
Agree that having more people and families affected by dementia involved would be great. Physician involvement.
Find a way to include people with dementia. ?Video if logistics are too dfficult.
Can't think of anything.
None that I can think of.
Add people with dementia and more care partners to the invite list.
Need to include/have present people with dementia - even if it was in the form of video presentation/interviews. Recognize that it is complicated to bring people with dementia to the Summit. ->Importance of animals for people with dementia -> any study done on this?
(1) Maybe switch the small groups back to the morning (before people leave at lunch) and switch the panel session(s) to the afternoon.
(2) Time management seemed to be an issue which cut into time for questions. Maybe use time cards next year?
More indigenous representation.

Comments from: Return Participants

Responses to "Next year, a topic I would include, or special guest presenter I would invite:"

More case studies/personal stories of how Dementia clients and caregivers have been impacted.
Possible to have a guest presenter from UK or Australia (eg. University of Tasmania, Wicking) if not in-person via technology for presentation.
Minds in Motion. An initiative happening in a rural community that has made a different in care. First Nations & dementia.
-Person living with dementia and/or their family to discuss personal circumstances and possibly their involvement with the health regions or getting a diagnosis, community supports, etc.
Open sponsors/vendors for community programs & private services supporting people with dementia.
See responses from group work.
Presentations from wide variety of disciplines ie: different areas of healthcare (like pharmacy) or social areas/social programs to stimulate cross-discipline initiatives and research.

Neurology; changes in field of science related to dementia.
Have a person with dementia and their care provider (family) speak about their experience navigating.
Personal perspective from person with dementia or care partner.
Survey on the satisfaction of care provided in LTC from family members.
International presenter from countries who have implemented National Dementia Strategies.
Supporting dementia in homes/communities. Programs, innovations.
? Something to briefly discuss some basic science breakthroughs related to dementia.
Ambiguous loss, palliative care/end of life. Programs for Persons with Dementia.
Someone from or to speak on the National Dementia Strategy.
Education for care staff to work with people with Dementia.
Speaker on national dementia strategy/Perspectives from a person with dementia or a family caregiver.
(see above?) [comment above was "Add people with dementia and more care partners to the invite list."]
Xxx does a good job of selecting a keynote speaker.
Personal care homes (there is a list of SK Ministry website), palliative care, presenetation.
Person who has been a user of discussed services. Person with dementia.

Comments from: Return Participants

Responses to "In my opinion, hosting the Summit at the [venue] is:"

GREAT!

-good size space
-delicious food
-something extra to do during breaks.
Convenient, comfortable, affordable (eg.no parking fees) and preferred to other venues downtown.
Wonderful - the space is great for the amount of participants.
Excellent.
Great, really like the venue.
A good venue . . . lots of room to include the posters through to the next day . . . ample room for table groups . . . great food.
Well done - excellent venue.
An excellent choice.
Great!

Ok . . . nice big room, but old and drafty. More coffee please! Not enough parking.
Great venue - food and space is great.
Perfect.
Great staff, facility and food.
very convenient, opportunities to stay at more economical hotels, free parking, easy access.
Alright.
Great! A bit cold.
Excellent - good quiet venue for meeting and great to share with the changes[sp?/word?] and artefacts.
Great locations - easy to access. Free parking & great food.
Great idea! Parking is great, accessibility great, food great.
Great.
Really handy to hotel! Spacious, easy parking. Love the gift shop. :)
Good.
Ok.
Enjoyable! [Venue] is quaint and has great food & hospitality.
Great.
A good location to host the Summit.
Awesome.
Works well.
The perfect venue!
Comfortable.
Great!
Good.

Comments from: Return Participants

Responses to "Other Comments:"

Thank you for responding to my request to have the hotel closer to the venue!
More diverse population invited to Summit - more representation from Indigenous groups, people who work with newcomers and peple who work with adults with cognitive disabilities and dementia. Some urban physicians and Nurse Practitioners.
This day is very well done - organization, tone, professionalism, venue, organization. Thanks.
Food options awesome.
More structured opportunity for networking would be appreciated.
Telehealth center at rural care home/health center combinations would be fantastic (Xxx size communities).
Time management for a few speakers could be better.
Appreciate the opportunity to bring all program staff and provide updates to participants of our work!
May invite front line staff - care aid, to know their ideas and concerns affecting care they provide in LTC!
Thank you for such a great informative meeting.
Thanks to RaDAR Team! You are so welcoming and organized!!
I would like to be able to invite more front line staff next year.
Great Summit again this year. Hard to believe it's been ten years.

Thank you for an excellent event!
My hope is that one region doesn't negatively affect ongoing region[word? sp?]
A bit close schedule-wise to CAG (and midterms for us academics) so that's not necessarily a problem, but it may inform future availability to attend.

Summit Monday Evening Poster session

Comments from: Undeclared

Responses to "Comments about the Monday night Reception and Poster session"

Summit Meeting - IMPACT

Comments from: Undeclared

Responses to "What has Summit provided you that you haven't gotten elsewhere"

Contacts.
Rural focus international, national, local.

Comments from: Undeclared

Responses to "Overall, do you feel the costs to your org/department have been returned . . ."

Retired.
Yes without a doubt.

Comments from: Undeclared

Responses to "What changes in your work do you think you will make/have made already"

Sharing what services are available across the provinces.

Summit Meeting - YOUR OPINION

Comments from: Undeclared

Responses to "Elements of the Summit that liked best were:"

Contacts.
Time for networking.

Comments from: Undeclared

Responses to "Elements of the Summit that could be improved:"

Comments from: Undeclared

Responses to "Next year, a topic I would include, or special guest presenter I would invite:"

Comments from: Undeclared

Responses to "In my opinion, hosting the Summit at the [venue] is:"

Keep the local grass roots going. Federal Strategy may be all talk. Province has no money. Grass roots is where the action is.
Good.

Comments from: Undeclared

Responses to "Other Comments:"

SUMMIT 11 EVALUATION SUMMARY

Total # of Forms completed: 59

This is my first Summit: 21
I've been to a Summit before: 35

10 I'm a researcher working in the field
23 I work directy with people with dementia in rural areas
14 I work at an admin level in the field of dementia care
9 I'm a student
11 I'm a family member of someone with dementia
11 other:

"other" write-ins: ASoSx 5, Supportive Health Administration, Developer/Service Provider, Senior's Health & Continuing Care project coordinator, Research Assistant, I have dementia,

If you attended on Tuesday Poster session, rate the session:

	Excellent	Very Good	Good	Fair	Poor	SUM
Your opportunity to **learn** about rural/remote dementia care research	22	20	2	-	-	44
Your opportunity to **interact** with others interested in dementia care	25	15	2	1	-	43
The **value** you received in exchange for your time	24	17	1	1	-	43
**Overall** quality **of the posters/presentations**	26	16	2	-	-	44
Venue	20	15	5	-	-	40

Write -in on 'venue': Parking!!?

Would you recommend the poster session to a colleague/friend? 44 Yes 1 No

Comments about the Tuesday Night reception and poster session:
-So great to have this opportunity to speak with others. Great networking.
-A more informal sharing of ideas.
-Very informative and educational.
-Enjoyed touching base with folks I have met. Great food. Also appreciated Dr. Xxx's apha sia info.
-Nice opportunity to learn and network.
-I was glad there was a speaker. Food & drinks was nice.
-Nice opportunity to network and meet other professionals.
-Great posters. The short presentation by Dr. Xxx was a great addition to the evening.
-Relaxed, Enjoyable!
-Great opportunity to network!

-Opportunity to see Research and initiatives in dementia care.
-Great informal atmosphere coupled with high quality research and knowledgeable presenter s! -Welcome & presentation from Dr. Xxx very informative.
-Set up well for movement between posters. Often time too close to one another and skip because of room. Was not the case, was set up well!!
-Innovative research ideas. What is happening around the province.
-Wonderful time of networking.
-Enjoyed the presentation addition this year.
-I did enjoy/like the presentation that took place.
-Very impressive to see the work around dementia.
-This was so great to have! I appreciated being able to see all the work/research being done and to ask questions and get clarification on the work being done.
-Excellent
-I am sorry to say I missed the posters this year, but find it valuable & informative. Please continue.
-It was excellent. I was not able to make it on time – would have benefitted if I did. Dr. Xxx was excellent!
-Excellent poster sessions!
-Fabulous, but almost too brief! I enjoyed Dr. Xxx's presentation very much & think it's a good idea to include a presentation. However, maybe in addition then you need to add that much
time to keep the networking the same (ie: if ½ hour presentation, start poster session ½ hour earlier).
-For me had trouble making through them. Easier language along with important information for better understanding of model. Thank you!! Very nice.
-It was very encouraging to hear from caregivers that the poster session gave them hope and understanding that there is lots going on to improve dementia care. Their perception was that not a lot was happening.

Summit meeting Impact

What has Summit Provided you with that you haven't gotten elsewhere?
-Knowledge of what research is being done in dementia care in SK.
-Meeting other professionals, family members, caregivers in dementia
-An opportunity to meet so many health care professionals who also [sp?] and support PWD and care providers.
-Networking
-The opportunity to interact outside of SHA.
-Current & Upcoming research opportunities.
-Insight into research and what others are doing
-A chance to meet with other professional working in geriatrics.
-Support, genuine interest from other carers.
-All of the research and information about dementia as well as resources to refer to and also to connect with other professions and researcher who have the same interests in dementia care.
-A lot of resources and good information on what RaDAR is about and what it encompasses.

-Chance to interact with people from so many different disciplines/experiences of dementia.
-Directions of dementia care – where to go next.
-New energy and enthusiasm for the topic.
-Dementia from a community aspect.
-Networking with other regions.
-Better understanding of rural challenges.
-Opportunity to personally connect with people in diverse roles that I probably would not have otherwise.
-Opportunity to network with a variety of programs (Alzheimers Soc., PHC, HC, physicians, educators, and family members).
-Update on what is happening with rural dementia care.
-Opportunity to hear speakers from other countries.
-A network of people all riding the same boat.
-Focus on information relevant to LTC.
-Great opportunity to network.
-Learn evidence based strategies
-Knowledge about research being done on dementia & issues people face in rural areas.
-To meet others who specialize in dementia care in SK.
-Dementia care around the world.
-Networking with other professionals.
-Opportunity to connect with such a varied group of people who are passionate about dementia care.
-A comprehensive understanding of the issues at the forefront of providing care to people living with dementia (and their caregivers) in Saskatchewan. I appreciated that the emphasis was beyond research findings, to include meaningful group discussion and lived experiences.
-Networking with other people affected by and working with people with dementia.
-Upcoming interventions for working with individuals who are impacted by dementia.
-Interesting seeing the direction of dementia research.
-So much! Academic, personal stories, new ideas.
-Yes, opportunity to hear from a variety of health care professionals and varied experience with dementia.
-A genuine hope that people living with dementia will have a better experience/a safer experience.
-Awareness of what is being done in the province as well as other countries.
-Creating connections with researchers & others in dementia care.
-No other of its kinds.
-An overview of the research that is being done and time to network with others with the same interests.
-Getting familiar with all researchers in the field and sharing results with each other.
-Wonderful opportunity to hear international scholars and hear about new initiatives – excellent.
-A more rounded insight into how dementia affects individuals and their family.
-I really enjoyed the presentation from individuals with lived experience.
-Access to a wide range of information – research & current initiatives.

-Meeting people & conversations. Hard data from family.
-Option to connect with such a variety of individuals and professionals.
-Talking with different individuals in person.
-Researches on dementia and associated works.
-Interprofessional collaboration. Usually I work in my own silo as a discipline (nursing). This opened my eyes to all the people involved & working towards these initiatives, so I never knew of before!
-Information – research projects & future plans for research projects.
-Chance to hear about broad overview of research & clinical approaches and patient perspectives on dementia.
-Energized!! Networking.
-Yes to lived experience stories – models of care with what's going on with cognitive change.
-Information on the current research focus in this area.
-Perspectives on the research gaps from individuals in a variety of professions.
-Networking – meeting a wide variety of individuals with different experiences.
-Networking opportunity.
-Ideas to take back to my daily work.
-An opportunity to network with an interdisciplinary & multi-stakeholder group of people who are focused on dementia care within a rural context.
-The chance to explore new ways to solve challenges. People acknowledge barriers but move ahead with possibilities.
-Networking opportunities with other individuals in the field.
-Chance to learn from others & see other perspectives.
-New insights & inspirations.
-Chance to renew enthusiasm by connecting with others who are immersed in & passionate about the field.
-That so many different groups, including Alz Society care and actively tackling many issues with dementia have together & include groups from everywhere at table so to discuss & understand each persons view.
-Knowledge of what is happening in Rural Sask and what research is happening! Wow.
-The opportunity to appreciate the wealth of knowledge in our province – great for relationship & partnership opportunities.

Overall, do you feel the costs to your org/dep. Have been returned in the knowledge or connections you have gained?

-Yes
-Very much so – every year this is a great opportunity to recognize the work and forward strides that have been made in this area.
-Yes.
-Yes – I continue to make new contacts across the province & connect with potential CIs re: future research.
-Yes.
-Yes.

-I come on my own time and it is worth my time and or any cost for the knowledge and connections available.
-I gained a great deal of knowledge to my work field (home care).
-No, I think more clinical information would have been more useful for me, but it was nice to hear about some of the research & ideas coming in the future.
-Yes! Developing relationships with experts in the field.
-Yes, very much.
-Definitely – just the break out sessions alone opens up your perspective so much more than is likely working in silos as we often do.
Yes, I think that taking time to connect with the broader community is essential. It was a great overview of services/programs/experiences at different levels.
-Absolutely! I have been working in LTC for >30 years, in more than 1 role. I always learn something and share with others. I have also known Xxx for many years and was involved in one of her studies.
-Yes – usually always connect with people or resources learned about at the summit.
-It has peaked by interest in possible supports & future knowledge/research & projects that are being created in SK.
-Yes – gave me knowledge of areas of life for those with dementia that I wasn't aware of.
-Yes.
-Yes. Opportunity to learn what others are doing. I loved the keynote presentation. Many takeaways from her presentation.
-Absolutely! For the reasons stated above and below.
-Yes, It's important to stay current with dementia research and care.
-Absolutely.
-Yes. I've made a lot of professional connections.
-Yes. I plan to bring this info to my Admin table to share with others.
-Yes, raising awareness of what is out there and available is very valued in my role to share with others in LTC.
-I believe so; will help us with expanding our network in dementia care so will impact the care we can provide our clients, families, and the community as a whole.
-Absolutely.
-Yes, many times over – Idea sharing and new information to bring back to organization.
-Absolutely.
-Yes!
-Yes, it's important to be aware of what is going on in different areas in the province, and the summit.
-Yes, I have, I have learnt a lot and it was very valuable to attend Summit this year. Very beneficial.
-Yes, I get to meet these people whose names I heard a lot through e-mails. I learned a lot of new initiatives towards quality cares for people with dementia.
-Yes. This fueled my passion & was the 1st time I'd been surrounded by people all focused on the same subject matter (dementia).
-Yes. – new ideas & connections made for future research collaborations.

-Absolutely. The focus of PWD living rurally is essential to striding forward in dementia. I love hearing about ideas, interventions, models of delivery, success . . .
-I find this event energizes me in a practice that we have sad circumstance the positive is needed.
-Yes, coming together for the discussion is so important. It's a great way to know what is going on across the country.
-Yes, definitely. The knowledge gained at this meeting and constructive discussion is invaluable to moving forward in improving the lives of individuals with dementia.
-YES - a very quick day in and out of Xxx, but time very well spent in hearing about research, research in progress, service delivery, models of care, personal stories.
-YES – taking back ideas to further modify current processes to better support caregivers & individuals with dementia.
-The knowledge & connections gained/developed at the Summit well worth the day away.
-Yes.
-Hugely beneficial to me see other side. Thank you!
-Very good value to participate. Our staff look forward to attending every year. Great to meet/partner and learn from others who are passionate about dementia care.

What changes in your work do you think you will make/have already made as a result of participating in Summit activites or interacting with others during summit?

-Add to the people I have access to in order to ask questions.
-Will attempt to connect with RaDAR staff re upcoming initiative that intersect with current SHA projects.
-Working more collaboratively with other professionals.
-Evaluate caregiver education supports, cite references for those who need.
-Encouraging more patience from colleagues.
-Apply to my role as a therapist (RT) in LTC and also as my private practice on how much my link to community is needed for rural and or people that needs to/want to remain in own home to provide education to family/caregivers and co-workers/management.
-Reviewing all resources available to rural.
-Think more about early diagnosis & ensuring people being diagnosed & their caregivers get access to education and support.
-Review guidelines around fitness to drive and preparing people early in course of cognitive impairment for the challenges of not driving.
-Further investigate cognitive rehab/stages.
-Utilize some of the resources for early stage dementia for some of my LTC residents that where obviously placed in LTC due to physical limitations that are now showing signs of dementia.
-Discuss more on friendships with clients and families.
-Thinking about needs of different professionals and asking more questions based on each environment.

-I will encourage others to attend & continue to share what I have learned. If given the opportunity to participate in research in order to get funding I will support the project as Dementia Care is so prevalent – we need a plan.'
-Cognitive rehab principles into meaningful activity care planning in LTC.
-Reinforced importance of supporting people with dementia and their caregivers from diagnosis forward and the emotional impact of losing license was something I don't think I focused on enough so will try to be more aware and supportive.
-Continue to partner and advocate for early diagnosis.
-Early detection is key and to provide support throughout the dementia journey.
-Greater awareness of the experience and challenge, for people living with dementia and their caregivers. This will allow me to be more responsive in my interaction with clients. The panel highlighted for me some of the dangers in avoiding tough questions and treating patients differently. Though done with good intentions, those behaviours can be isolating for people on the receiving end.
-Increased empathy for persons with dementia and their caregivers. Increased knowledge of supports available and sharing this info with others.
-Not forgetting importance of psychosocial interventions.
-Will make me think about the patient perspective when designing research.
-I will try to arrange education session(s) in my community (eg: church) by asking speakers from afternoon sessions.
-I will ensure that we assist people with timely access to assessment/support. Even just knowing the dementia helpline phone number helps.
-Providing new information to others, help with connecting other and advancing with some ideas shared being done elsewhere to practice.
-Greater advocacy; increase knowledge of resources available.
-Reinforces the importance of a social network.
-Add RaDAR & Alzheimer Society referrals to the available options.
-Review what staff are sharing with families about diagnosis. Have we taught families about preparing for next step & progression of disease.
-Being aware of and referring to appropriate resources like Alzheimers Society. Also new ways of thinking when dealing with dementia & care providers.
-Not sure, but lots of "food for thought".
-More people come from facilities in our area.
-Mainly awareness of research, and programming in place within the province of Saskatchewan.
-Keep info in mind to discuss with clients, use in my work everyday.
-Will do more research on this subject. Actually pumped my interest in this subject.
-Educate staff – Make Alzheimers resources more visible to staff.
-Connect with Xxx about future collaborations.
-Using the GDS tool more.
-Keeping abreast of some of the RRMCi moves and hopefully partnering with people from my area in these innovations.
-I have gained valuable research ideas and have learned information and gained a deeper understanding of the disease itself.
-Importance of lived experience to research.

-Review criteria for LTC of individuals with specific dementia diagnosis.
-Dementia Friend.
-More collaboration in my research with multi-stakeholders as a result of the Summit.
-Unsure yet; other than Pots-Summit I have more people, contacts & discussions to hold as follow-up!
-Raising awareness in communities by me speaking about my experiences. Good mostly.
-I want to continue educating schools, workplaces, and care homes about "human" side of dementia.
-Need to meet with RaDAR team on an annual basis to keep each other informed of our key initiatives.

Summit meeting – your opinion

Elements of the Summit that I liked the most were:

-Keynote Speaker
-Xxx and Xxx & Xxx's experiences
-Everything!
-Networking opportunity
-Research
-Small group discussions
-Dr. Xxx
-Individual Groups
-Lived experiences.
-All of it! Research and where we can help all affected by dementia.
-For my first Summit, I liked it all, all aspects.
-The breadth of experience and knowledge of attendees – nice to meet with people covering so many aspects of dementia care.
-Live experiences panel.
-Keynote.
-Networking, new ideas/seeing what services & supports are out there.
-Personal stories – very touching – always good to remind us why we are here!
-Lived Experiences.
-Liked Keynote speaker, ideas & innovative.
-Networking, personal stories from caregivers/individuals with dementia learning about innovative rural research and providing input.
-Dr. Xxx's presentation, small group activity.
-Networking, keynote speaker, the opportunity to share ideas.
-The lived experiences presenters were wonderful, inspiring.
-Evidence based information, strategies, ideas.
-Time to meet with others, the small group discussions.
-The lived experiences presentations.
-Presentations, group discussion, networking with other professionals, Point of view across SK.
-Networking with others, hearing other peoples' experience and point of views.

-Poster session, small group discussion, panel of lived experiences.
-Everything was great – just keeps getting better! The Austrian Report, group discussions, personal stories.
-Presentations
-Networking
-The lived experience presentation, the diversity of participants.
-Interactions with many people with various experiences.
-The presentations were excellent.
-Share lived experiences were incredible.
-Lived experiences; small group discussions/Group work/working & brainstorming.
-1)Posters, 2) Keynote, 3) Lived Experiences.
-Networking, Presentations, being part of the upcoming research ideas.
-Networking.
-Caregiver presentations and Xxx's talking. Hearing about the new plans for CCNA 2.
-The lived experience presentations.
-Learning more about the work of RaDAR.
-Lived experiences panel.
-Poster presentation.
-Networking, meeting new people.
-Research projects – brain chemistry – choir & transport.
-Key note speaker was great.
-Group discussions and Dr. Xxx presentation.
-Presentations – Mainly keynote speaker.
-Real life stories – sharing.
-Poster presentations. -Discussion groups – as it forced us to mingle & talk to others & make some great connections.
-Networking, Information, personal stories.
-Broad range of presenters.
-Invigorating
-Networking, presentations, Lived Experience panel was so meaningful.
-The group discussions and personal stories.
-Hearing about dementia initiative of a different country.
-Opportunities to provide input into research priorities.
-All the research and service updates.
-The personal stories were very moving.
-Lived experience panel.
-Poster presentations.
-Lived experience & morning keynote presentations both very informative.
-Personal stories & opportunities to brainstorm priorities for research – excellent discussion.
-LIVED EXPERIENCES PANEL
-Dr. Xxx education segment in 1st evening & table discussions need little less choice or more time to discuss.
-Hearing what is happening in Saskatchewan.
-Guest speaker – very informative. Always interesting to hear about research

Next year, a topic I would include, or a special guest presenter I would invite would be:

-Xxx
-It would be great to hear about opportunities to participate in research rather than simply hearing about RaDAR's plans: I think there's lots of opportunity for partnerships and or Co-Is
-Info about education geared to first responders & front link workers such as banks, bus drivers, police, call centres.
-Recreation Therapy in community (Eastern prairies and or AB/BC as more in-depth and utilized than in Sask).
-Eden Care in Community.
-* Recreation Therapy and how to connect clients to services of leisure availability*
-Physician to speak about a dementia diagnosis.
-I would be interested in more clinical, frontline info (maybe have a half-day clinical stream to Summit?)
-Prevention/Interventions – specifics regarding physical activity, nutrition, etc. Evidence based practices.
-Barriers in care with those who have dual diagnoses – ie: schizophrenia and frontotemporal dementia.
-Legal – regarding Power of Attorney – process of documentation required for capacity assessments.
-A speaker from the Indigenous community to discuss unique needs/experiences.
-Dementia Friendly Communities.
-Palliative care decisions for people with dementia.
-Responsive behavior management education strategies.
-National dementia strategy!!
-I would recommend topic similar in nature, perhaps a more in-depth discussion of social interventions would be of interest.
-Indigenous perspective.
-Results from the Rural Transportation issue. This is extremely difficult for Rural residents.
-Risks/Resources for someone diagnosed or caring for someone with AD, dementia, etc.
-Trends & disruptions in industry or like industries that we can use to be creative in meeting community needs.
-Update on national dementia strategy.
-Cognitive rehab. What is it, evidence, who's doing it.
-Perhaps there is a community that would model dementia friendly/age friendly success – truly inclusive place in Saskatchewan would be great!!
-Xxx & deinstitutionalization of dementia care – Xxx touched on some of this.
-Topic: reducing the stigma associated with dementia.
-Addressing the issue of stigma related to dementia.
-Cognitive rehabilitation and neuroplasticity
-The national dementia strategy.
-Unsure yet – perhaps update (if available) about Bill C233/National-Level plan/strategy?
-See early onset dementia of people age 45-55 years.
-

Other Comments:

-Thanks so much for all the RaDAR Team does.
-Lots of reviews in the poster presentations, looking forward to seeing outcomes of next steps.
-Research update was great
-Would have been better with increased time, slides too small to see, yet presenter kept referencing "as you can see" . . . .
-RRMCI was a great review or where we need the areas focused and being able to come together to see where most see as top 3.
-The need for community services to all who need supports in rural settings affected by dementia needs to be continued and fill the gaps, advocate for the needs that will be an epidemic and to be prepared to serve our clients – outside – of LTC!!! Maintain @ home is essential.
-I wanted to hear more details about the rural dementia care model in Austria, more details about the day to day operations, treatment/training programs, etc.
-Presentation colour schemes make slides hard to read during presentation.
-Appreciated sticking to timelines & agenda. Lots of opportunity to network or get contact info from speakers.
-Felt deflated with the focus on community vs. LTC when this sector was well represented by participants!! I think there is a perception that LTC is well supported – and in reality there are still many without a clear diagnosis, nor individualized care plan. That said, the earlier diagnosis and work to delay move in to LTC is very worthwhile work!!
-I would have liked the Lived Experiences presenters had more time to tell their stories but I also found the researchers rushed so I realize there's just not enough time and it was a very full day.
-Well organized meeting! I appreciate the strict adherence to timelines.
-I really think it is important to get knowledge & tools into the hands of caregivers – whether they be staff in carehomes or family or private caregivers, public homecare caregivers, so interventions can occur where people with dementia live. Whenever things are presented the should be recorded and shared on a network portal.
-Great! Thanks for having me.
-Personal stories are very helpful. Sometimes too rushed. Perhaps schedule on less presentation?
-Sometimes hard to hear presenters. Need to keep mic closer or clip-on mic?
-Thank you so much for this great Summit!
-More time for the presenters. (Everyone was so worried about time during presentations)
-Increase focus on what we can go do immediately after the Summit. – how to continue to work together towards larger initiatives.
-Front line stories/what can we do?
-How are we moving forward Early diagnosis??
-Connecting with research/providers.
-No Comment

-The sound system was poor this year. I couldn't hear many of the speakers. I missed much of what the keynote speaker said.
-Sad to have missed the poster session. Thank you for the live experience speakers!
-Speaker addressing Driving intimated blame on the Xxx for cancelling STC services – I don't appreciate political commentary, while perhaps was over sensitive to this.
-Thanks for another great Summit!
-Glad temperature in room increased in afternoon.
-An informative & inspiring event, thank you for the opportunity to participate! It is great to have the opportunity to learn and network with others focused on rural dementia care.
-Another informative, well organized Summit which are a chance to review current research & talk/visit colleagues.
-I found this year to be challenging with the group discussion in the am and presentations & panel in the pm – keeping discussion to afternoon helps ensure presenters aren't "Out of Time" by end of day because the is becomes too rushed & we lose value of those presentations.
-Thank you so much for opening my eyes of work of all people involved from research – so much behind scenes stuff going on.
-Most speakers were "pressured" and "rushed" by tight timelines! Perhaps do 1.5 day of Summit to fit in everything.

Please rate your SATISFACTION with the following:

Please rate your satisfaction with the following:	Extremely Satisfied	Very Satisfied	Somewhat Satisfied	Slightly Satisfied	Not at all Satisfied	SUM
The meeting room, food, and amenities of the [venue]	31	24	2	2	-	59
The materials provided for the meeting	28	26	2	1	-	57
Morning keynote	38	18	1	-	-	57
Alzheimer Society update	18	28	8	-	-	54
Lived Experiences panel	44	11	1	-	-	56
Research highlights	22	26	6	1	-	55
On the RaDAR panel	23	22	2	-	1	48
The Small Group session	34	15	7	1	-	57
Your opportunity to share your opinions and ideas	25	23	9	-	-	57
Your opportunity to increase your knowledge in rural/remote dementia care	33	19	6	-	-	58
The value you received for your time today	39	17	2	-	-	58

Comments written on table:
-(about "research highlights"): needs more time, rushing decreases value.
-(about "research highlights"): not enough time!
-(about "research highlights"): Sped up – too quick but I will read slides later
-(about "research highlights"): not enough time given

-(about “ASoSupdate”): couldn’t always hear.
-(written in under grid): need outlets for charging devices.
-(about “. . . materials provided for the meeting”): I like paper
-(about “..opportunity to share..”): more networking time
-(about “Morning Keynote”): couldn’t hear her.
-(about “ASoSupdate”): hard to hear.
-(about “the meeting room, food ..”): squeaking noises very distracting
-(about “research highlights”): Dr. Xxx was way too rushed and there was not a clear explanation about how what she showed was going to fit into the group activity at the outset of her presentation. Had that been more clear, it may have made more sense?
-(about “On the RaDAR panel”): The Xxx presentation felt more like a “plug”/sales pitch than in informative look at the model.
-(about “Lived experiences panel”): Not enough time ☺

Who would you like to see invited to Summit Next year?

Name of Indvidiual	Name of organization
Xxx (PT)<br>Xxx(PT)<br>Xxx(EdenCare)<br>Xxx–Uof RProf.<br>Xxx –LTC-RQHR	Polytech Xxx Therapeutic Rec Program Missing r ural service gaps to home care{sp?}

Evaulation Summary - Summit 12 – Nov. 5/6 2019

Total Evaluation Forms Handed In: n = 75 (note: this is 16 more than Summit 11 2018)

This is my first Summit: 30
I've been to a Summit before: 37

Mark all that apply:

I'm a person living with dementia	2
I'm a family member of someone with dementia	12
I'm a researcher in the field	5
I work directly with people with dementia in rural areas	34
I work at an admin level in the field of dementia care	9
I'm a student	8
Other:<br>"Other" write-ins:<br>• PHC Director<br>• LTC with veterans<br>• Provide phone and educational support across province to families<br>• Interested in educating myself<br>• PT RRMC<br>• Patient Family Advisor<br>• Work with ASOS (2); ASOS staff (2); Alz. Society; Alzheimer Society	14

**If you attended the Tuesday Evening Poster Session, rate the session:**	Excellent	Very Good	Good	Fair	Poor	**SUM**
Your opportunity to **learn** about rural/remote dementia care research	36	20	4	1	0	61
Your opportunity to **interact** with others interested in dementia care	38	17	6	1	0	62
The **value** you received in exchange for your time	31	23	5	2	0	61
**Overall quality** of the posters/presentations	30	27	4	0	0	61
**Venue**	34	24	1	2	0	61
**Co-hosting the evening** with the Alzheimer Society of Saskatchewan	42	16	1	0	0	59

Comments written on the above table:

- Re: "venue": temp
- Re: "venue": lacks natural light

Would you recommend the poster session to a colleague/friend? 53 Yes; 3 No; 1 "perhaps"

Comments about the Tuesday Night Reception and Poster Session:

- Did not attend this year; have in past years – very good
- It was very informative. I would love more time to explore the posters and talk with presenters
- Liked that posters were not in rows and [were] randomly placed
- Interesting, informative
- Appreciated being introduced to the Xxx family and learning of their support of Summit. I appreciate their generosity towards RaDAR
- I appreciated the presentation by Dr. Xxx.Excellent use of time in and around networking.
- I did not attend this year and missed it!

- I think the presentation should be at 7:00 instead and then give the opportunity for people to leave or stay longer
- Enjoy informal/networking time
- Enjoyed the presentation on Tues. evening
- Value of networking
- To physically see the work happening locally is very powerful and overwhelming (in a good way!)
- An earlier start would be something to consider
- Allowed me time to pre-view some material for the next day. I was able to prioritize my time based on need for the facility I manage.
- Posters were very interesting and topics applicable to healthcare workers
- More cards/brochures that can be handed out
- Well-organized and great networking
- I liked the new set up - a lot easier to move around
- A very interesting evening
- Opportunity to discuss research poster with the actual researchers
- Really nice to meet others, faces to names, etc.
- I loved learning about the broad scope of dementia research
- It was displayed in a more welcoming room space
- Good opportunity to learn rural dementia prevalence
- Great for learning and networking
- The teamwork happening in order to tackle awareness of dementia
- Informative; great to be aware of research in SK and elsewhere
- Well done, always appreciate the wide array of posters. Consider encouraging more people to add “comment” sections on their posters so it is interactive
- Broader conceptualization about dementia prevalence
- Great posters; enjoyed getting copies as part of the package
- I liked the new physical set up of the posters (easels)
- Excellent
- Convenient location – some interesting posters that were applicable to my practice
- Wow everyone. My first Summit to see and hear the work done in Saskatchewan research
- Enjoy addition of short presentation
- Very nice set up – facilitated interactions and networking; the talk by Xxx
- Liked new (different) setup this year – room followed [flowed] better
- Great speaker

The thing that stands out for me about co-hosting the Tuesday night Reception and Poster Session with ASOS is:

- The importance of Alzheimer’s Society as a resource
- Great to have a larger audience. The speaker was fantastic.
- Time saving
- Being able to connect with the persons who created the research posters
- Learning more about the resources they provide
- There was a lot of people and good networking
- I will be back next year – best chance to talk with folks

- The various topics that are being looked into to improve quality of life for people living with dementia and their families
- I think the speaker was really good and I really enjoyed her talk
- Enjoyed Xxx's presentation
- Learning connections between the two [RaDAR & ASOS]
- The diverse ideas – nutrition! Amazing! Music – Brilliant!
- Showcases SK; great work and collaboration with other associations and groups
- Very good educational value for grassroots level
- Great networking
- The more organizations work together, the greater their strength
- Opportunity to network with RaDAR team and ASOS staff
- Everyone's explanations about their work
- Learning more about the programming the Alzheimer's Society offers
- The Dr. speaking about funding in Canada is way less than USA
- Statistical analysis of the poster presentation which helped a lot to understand dementia status
- Networking
- The opportunity to network and collaborate
- It is so packed with valuable info, just too short of time to go to all of them and ask as many questions as you hope to
- Prize event
- We have so many common stakeholders
- The number of active caregivers in attendance - - this was great – they are the experts
- Co-operation and collaboration is always positive
- The opportunities for postdoctoral fellows and others to meet and network and connect with others focusing on rural and remote dementia care
- The work of research and people's passion to collaborate
- Meeting all different groups
- Well put together
- Certainly noticed more in attendance; lots of interest by other guests
- The format of the evening

SUMMIT MEETING IMPACT

What has Summit provided you with that you haven't gotten elsewhere?

- Connection to others with common rural values and desire to improve rural services
- More information on dementia – innovative treatment, difficulties acquiring a diagnosis. And networking!
- Info about provincial research in dementia – opportunity to learn from a variety of stakeholders
- Have a chance to meet others who [are] doing research related to dementia
- Being able to coordinate with others as a larger capacity to be able to take away many realms of input and professions
- I appreciate the updates on research projects
- Increased my network of dementia researchers
- Personal testimonies of family caregivers – so powerful

- One-on-one with other people interested in the same areas of dementia research, diagnosis, and follow-up
- Networking with other providers who have information about resources you may not have access to
- Connections with others working in dementia; this Summit leaves me energized to go forward in dementia care
- Information on research about different forms of music therapy; information on nutrition and end-of-life, neglect and vascular dementia, comfort feeding
- Met new health care professionals that I have not been able to yet meet in person
- Networking; attending as a team
- Networking; Info/Resources
- Networking with a diverse group of people with a passion for dementia care; involvement in research projects; exposure to new research initiatives
- I haven't attended any other summit/conf dedicated to dementia
- Music and dance options now available (or soon?!); support for struggling caregivers – staff and family both; comfort food policy; personal stories to learn from; education for LTC home caregivers – podcasts, etc.
- Network; involvement in RRMC 2.0 – you don't know what you don't know and having those disciplines present and engaged is incredible in having input
- A sense of community and collaboration – commonality
- A concentrated amount of useful information in one day; Power of attorney info
- Information and current research findings in dementia care and especially rural dementia care
- Links to the work that is happening provincially to advance dementia education and access
- Connections/resources to help communities
- Recent research and excellent/varied presentations
- This is a one-of-a-kind event. Got so much out of it in terms of new info, ideas to take away, etc. Don't know of any other conference on rural dementia care and research
- An opportunity to connect in a multidisciplinary way with peers and people with lived experience
- Awareness of the broader rural health care context
- Knowledge about the research being done and what has been done; connecting with others involved in dementia care
- A chance to spend time with and hear about the work that is being done, similar to my practice in other areas
- The Big Picture; status today; future plans/problems; Inspiring!
- A chance to network and put faces to names
- Evidence-based data and factual concerns of the whole province can be raised and discussed by healthcare professionals and family
- Much needed information on dementia and contacts for help; information on the memory clinics
- Like-minded individuals, research posters
- Fantastic connections; seeing the scope of Saskatchewan Dementia care, research, and advocacy
- The opportunity to meet/network with people with dementia, caregivers, professionals, and researchers in the field
- An overall summary of the ongoing research in rural dementia in SK
- Ballet dance

- An introduction to the research and research initiatives being done and a chance to give input regarding same; Awareness of resources that are out there is very useful
- Ongoing work being done in Saskatchewan regarding dementia care
- How to reach out to the organization who can help people with dementia
- Information about different research projects and initiatives happening within our province
- Met people I wouldn't have otherwise; will connect with some people; learned about new initiatives I will follow up on
- A complete picture of what is happening across the province
- Face-to-face access to researchers. Thank you!
- Amazed to see the research on dementia and reaching resources to rural communities
- Opportunity for face-to-face interaction
- A chance to illustrate our work and better support our clients
- The opportunity to talk and learn from others who have such vast knowledge and experiences
- Information (knowledge based)
- A chance to share this great event with my colleagues from other Alzheimer Societies – it's hard to describe the Summit to them but by being able to have them attend – they really see the benefit of working with researchers
- Specific information about rural and remote dementia health service delivery
- An idea of the different views on care and funding from a wide variety of disciplines
- Networking opportunities
- Opportunity to talk with others who are like minded and to hear about what's being researched
- Networking and learning
- Interdisciplinary networking and conversation
- The opportunity to focus specifically on my research area of rural aging and dementia, within a supportive environment to learn and grow and a postdoctoral fellow
- The Programs and Research going on Provincially and in Canada; encourage to build the TEAM for care of caregivers and PWD. Talking to peers we have common ground; researchers have engaged PWD as part of research and unity
- Hope that the system is improving and diagnoses (timely) etc. is improving
- Opportunity to network and make connections face to face
- Variety of topics related to dementia care; great speakers and ability to meet and interact with them
- Connections with others working in the field; Updates on the ongoing research; Learning about related topics
- Rural focus
- Contacts
- New information, updates from the team and research

Overall, do you feel the costs to your organization/department [in terms of time away to participate in Summit] have been returned in the knowledge or connections you have gained? Please explain:

- Many times over
- Absolutely! It has been very informative!
- Yes. The learning opportunity and up-to-date research is worth the time and trip.

- As a student and researcher, participating here has gained me knowledge about health research in dementia
- Yes as always the knowledge and connections have been priceless
- Good value for many. Made connections with individuals I will be in touch with for work purposes.
- Yes, it's so good to be knowledgeable in recent research findings in dementia care and prevention and resources
- Yes, but I wish that some of the focus was on people living in LTC. Many LTC residents do not have a proper diagnosis and some don't develop it until they have been living there for a time.
- Yes – as above, re-energizing me in my role and expanding ideas for care in my practice is well worth it
- Yes. I have learnt more about the different ways people can be involved to support education about quality of life for persons living with dementia
- Yes
- Yes, valuable information learned that will benefit my practice
- Appreciate RaDAR support!
- Yes
- Yes. Networking – relationships continue out of Summit to consult, etc.; There's always relevant knowledge to take back i.e.: POA/Guardianship, driving, nutrition, etc.
- Yes. Many connections and valuable info i.e. POA/Guardianship, research info in the field
- Yes. Gain knowledge, info, access to resources that have potential to enhance quality of life for caregivers and residents
- Absolutely! Xxx's whole presentation! The dancing innovation-I'm feeling inspired to do more!
- It is worth the cost, yes! It gives a chance to take back knowledge which generates new conversation and debate with colleagues
- Absolutely. Especially the RD's info on EoL feeding; Memory clinic; Dance info by Dr. Xxx; some of the info I can immediately utilize in my role
- Yes, in knowledge in dementia care, collaboration with others interested in dementia research care
- Yes – every year there are multiple nuggets of information that are directly attributed to work
- Yes
- n/a
- Yes. Knowledge gained will help inform decisions/practice going forward
- Yes! The costs I put in in terms of prep time was worthwhile for the exposure I got to other research
- Yes because it's a good place to learn about the resources available in my community or other communities
- Yes, expenses were minimal to attend
- Very informative; researchers help a lot in gathering data to support findings with dementia information
- Yes, even to my senior peers in my community and the PA patient advisory groups
- Totally. Opportunity to build relationships, make professional connects to other people in other areas of the province
- Yes. I've gained a lot of knowledge and insight that will guide my research
- Yes as I have gained further knowledge in how to meet the needs of the clients I serve

- Yes, by understanding the process the research is necessary to move forward; the pamphlets on each poster was informative
- Yes – being new to the RaDAR program I feel I have gained more understanding of the program and where it is going in the future
- Yes!
- Yes, the poster presentation was very helpful. I also enjoyed the “neglect” presentation which explained what happens with people with dementia
- Yes. I learned a lot and made a lot of connections that will help me in my work
- Yes. Same comments as above [met/connect with new people and initiatives to follow up]
- Yes – it would be impossible to give the same information in an agency training
- Yes, important to: 1. Be aware of research/research results!! 2. Connect with other professionals and people affect by/living with dementia
- Yes, presentations and upcoming projects and hearing the living experiences
- The value of the opportunity for connections would be difficult to cost
- Yes – this will improve assessments
- YES. A resounding **yes**, absolutely.
- Yes, learn a lot about prevention and quality of life care of dementia people
- Yes – very beneficial to have our staff attend
- Very thought-provoking - - opportunity to consider how Alberta can learn from the Saskatchewan experience
- Yes – we were trying to promote our research and get feedback and that was successful
- Very much, always able to take back information to those who I support in my day to day work
- Yes
- Immense value in order to go forth being more aware of dementia
- Yes
- Connections of people throughout Canada. Sask. teams and people with great visions and research knowledge and experience and sharing and collaboration of RaDAR pilot projects and funding and increase staff as these numbers grow
- Yes very much so - - all our staff say this is a highlight of professional development. We appreciate the opportunity to bring all our staff
- I feel that the value of me attending the Summit was worth the costs (networking, acquisition of new knowledge)
- Yes. Expansion of knowledge of ASC [ASOS] resources
- Yes
- Yes

What changes in your work do you think you will make/have already made as a result of participating in Summit activities or interacting with others during Summit? (please share an example if you wish)

- Continue to support additional sites and current sites
- Advocating more for my residents and for more activities on their floor to increase QoL
- Incorporate new learning into my teaching; thinking differently about advocacy for my profession and their role in dementia care.
- I am working on the research among aging populations. I have not focused only on dementia but also other chronic conditions

- Alzheimers link to families/staff, providing choices of programs to residents re: ballet dance exercises to utilize; use as education with staff or colleagues in recreation therapy; provide more services available to rural/community members through my recreation therapy
- Refer to more recent research to inform policy. Invite collaboration of others.
- We would like to incorporate the dance exercise sessions in our care home
- Bringing ideas into our local nursing home that I heard about while networking and lunching with strangers
- I will access the Alzheimers Society more often
- Great ideas on how to deliver care and this year, a clear path to how I can access other disciplines to improve care in my memory care clinic
- Share more about RRMC and the amazing work being done; more confidence sharing information on power of attorneys
- I don't think much of my work would change besides the knowledge I gained through the updates given throughout the day
- Involving Alzheimers Society a lot more
- Incorporating arts/dance into programs
- Access to services
- Driving – connected with SGI – presentation made to our team by SGI; POA/Guardianship – always appreciated to gather more info
- Will request a Comfort Feeding policy; will look to enhance over chair exercise program; sharing Dance Seniors; will share contact info with a particular person who would both benefit and be an asset from/to RaDAR
- Sign up for Music Dance access as soon as available; find and use a comfort food policy; find and use caregiver education; participate in research as opportunities present
- Having more contacts – being able to personally reach out to those experts; ideas for improvements – example, dancing, knowing what's happening, and where I can reach out to help programs
- Policy discussions and changes
- End of life feeding; dance integration for LTC residents; more attention to family members once residents have been admitted
- RaDAR – dementia clinics; follow-up with clinic clients
- Advocate for practitioner education in regards to domestic care, treatments, diagnosis
- Will help those with dementia to live a [better] quality of life
- n/a
- I will integrate stuff re: capacity into my thinking/practice of NP assessment in future
- Better understanding of power of attorney and guardianship will help me discuss those topics better with clients
- I would like to write a proposal for a memory clinic in my area; the information on POA and guardianship was good info
- From the experience/testimony family member with dementia an opener that would facilitate my timely response to referrals associated with early diagnosis of dementia
- Ensuring that there is always collaboration with patients/those with lived experiences
- Larger connections with the Alzheimer's Society; new knowledge of the provincial/national dementia initiative/strategy

- Providing more socialized programs, using little verbal response, more body movements; eating well, exercise
- I feel more aware of the supports and the organizations that can be accessed and am more likely to seek out these resources for clients
- I liked everything
- Awareness of the different programs present that can help assist clients further
- Some initiatives I will follow up on. I don't work in healthcare
- Will read research articles referenced and possibly use content to compliment work with clients (when applicable)
- More focus on residents in LTC homes with dementia and work towards improving quality of life and early diagnosis
- More referrals to Firstlink; more connection to CBTi as per Dr. Xxx
- I now have a few contacts with which I can follow-up with to learn more about collaborations or other opportunities
- Be a part of the problem (dementia) based research soon
- The Alzheimer Society needs to spend more time with RaDAR team to discuss possible collaborations
- Promoting rural and remote health/dementia services and progress through a similar format to Summit in Alberta; Bring researchers and service providers to focus on rural and remote health initiatives related to dementia
- The way I understand the importance of the funding and research
- Keep the rural clients I support more in mind when talking with them (would they be someone who would benefit from attending Summit OR would Summit benefit from them attending)
- Retired
- Early screening and intervention
- Increased collaboration with Alzheimer's Society of Saskatchewan in my research
- Continued involvement with incorporating research evidence based work in our day to day work; value of relationship with RaDAR and SPHERU
- The discussion about legal terminology as it relates to dementia and people with lived experience; The piece about meaningful engagement was also pretty good
- Awareness of people I can ask or programs available (i.e. guardianship, dance)

SUMMIT MEETING - - YOUR OPINION

Elements of the Summit that I liked the most were:

- Rejuvenation and excitement for rural services and possibilities
- Hearing about the innovative treatments/activities that can be used with this population (both the presentations and the posters)
- Everything! Connecting and networking seeing the research that is taking place; Alzheimers Society being present and sharing all their current initiatives
- Dr. Xxx's presentation – stayed with a flow to presentation
- Good presentations, posters
- Networking with others involved in dementia care. Discussion groups that impact research.
- Table discussion – hearing the views of others on a single question was enlightening

- Lived experiences presentation and group discussions
- See "Impact" [re: energized, new ideas re: care, etc.]
- Research; Discussions; RRMC2.0 idea – has potential once kinds are addressed
- Listening to the speakers – it was a great variety of speakers and topics
- All the topics and speakers were very interesting and informative; family stories very effective
- Food was great! Opportunity to network and learn from others working in the area of dementia
- "What's out there"
- The panels, comfort feeding presentation; benefits or negative outcomes of 'locked' dementia units; more information about moderate to severe dementia i.e. resources, engaging them
- Learning of info and resources to take home and use; personal stories to stay motivated to improvements
- Small group discussion was soooo good; the summit presenters were very engaging and there were so many useful topics covered
- Keynotes; Lived experience
- The timelines/breaks were impeccably followed; the entire event being held in one room; having posters displayed during the entire event
- Keynote – dance program; dietician – comfort feeding
- Presentations
- Xxx; family member's presentation; neglect
- Liked all presentations. Particularly enjoyed Dr. Xxx's
- All of it
- Hearing the story of Xxx and his partner was touching and powerful;facilitating discussion groups
- Excellent presentations - especially Dr. Xxx's and Dr. Xxx and Dr. Xxx; Group activities were helpful to hear a range of perspectives
- Topic for discussion this year felt like we were making a large difference with our opinions and comments because we know the complete program that will be coming out
- The small group discussion; the lawyer's notes on POA, etc.
- The part on neglect I found very interesting
- The keynote was great, the dance program was amazing
- Good discussion (small/big group); great ideas!
- Lived experience speakers; RaDAR updates, research updates
- All topics were very interesting
- Food accommodations for certain diet – thank you! Very well organized and informative.
- Networking opportunities; presentations
- Update on the ASOS and the small group discussion as it provides an opportunity to become engaged
- All speakers; the speaker that was a life experience
- It was interesting and informative to hear what people living with dementia have experienced during their journey; I especially enjoyed the presentations from the people directly affected by dementia
- Encouraging public awareness and engaging family members who have/had dementia
- Breakout session and discussion
- Networking; Xxx's work and group discussion
- It was all very good

- Difficult to specify one or two elements! All elements add to my knowledge base in some way and directly/indirectly compliment my day-to-day work with clients.
- Lived experiences; Keynote
- Meeting and conversations with participants
- Lived experience panel; poster sessions; Dr. Xxx's presentation!
- Speaker from family members of dementia people
- Guest speakers
- I loved the talk by Xxx but had trouble connecting the relevance
- Networking opportunities; great pie!
- It was all good
- Group discussion; personal experiences
- Poster session, keynotes and interactive group work; opportunities to share and learn from others
- Talking with peers. Small group discussion session and teamwork and ideas, experiences, knowledge and expertise being forward to a common goal. Unilateral thoughts of the needs for Friends and Staff to carry forward and expand the services to all regions
- The pie?? Lol The many professionals working for a common cause
- Updates on RaDAR Project; On the RaDAR; Personal story – lived experience
- Variety of topics covered; format (great organization!); the venue (and amazing food); also, the meeting was relevant to other provinces!
- The discussion from those with lived experience; Updates on advocacy efforts; Well-timed breaks and overall pacing was great; Food/drink/general environment
- Networking
- Research updates
- Dr. Xxx's presentation

Next year a topic I would include, or a special guest presenter I would invite, would be:

- Recreation therapy – findings in the field, programs used with dementia, etc.
- Dementia prevention research
- As always, recreation therapy and/or educators from U of R with our therapeutic recreation profession and our assessments/treatments we provide as a missing piece to all current research as we have an evidence-based practice; Degree or Certification graduates to present
- Another lived experience of caregivers dealing with family member; or person in early stages of dementia – reaction to their diagnosis and plan for future
- How to help an Alzheimers patient to grieve the loss of a spouse
- Working with undiagnosed dementia in LTC
- Like the lived experience panel
- The lived experiences in terms of ways to support persons living with dementia and their families when dealing with grief of daily losses and living well with dementia
- No comment
- Cognitive Rehab; Lifestyle Modification
- Topics related to responsive behaviours and how to mitigate with meds; new approaches to stimulate/maintain cognitive abilities; how to ensure/enhance the day to day contributions for folks living in LTC – successful approaches currently being practiced

- Inclusion of more participants who need to be involved ie. DIP, WCB – re: awareness and influence; Dementia Strategy specific to LTC!! – especially re: staffing levels needing standards and review – can we hear from the provincial officials on this?!; Research and update on effects of diet in lowering risk and reversing progression of disease?!
- Anticipatory Grief? End-of-life advocacy? More innovation! What can we learn from those leaders
- Xxx; Xxx
- Xxx story was absolutely riveting!; There's lots of info in the Gentle Persuasive Approaches course from the Alzheimer Society – this info has been taught to many front line staff
- Tools to assist as workers to care for people with responsive behavior
- Perhaps the above [Xxx, family member's presentation, neglect]
- Lived experienced presentations are always helpful and informative
- Indigenous dementia care strategies and young onset dementia care strategies (rural/remote); identification of ways and means to work together and spread initiatives and promising practices
- Xxx; Speak on advanced care directives, medical proxy
- I just wanted to know about RRCM
- My granddaughter who wants to go into Medicine!
- Caregiver experience
- More topics on how to have relaxed programming in LTC facilities for clients with various dementia; more topics on communities that have various dementia and how to access
- Education on the different types of dementia and diagnosis of same; education on treatment of dementia – both pharmalogical and non-pharmalogical
- Maybe something with engaging more people in their research
- Stigma within rural communities/how to engage them; denial of disease
- Spread and scale of initiatives that improve experience of care; I would love another invite
- Research related to meaningful engagement of people living with dementia – especially under 65 years of age – in workplaces and other organizations in volunteer roles
- Xxx – Conflict management and intervention
- Behavioural Consultation (BST in Xxx, Dr. Xxx OR Topics related to moderate-severe neurocognitive decline
- Immigrant population and dementia
- Giving feedback on research in small groups was great; more of this? Maybe on a second day?
- Team work, not working in silos – how can we share out information??
- Treatment options – current pharmacology
- I was surprised to hear of the lack of Indigenous patients being seen by the RRMC. I feel like this is a topic worth exploring. Why we're not seeing referrals – an issue with the process itself, or a lack of meaningful engagement with the individual communities? Or maybe cultural differences in care? Would it be helpful to have an Elder, or someone else involved in providing services and working with Indigenous communities to be involved?
- Balance/Mobility aspects; differential diagnoses

Other Comments:

- Excellent event. Thank you.

- I found the small group discussion different because I didn't feel [we] were prepared to address the questions asked
- Thank you for the consistent work and services available to our dementia clients and caregivers as well as presenting all the research and possibilities to assist our dementia clients
- Would have liked to hear the rest of Xxx's story and did not like to see his presentation cut short before he was able to complete his message. Missed opportunity to learn.
- Needed more time for table discussion. We had a lot more to say and discuss! Thank you to organizers – well done!
- Love going green and love that Xxx organized material into one download; the legal trustee should have slides because this is a complicated topic that would have helped me focus
- The group discussion is necessary – I think the topic and project was interesting but should ask participants via mail after Summit further input and intake. It would be nice if it could end at 3 pm for people that need to travel after hours to get home
- Xxx – great organizer/communicator!
- I appreciated the commitment to the time frames on the agenda; lunch was great – kitchen staff rather noisy during clean-up
- Summit as always – great!
- I don't think I understood exactly how Xxx's research project would work and I think that impacted our small group session as there seemed to be a lot of questions on it
- Please don't cut off family members from talking . . . very interesting/beneficial to hear their story; we learn from them and I wanted to hear the whole story
- I appreciated the opportunity to learn more about dementia and projects undertaken in SK to provide services to rural clients
- Food was good, accommodated everyone's dietary concerns; strict timeline, one presenter cut off – good/bad thing
- Fantastic!
- Excellent!
- I was thinking that promoting dementia on social media can also help
- More small group discussion; World Café info sessions; some presentations too long – more interactive networking would be great
- Thank you very much for the opportunity to attend Summit. Xxx and Xxx: I appreciate both of your efforts to connect with all attendees. Once again, an outstanding Summit.
- Reducing paper used and going green is great. Documents on laptop were okay but frustrating to use on phone. Please make more friendly for mobile use.
- Timing, food, everything is excellent
- Could have used more time to have the concept of RRMC2.0 and more time for group discussion
- Hearing people's personal stories was great
- Thanks so much for all your GREAT WORK!!
- Not quite enough time for discussion session
- The accommodations and venue was very good and convenient. Even more convenient (this time of year) would be having the venue in the accommodation hotel
- Invaluable opportunity to interact and network and learn from others to support rural aging and dementia
- Great job to bring together 2019 Summit

- Very good – created awareness in me of the different groups in the province working toward common cause; also different cultural needs though vague but eye-opening
- Great job!
- Very enjoyable again; lots learned

Please rate your <u>satisfaction</u> with the following:	**Extremely Satisfied**	**Very Satisfied**	**Somewhat Satisfied**	**Slightly Satisfied**	**Not at all Satisfied**	**SUM**
**Update on RaDAR projects**	33	40	1	0	0	74
**Morning keynote**	43	27	4	0	0	74
**Small Group session**	21	27.5	18.5	10	1	78
**Research highlights and On the RaDAR panel**	29	39	3	1	0	72
**Alzheimer Society of Saskatchewan update**	30.5	33.5	7	0	0	71
**Lived Experiences panel**	49	15	2	0	0	66
**Materials provided for the meeting**	36	25.5	3.5	1	0	66
**Meeting room, food, and amenities of the [venue]**	48	22	2	2	0	74
**Your opportunity to share your opinions and ideas**	33	36	6	0	0	75
**Your opportunity to increase your knowledge in rural/remote dementia care**	47	25	2	0	0	74
**The value you received for your time today**	45	28	2	0	0	75

Comments written on the above table:

- Re: “research highlights”: new insights
- Re: “materials provided”: had trouble downloading
- Re: “lived experiences”: appreciated the fact that they were willing to share
- Re: “meeting room”: cold
- Re: “morning keynote”: too long
- Re: “small group”: groups were quite large and challenging to hear all participants
- Re: “small group”: felt this was too rushed being explained and not clear; group discussion is always so good, but was fairly unclear and unstructured but could have been so much more rich if we had all received more info from Xxx
