# Supplementary material for: A stakeholder engagement strategy for an ongoing research program in rural dementia care: Stakeholder and researcher perspectives
Source: PLoS One. 2022 Sep 22;17(9):e0274769. doi: 10.1371/journal.pone.0274769 (PMC9499231; doi:10.1371/journal.pone.0274769)
Supplement: S3 File — (PDF) [file pone.0274769.s006.pdf]

Research team member perspectives on the Summit as a stakeholder engagement strategy

Email responses from researchers/trainees (n = 8)

[1] I would say that being involved in Summit has provided me a great model of how to bridge gaps between people working in different sectors of dementia care and research! It is unique from a conference because there is more of a focus on hearing from people working in the field and who have personal experience with dementia. As a trainee, this (and RaDAR's work in general) has allowed me to learn more about how to do research that is community based. It has also allowed me to meet people working in different sectors who I would not otherwise have met.

[2] As a presenter:
- It has definitely made a difference. I see my research in context that it may be used - less 'academic' - focus on what matters - research not just creating knowledge in vacuum rather rigorously documenting facts to influence public policy to solve problems in rural dementia diagnosis and care - help understand my role as a researcher on team.
- Big picture understanding needs and perspectives of parties involved - reveal blind spots - motivating to get to know others involved in rural dementia care or with lived experience as a caregiver or person living with dementia.

Being involved in summit:
- Has changed how I think about working with stakeholders - really appreciate the value of it more - not only do I learn other perspectives, but I also build empathy for the different stakeholder groups. I find myself caring a lot more about rural dementia care on a personal level.

[3] As a speaker, poster presenter, and yearly attendee, the RaDAR Summit has created an opportunity for me to connect with colleagues in similar areas so we may discuss future research endeavors. As a participant, I always take away new research findings to apply in my own clinical practice. If I am struggling with a particular clinical issue, someone at the summit will know who to contact or put me in touch with a team who can directly help me.

[4] For me as a student, Summit had a huge impact seeing such a wide range of attendees that seemed so interconnected and so passionate about improving rural dementia care. I had the opportunity to share my practicum work at Summit and meet so many people working in the dementia field. I sort of already knew I wanted to continue doing dementia research and I feel fortunate to continue my involvement with Summit each year. It is so inspiring to engage regularly with such a committed, diverse group of people and witness the spark of new connections and ideas that drives forward both research and practice. As both a student and a researcher, how could I *not* want to be involved with Summit and this dedicated group of people?

[5] The Rural Dementia Summit is a unique event in many ways. Most meetings of this type which I have previously attended are geared primarily toward psychology trainees, clinicians, researchers, and allied health professionals. The Summit offered an opportunity to present my clinical research project and receive valuable feedback from a much wider range of health service delivery professionals, and more importantly stakeholders, as well as persons with lived dementia experience and their care partners. The

experience of participating in and facilitating a group discussion regarding future directions of the RaDAR team connected multidisciplinary professionals and patients/care partners from across the province as equal partners to identify the research priorities and outcomes.

[6] Engaging in the Summit over the past few years as a student poster presenter has allowed me to see the real potential impact of my research on the healthcare providers who are dealing with real life patients and care issues. This has allowed me to step outside of the lab environment, and gain a more relevant perspective, that is holistic, which made my research seem more relevant and applied. It gave my research more meaning. Also, being able to exchange my thoughts and ideas regarding my research with the Summit attendees/stakeholders has enriched my learning experience regarding some of the current issues in healthcare. Overall, the Summit has helped shape me as a researcher, and has helped to immerse me in real life issues/practice.

[7] In general, I find it very beneficial and thought-provoking to hear stakeholders' perspectives on what I have produced as related (or not sometimes) to their priorities and lived experiences e.g., is what I am presenting out important/of value to them – how so? why? what can I do differently to align with their priorities, perspectives for research?

- perspectives and experiences are unique
- potential to improve quality of my research and effect eventual uptake

The last summit I attended was extremely informative and beneficial for me from both a process and research perspective e.g., we had individuals who had vested interests in the next steps for RaDAR research (and future decisions and evidence to support these decisions) providing the input and their rationales re: the research priority

- the outcome for the research priority was not what I expected based on the discussion at the table I was facilitating, but that is the consensus process
  - research scope defined/refined
  - get buy-in from the ground up

I think this goes without saying – engaging with stakeholders is a step in the right direction in generating research and evidence that is relevant and useful for health care/priority decision-making

[8] Connection: connecting with other researchers, decision makers, patients/families who share a common interest in dementia helps to motivate. Research can be a lonely pursuit in some ways, and the impact can be far-removed from the researcher experience. Summit helps to bring a sense of community and connection, and show the value or importance of the research to providers and pt/families. There is an aspect of validation – that the work we are committing our careers to is valued and useful and important. The encouragement and support given by the pt/families and decision makers helps to maintain the long-term focus of the research.
Connecting with other researchers also helps to stimulate growth, develop ideas, and push your own research further in collaboration.

Goal-oriented nature of regular occurrence of Summit: because the Summit is a regularly scheduled event that reliably occurs every fall, a cycle of research is generated. We work toward the Summit as a goal of having research to present and share for feedback (completed, in progress, ideas in development). Summit creates a deadline to work toward which can be helpful, especially for students and for research ideas that really do require input In order to progress.

Support for trainees and early career researchers: Summit is a huge source of support for trainees - it is a free event that provides a huge value to students (experience presenting research, interacting with pt/fam, providers, decision makers, education and professional development) and encourages them to work in the area of dementia; interest is piqued and the high quality of research profiled serves as a motivator. In terms of early career investigators, the Summit is invaluable. Senior researchers help to mentor, collaboration is given (the established network of the Summit group would take a decade to develop, and it is an asset that is provided to us), and the mechanism of Summit is well established and shared. The ability to gain feedback and collaborate with pt/fam, providers, decision makers is one of the most important aspects, and this model is already well-honed and operates for us. This is invaluable to an early-career researcher. The connections developed at Summit are important as well – increased collaboration and participation in interprofessional research is a benefit.
I can say that my Summit experience really helped me to take the leap into a Phd – I felt like I could do meaningful work that would be of value. And each year I would feel encouraged to work hard on the research; Summit often boosted my spirits and reminded me of why I was pursuing the research I was. As an early career investigator I feel very lucky to work in this collaborative environment and am well-aware of the privilege of working with the pt/families and decision makers and how the regular in-person meetings of Summit are of incredible benefit to me personally.

Leadership: There is a supportive aspect to the Summit that is continual. Summit occurs yearly, but the planning and deliberate connection continue throughout the year. Relationships are fostered and communication (formally and informally) happen regularly. This is all underscored by leadership – without that, Summit and the collaborations wouldn't happen. Someone has to helm this, someone has to have a vision and guide the process. The leadership happens in total collaboration, and requires a long-term commitment.

Progression: collaborating with pt/fam, providers, decision makers pushes the research – sometimes in different directions than anticipated, sometimes faster than anticipated. Knowing that an idea is supported or valued by pt/fam can provide courage to take a research risk or move in a direction that we might not have thought of. It can also help us to let go of ideas that simply wouldn't work in the practical context (insight provided).

Safety/Security: the long term nature helps to develop true relationships beyond the surface. Over time, this helps in terms of trust. Summit becomes a safe place to test out new ideas and brainstorm or problem solve in a trusting, safe environment. I think that we have much more finessed and 'deeper' engagement, and the discussions we have require less time to dive in.

Focus group with team members (n =6)

- Xxx: first few times of participating at Summit and getting feedback – really helped me understand location adaptations to our rural settings and how lack of resources in the field would impact the methods of planned studies. The rural settings are all so different.
- Xxx: for the Xxx study: had to restrict exercise to the type of equipment that could be used in rural site, and therefore had to modify to only one site.
- Xxx: priority setting tasks with Summit participants helped too.
- Xxx: for Xxx discussion, I really got a sense of barriers to adaptations that I hadn't expected (rural/local factors). Frankly *all* the mitigation strategies in the grant application came from discussions in the Summit – every single mitigation strategy in the grant came from the discussion groups at Summit.
- Xxx: mediums of communication – like using telehealth. The methods of how to reach the rural settings always came out from Summit. The rural settings are all so different from each other that the consultation is very appropriate.
- How do you prove greater funding success?
- We definitely get user-relevant questions/comments for discussion
- Xxx: sitting in the groups and listening to the home care nurses discuss travel issues rurally – safety issues around travel (weather and road conditions) and visiting locations (safety issue like personal safety in unknown homes, possible acting-out behaviours of patients being visited), and issues such as who pays for cell phone (personal or work). Due to the feedback from nurses we implemented changes between phase I and phase II of the rural nursing study to address some travel issues. This was an absolutely serendipitous discussion that informed the trajectory of the research.
- Xxx: the interaction/knowledge exchange between users and researchers. Xxx, with your Chair you really changed the focus from knowledge "translation" to "exchange"; towards a more active/interactive process or exchange back and forth. Would be valuable to look at Summit Agendas over-time in addition to the work already done to look at evaluation forms.
- Xxx: I've not been bored any year with content of Summit, because we keep evolving, and it is always interesting. Tongue in cheek comment, perhaps, but sometimes programs/conferences grow stagnant over time, but Summit continues to evolve and change. Now we're bringing in more people with dementia to the meetings and it is making Summit more and more relevant. Xxx suggests that we look at the agendas to review: oh yes, this is what happened at this meeting. Some of the process/development over time for some of the past meetings might be better captured in the *agenda for the day*, vs the *evaluation forms* collected after the event.
- Xxx: Summit keeps us Rural-Centric, and keeping us out of our "Ivory Tower"
- Xxx: looking at the agendas *and* evaluations over time might be a way to tie-in both to each other – the agendas tend to reflect the content of the evaluations from the year before: we evolve the content to meet the needs/feedback reflected in the evaluations.
- Xxx: It's Xxx's leadership overtime.
- It might be good to focus on the PROCESS of how we USE the evaluations – every single thing: down to the weather, the meeting room setup, etc. The feedback we've collected does reflect that participants *see* their comments being valued and put into effect.
- Engaging participants in research: The Xxx meeting pulled in the Xxx group, for example. The thing about Summit is that we often bring in participants for ONE reason and they evolve into something else, become our advocates in other ways – example of Xxx.
- There is a lot of lip-service in engagement research, but Summit seems to create a sense of being

part of a “thing” that is dynamic and moves forward on improvement of access to care for folks with Dementia in rural areas. One year participants provide some ideas in a discussion session, and the next year when the participants come back we’re reporting back on how those comments have already been used to shape an application for funding to address the issues raised.

- Xxx/Xxx: Now Xxx has the funding for system Navigators, which we had proposed as Dementia Advisors.
- Xxx: It’s about keeping goal-directed, even with move from LTC to community-care, Xxx has kept the focus on goal-directed activities. The best leaders are relationship-building leaders. There has to be face-to-face, TH, etc. time to build credibility and relationships over time: which develops trust as a spin-off. With Xxx’s leadership we have people committed at all levels of care – national/provincial/community levels. The terms used in the list of outcomes Xxx circulated at the start of the discussion is so “academic” but these ideas are less so, but just as important.
- Xxx/Xxx: It’s building a culture and a community – community-based culture has been built through these relationships over time. I think that is why Xxx is what it is/has become over time – trajectory of Xxx over time is likely due to interaction with RaDAR team/Summit over time.
- Xxx: I think the whole aspect of the Xxx collaboration could be a separate paper, so much richness over time and their culture of research. Plug for another paper. They’ve become much more rural-focused themselves now. I feel like the Summit having a rural lens has given people permission to consider rural as a whole other area in relation to dementia care. Xxx: It may have been that even well meaning people didn’t see a difference between rural and urban in the past, even those from rural areas themselves, but I feel like that has changed now with Summit.
- Perfect example of taking things back to community: setting up rural memory clinics (with iterative assistance from RaDAR team).
- Xxx: nice that we’re able to show examples of innovations in these communities: what’s going right vs. what’s wrong. And how they’re doing something right.
- Xxx: using “Innovation” is a good word, goes beyond the actual evidence, adapting it to make it work in their settings.
- Xxx: like at this year at Summit: the Xxx folk from Xxx made a comment that it was nice to be surrounded by evidence based research/information that they could also take back to their community and adapt to their context.
- Poster session: lots of people are very interested in the work, hard to trace the impact – but might be worth talking to some of our students about the impact the poster session/Summit has on them. It’s a different interface than academic poster presentation to other researchers: rather its research presentation with local stakeholders who might be looking to adapt research ideas to the concrete.
- Xxx: in my experience the small group discussions at other meetings are begrudgingly done – whereas at Summit it felt like people were really bought-in to the discussion and interested/excited to be sharing their perspective, and it speaks to the history of building the relationship/community of trust over time. In my group it was very good discussion with all sharing: there was a lack of big egos, and rather just a time to share perspectives: everybody was just listening to other. Summit is a large meeting, but feels very intimate at the same time.
- Summit is about increasing capacity and empowerment of stakeholders: ex: Xxx, involvement of first primary health care team, many of the speakers that we have (especially from rural communities) – word is out it’s a *safe place* to bring up ideas to share your voice. People listen to others well.
- Example of Xxx program presentation one year, and then LTC site that adapted it and shared back their experience at the subsequent Summit meeting: building capacity in local communities

to engage with research. Another: this year the dance intervention caught the attention of some of the Xxx folk and they were already discussing ways to adapt it.

- Xxx: The structure of Summit/RaDAR team is very egalitarian, and it *creates* the environment for this to develop. It is my good fortune to be a part of a flat-structure team like this. There is a lot of wasted money in hierarchical structures, in my opinion.
