# Supplementary material for: A stakeholder engagement strategy for an ongoing research program in rural dementia care: Stakeholder and researcher perspectives
Source: PLoS One. 2022 Sep 22;17(9):e0274769. doi: 10.1371/journal.pone.0274769 (PMC9499231; doi:10.1371/journal.pone.0274769)
Supplement: Attachment — Submitted filename: Response to Reviews (Aug 14, 2022) .docx [file pone.0274769.s007.docx]

**RESPONSE TO REVIEWS**

The authors would like to thank the reviewers for their thoughtful and helpful comments on the manuscript. We appreciate the opportunity to revise the paper and hope that we have addressed their recommendations to their satisfaction.

**A. Journal Requirements. When submitting your revision, we need you to address these additional requirements:**

**1. Please ensure that your manuscript meets PLOS ONE's style requirements, including those for file naming.**

This has been done.

**2. Please provide additional details regarding participant consent. …If the need for consent was waived by the ethics committee, please include this information.**

This has been done. Consent was waived by the ethics committee because the study was deemed program evaluation. However, Summit participants were informed that the evaluations could be used for publications. The evaluations were completed anonymously.

**3. We note that the grant information you provided in the ‘Funding Information’ and ‘Financial Disclosure’ do not match. When you resubmit, please ensure that you provide the correct grant numbers for the awards you received in the ‘Funding Information’ section.**

This was done. The information is included in the cover letter as requested.

**4. We note that you have provided funding information that is not currently declared in your Funding Statement. Funding information should not appear in the Acknowledgments or other areas of your manuscript. Please remove any funding-related text from the manuscript and let us know how you would like to update your Funding Statement. Please include your amended statements within your cover letter.**

We have removed funding information from the Acknowledgements and included it in the cover letter.

**5. In your Data Availability statement, you have not specified where the minimal data set underlying the results can be found. Upon re-submitting, please upload your study’s minimal underlying data set as either Supporting Information files or to a stable, public repository and include the relevant URLs, DOIs, or accession numbers within your revised cover letter. We will update your Data Availability statement.**

The original submission stated that the data were provided as Supporting Information Files attached to the submission. This information is included in the cover letter.

**7. Please amend your list of authors on the manuscript to ensure that each author is linked to an affiliation.**

All authors have an affiliation except for a family caregiver (EP) who does not have an affiliation as she is retired and not linked to any organization.

**B. Reviewers' comments:**

**Reviewer #1**:

**This is generally a well-written manuscript that reports on the laudable goal of stimulating participatory research involving a broad range of stakeholders involved in rural dementia research and care over many years.**

**My major concern is that a substantial proportion of the manuscript reads like an organisational report, rather than the product of systematic research. The paper would benefit from a clear early section about the research methodology and how this was designed to answer specific research questions, with some justification/rationale around the mixed methods approach.**

Thank you for these comments. We agree that a statement about the methodology should have been included and have added this information at the beginning of the Methods section (Page 8, starting line 195). The more organizational aspects of the Summit in tables have been moved to Supporting Information files (see below).

**I would suggest the authors have a close look at sections of the paper such as Table 1 and 2 as to whether they fit in a research report. Table 3 may benefit with linkages to relevant research papers as well as outcomes that affect delivery of care in a rural setting.**

Based on this suggestion, and the recommendation of Reviewer 2 to remove some of the detail and reduce the length of the manuscript, we have removed Tables 1 – 3 and Box 2 from the manuscript. These tables are now included as Supporting Information files S1 (Summit components and their purpose), S2 (Example of Summit small group engagement sessions), S3 (how Summit differs from a typical conference), S4 (Interactive small group sessions focus and outcomes). Because of the gap in the engagement literature regarding the structure and processes of engagement strategies for ongoing research programs, we feel it is important that readers have access to this information. These structure and processes evolved in response to stakeholder feedback and are linked to stakeholder satisfaction with the Summit and the outcomes reported in the thematic analysis. In Table S2 the term “outcome” in one of the headings was changed to “purpose” to more accurately reflect the content of that column.

**I am also concerned that some of the data presented, particularly evaluation data, was deemed exempt from ethics committee consideration. Was this because this was for internal evaluation purposes initially and/or were participants aware that data may be reported?**

Yes the University ethics committee deemed the evaluations exempt from ethics review because it was collected for program improvement. Participants were informed that the evaluations may be used for reports and publications.

**It would be useful for the authors to consider issues of bias and motivation in their reporting. Clearly, those attending would have been highly motivated in this area? In addition, it should be made clear whether it is the RaDAR team that comprises the authorship of the article. Were any of the analyses conducted by researchers who were not engaged in RaDAR or the summit? I note the absence of any negative themes or quotes, which may well be the case, but it would be good to hear more about this paper’s research approach to reduce bias.**

Summit participants were invited based on their interest and expertise in dementia care, or were self-identified individuals who requested to attend, so they were highly motivated. The authors are RaDAR researchers, except for a family caregiver and rural nurse practitioner who have both had a long association with the RaDAR team and are regular Summit attendees. Many Summit participants attend every year and may be more comfortable reporting where improvements could be made, but it is possible that new participants would be less likely to make negative comments. Most of the “negative“ findings were related to organizational aspects such as Summit dates or start and end times, keeping to schedule so there is time for discussion, suggesting additional stakeholder groups to invite, etc, and these are noted in the Challenges section. Potential bias in the analysis was mitigated by the team approach to the analysis of themes, and inclusion of non-RaDAR members.

**Reviewer #2:**

**The work reported in this paper is very informative and of high relevance. The paper is very well and clearly written. However, I would strongly recommend the authors to review the paper and reduce it substantially in length. Please consider a more concise writing style and review whether the amount of detail provided in the current version of the manuscript is really necessary for the optimal communication of the message. As I mention, given the importance of the message, it would be best if the length and amount of detail provided does not discourage potentially interested readers.**

Thank you for your comments, and the recommendation for a more concise paper. As noted under Reviewer 1’s second point above, we have removed 8 pages of detail by relocating Tables 1-3 and Box 1 from the text to Supporting Information Files and removing some text throughout the manuscript. We agree that these changes make the paper more accessible, while still providing interested readers with the specifics regarding Summit organization and content in the optional Supporting Information files.
